# Clinical, tumor, and product features associated with outcomes after axicabtagene ciloleucel therapy in follicular lymphoma

**DOI:** 10.1172/JCI181893

**Published:** 2025-08-15

**Authors:** Soumya Poddar, Jiali Yan, Gayatri Tiwari, Darawan Rinchai, Justin Budka, Wangshu Zhang, Weixin Peng, Shruti Salunkhe, Madison Davis, Qinghua Song, Sara Beygi, Harry Miao, Mike Mattie, Rhine S. Shen, Caron A. Jacobson, Davide Bedognetti, Simone Filosto, Sattva S. Neelapu

**Affiliations:** 1 Kite, a Gilead Company, Santa Monica, California, USA.; 2 Dana-Farber Cancer Institute, Boston, Massachusetts, USA.; 3 The University of Texas MD Anderson Cancer Center, Houston, Texas, USA.

**Keywords:** Hematology, Oncology, Cytokines, Lymphomas, T cells

## Abstract

**BACKGROUND:**

Axicabtagene ciloleucel (axi-cel), anti-CD19 chimeric antigen receptor (CAR) T cell therapy, demonstrated remarkable efficacy with manageable toxicity in relapsed/refractory indolent B cell lymphomas in the ZUMA-5 trial.

**METHODS:**

Here, we report associations of product attributes, serum biomarkers, clinical features, and tumor characteristics with outcome in 124 patients with follicular lymphoma (FL).

**RESULTS:**

In univariate and multivariate analyses, pretreatment inflammatory markers, including TNF-α and IL-12p40, as well as total metabolic tumor volume (TMTV), associated with disease progression. Conversely, T-naive–like product phenotype associated with improved outcome, particularly in patients with high TMTV. These covariates improved risk stratification when combined with the FL International Prognostic Index. Postinfusion, CAR T cell expansion associated with improved outcome, while serum inflammatory and immunomodulatory markers, including TNF-α, associated with disease progression and occurrence of high-grade cytokine release syndrome or neurologic events, presenting targets to improve the therapeutic index of axi-cel in FL. Tumor gene expression profiling revealed that both type I and II IFN signaling associated with disease progression and higher expression of T cell exhaustion markers, including TIM3 and LAG3. Pre- or posttreatment CD19 expression on tumor was not associated with outcome.

**CONCLUSION:**

These findings offer insights into mechanisms of resistance and toxicity, risk stratification, and strategies for development of next generation CAR-T approaches.

**TRIAL REGISTRATION:**

ClinicalTrials.gov NCT03105336.

**FUNDING:**

Kite, a Gilead Company.

## Abstract 2

This analysis of translational biomarkers and clinical covariates identified key determinants of durable response and toxicity with CAR T-cell therapy in patients with follicular lymphoma.

## Graphical abstract

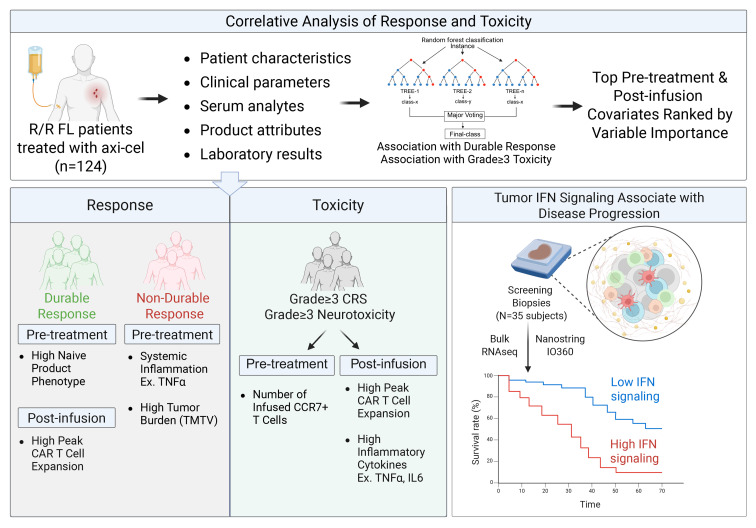

## Introduction

Follicular lymphoma (FL) is the second-most common subtype of adult B cell non-Hodgkin lymphoma (NHL). Most patients with FL have an indolent disease course, but the disease is considered incurable with patients experiencing frequent relapses and eventual development of resistance to current therapies. The prognosis is especially poor in the 20% of patients with FL who develop progression of disease within 24 months after first-line immonochemotherapy (POD24) (1, 2). Axicabtagene ciloleucel (axi-cel), an autologous anti-CD19 chimeric antigen receptor (CAR) T cell therapy, demonstrated high overall response rate (ORR; 94%), complete response rate (CRR; 79%), and durability (36-month progression-free survival [PFS] rate of 54%) in adult patients with relapsed/refractory (r/r) FL on the ZUMA-5 phase 2, multicenter, single-arm study (3, 4). Responses were similar in patients with FL treated with axi-cel with high-risk disease features, including POD24 (3). Axi-cel–associated toxicities, including cytokine release syndrome (CRS) and neurologic events (NEs) were manageable. These results led to the approval of axi-cel for the treatment of adults with r/r FL after 2 lines of systemic therapy.

**Figure 1:**
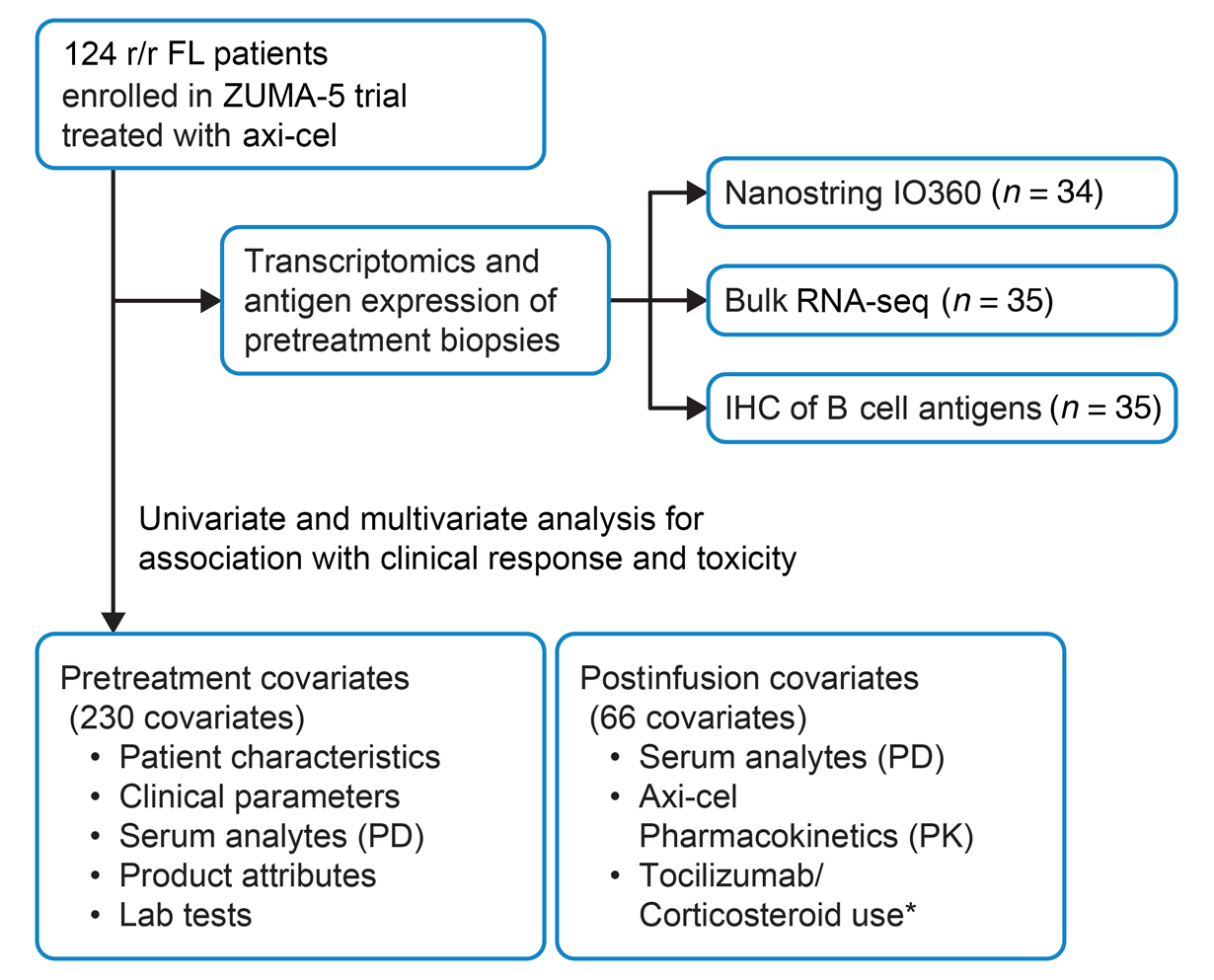
Study design for identification of covariates associated with clinical response and toxicity. *Tocilizumab/Corticosteroid use were not included for association with toxicity.

While the response rates were high and adverse effects were manageable with axi-cel in FL, identification of biomarkers associated with durable responses, resistance, and high-grade toxicity could help select patients most appropriate for this therapy in the future and lead to development of novel approaches to further enhance the safety and efficacy of this therapy. Previously, univariate and multivariate cox analysis of the ELARA study, employing tisagenleclucel (tisa-cel) in r/r FL patients showed that elevated tumor burden [TB; total metabolic tumor volume (TMTV) ≥ 240 cm^3^] at baseline (prelymphodepleting chemotherapy [pre-LD]), lower number of naive CD8^+^ T cells and more than 4 nodal areas at inclusion were clinical factors that correlated significantly with disease progression (5). FL International Prognostic Index (FLIPI) score was not tested in the context of CART cell therapy in patients with r/r FL. Here, we analyzed host, tumor, and CAR-T product attributes of patients with FL treated on the ZUMA-5 study and performed univariate and multivariate analyses to identify pre- and posttreatment biomarkers associated with durable responses and/or high-grade CRS and NEs (Figure 1).

## Results

### Durable response associated with low systemic inflammation, low TMTV and high naive T cell product phenotype.

We conducted an analysis of 230 pretreatment variables (Supplemental Table 1; supplemental material available online with this article; https://doi.org/10.1172/JCI181893DS1) at 2 different timepoints: baseline (most recent readout prior to initiation of lymphodepleting chemotherapy, LD) and day 0 (after LD prior to CAR T cell infusion) in 124 patients with r/r FL treated with axi-cel in the ZUMA-5 study (see Methods for details). This included patient characteristics, clinical parameters (e.g., number of prior lines of therapy, age, bulky disease, etc.), baseline and day 0 serum pharmacodynamic readouts, axi-cel product attributes, and clinical laboratory test results (e.g., hemoglobin, albumin, aspartate aminotransferase [AST]). The analysis comprised univariate screening (Supplemental Figure 1), feature selection, and multivariate analysis using machine learning. Variable importance (VI) was determined separately for PFS and binary endpoint (ongoing response versus nonongoing response by data cut off) using a random forest model. Some covariates were excluded from the VI analysis because of high collinearity. For instance, percent of T-naive in the product strongly correlated with total number of infused T-naive; likewise, the CAR-T cell peak strongly correlated with CAR-T cell AUC. The full list of covariates included and excluded in the multivariate analysis is presented in Supplemental Table 2.

**Figure 2:**
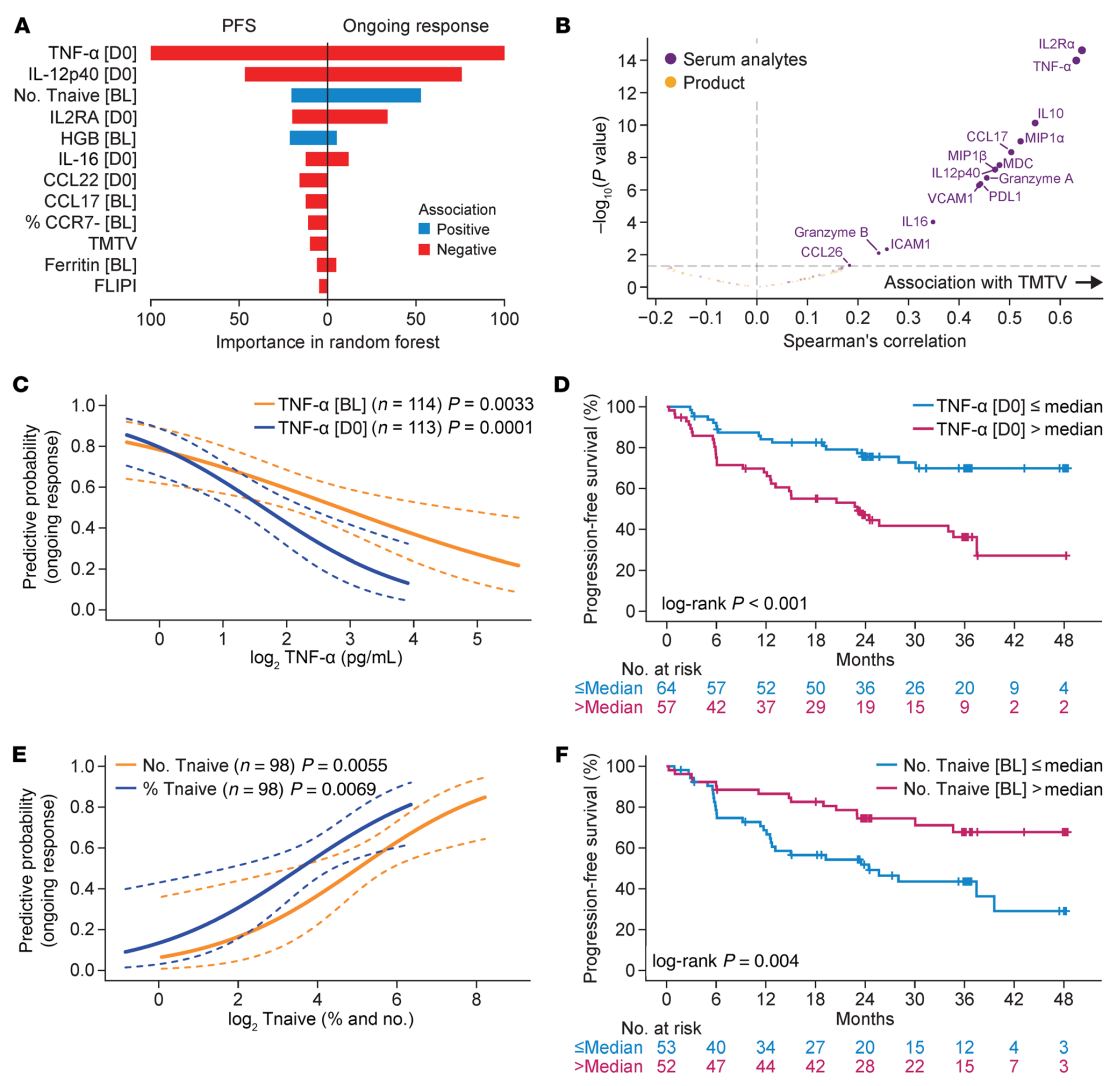
Multivariate analysis of pretreatment covariates identified TNF-α, systemic inflammation, naive product phenotype, and total metabolic tumor volume to be associated with clinical response. (**A**) Variable importance of covariates associated with ongoing response and PFS. (**B**) Correlation of baseline serum analytes and product attributes with TMTV (**C**) Predictive probability (95% CI) of ongoing response by baseline and day 0 TNF-α; (**D**) KM estimated PFS by day 0 TNF-α above or below median. (**E**) Predictive probability (95% CI) of ongoing response by the total number and percentage of infused naive T cells in axi-cel product. (**F**) KM estimated PFS by total number of naive T cells above or below median. FLIPI, follicular lymphoma international prognostic index; HGB, hemoglobin; PFS, progression-free survival; TMTV, total metabolic tumor volume; Tnaive, naive T cells.

Among pretreatment covariates at both baseline and day 0, inflammatory markers TNF-α, IL-12p40 and IL-2Rα associated with disease progression (lack of ongoing response or shorter PFS). Day 0 TNF-α showed the strongest association with disease progression among all pretreatment covariates, ranked by VI (Figure 2A). The total number of infused naive T cells (defined as expressing surface markers CCR7 and CD45RA) and hemoglobin showed association with improved outcome (Figure 2A), while serum chemokines CCL17, CCL22, immune-modulating IL-16, TMTV, and percentage of effector and effector memory T cells in the product (% CCR7-) associated with disease relapse and shorter PFS. FLIPI score also associated with shorter PFS (Figure 2A). Supplemental Figure 1C shows the patient-level distribution of the pretreatment covariates identified in the multivariate model.

Univariate analyses corroborated the above observations with the sole exception of CCL22 (Supplemental Figure 1A and Supplemental Figure 2, C–F and A–I) and provided additional insights. Lower levels of baseline or day 0 serum TNF-α and product enrichment in naive T cells (% of T cells or total number of infused naive T cells) could enhance outcomes in patients with r/r FL treated with axi-cel (Figure 2, C–F). Baseline hemoglobin levels associated with improved PFS with a median cutoff of 12.45 g/dL (Supplemental Figure 2A), which is consistent with levels of less than 12 g/dL being considered a poor prognostic factor for patients with FL (6, 7). Baseline serum Ferritin showed trends of negative association with ongoing response (Supplemental Figure 2F).

In lymphoma, tumor burden is traditionally assessed by the sum of product diameters (SPD) of target lesions (8). However, recent studies have proposed a superior prognostic value of TMTV measured by FDG PET-CT scans in FL and diffuse large B cell lymphoma (LBCL), compared with SPD (9–12). In our analysis, TMTV was a stronger predictor of ongoing response (*P* = 0.049) than SPD (*P* = 0.081), although SPD and TMTV were moderately correlated (Supplemental Figure 3A). TMTV associated with worse PFS with a median cutoff of 403.7 mL (Supplemental Figure 3B), and optimal cutoff of 438.5 mL (Supplemental Figure 3C), while it only showed a trend of negative association with cutoff of 240 mL (Supplemental Figure 3D), which was previously used in ELARA study (5). Lactate dehydrogenase (LDH), which has been used as a surrogate for tumor burden, was not associated with ongoing response (Supplemental Figure 3E) or TMTV (Supplemental Figure 3F). Several baseline serum analytes, including IL-2Rα, TNF-α, CCL17 (TARC), CCL22 (MDC) and IL-12p40, which were associated with each other (Supplemental Figure 1B) and disease progression, demonstrated correlations with TMTV (Figure 2B).

### Durable response associated with postinfusion CAR T cell expansion and inflammatory serum analytes.

**Figure 3:**
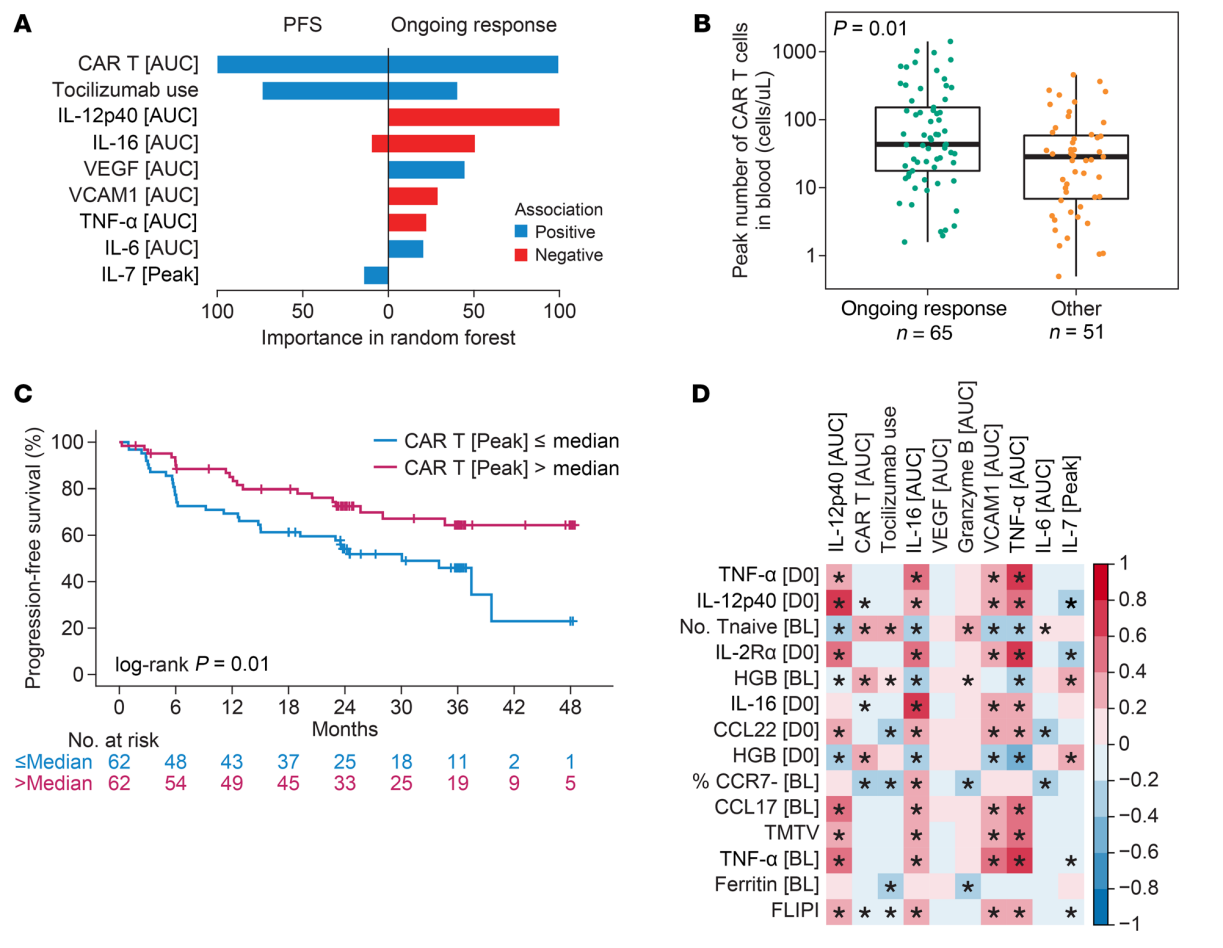
Multivariate analysis of postinfusion covariates identified CAR T cell peak expansion and sustained systemic inflammation to be associated with clinical response. (**A**) Variable importance of postinfusion covariates associated with ongoing response and PFS. (**B**) Box plot of peak CAR T cell levels among patients with ongoing response versus relapsed and nonresponders. (**C**) KM estimated PFS by peak CAR T cells above and below median. (**D**) Spearman correlation of pretreatment and postinfusion covariates identified in multivariate analysis. Correlations with *P* value <0.05 are indicated by *. CAR, chimeric antigen receptor; MIP, macrophage inflammatory protein; PFS, progression-free survival; SAA, serum amyloid A; TMTV, total metabolic tumor volume; HGB, hemoglobin; FLIPI, follicular lymphoma International prognostic index; Tnaive, naive T cells

We conducted multivariate analyses involving 66 postinfusion covariates encompassing serum analytes, tocilizumab/corticosteroid usage, and CAR T cell expansion metrics in blood (Supplemental Table 3). Some covariates were excluded because of high collinearity (e.g., Peak and AUC of several postinfusion variables like IL-6, IL-2, etc. showed spearman correlation R > 0.9; only AUC was included in multivariate analysis for those cases). Per VI analysis, the top-ranking covariates associated with durable response were CAR T cell AUC, tocilizumab use, serum VEGF AUC, IL-6 AUC, and IL-7 Peak level (Figure 3A). Conversely, IL-12p40 AUC, IL-16 AUC, VCAM1 AUC, and TNF-α AUC associated with relapse (lack of ongoing response; Figure 3A). Supplemental Figure 4A shows the patient-level distribution of the postinfusion covariates identified in the multivariate model. The positive correlation between tocilizumab use and durable response (Figure 3A), is likely due to its administration in patients experiencing CRS and NE, which are associated with CAR T cell expansion (13).

Univariate analyses corroborated the above and provided additional insights (Supplemental Figure 5, A and B). CAR T cell peak levels exhibited association with improved ongoing response (Figure 3B) and longer PFS (Figure 3C). Peak CAR T cell levels normalized to TMTV showed an even stronger association with ongoing response (Supplemental Figure 4B). Further, in univariate analysis, positive associations were observed between ongoing response and serum IL1RA, amyloid A, IFN-γ, and IL-6, while MIP1-α associated negatively with outcome (Supplemental Figure 5A).

Several covariates assessed at baseline or day 0, before CAR T cell infusion, were associated with select postinfusion covariates. TNF-α at baseline and day 0, CCL22 on day 0, CCL17 at baseline, IL16 on day 0, TMTV, and FLIPI score associated positively with postinfusion AUC of IL-12p40, TNF-α and IL-16. Whereas the number of naive T cells in the product and hemoglobin levels at baseline and day 0 associated positively with CAR T cell AUC (Figure 3D).

When pretreatment and postinfusion variables were jointly analyzed for their association with outcome, pretreatment inflammatory cytokines and naive T cells in axi-cel product ranked higher by VI compared with CAR T cell expansion and TMTV for association with durable response (Supplemental Table 4).

### Tumor IFN signaling is associated with increased risk of disease progression.

To investigate factors in the tumor microenvironment (TME) influencing outcome, we conducted gene expression analyses of pretreatment tumor biopsies (collected at initial diagnosis or before LD). We utilized the NanoString PanCancer IO360 panel to assess predefined gene expression signatures (GES) in 34 patients with FL and further performed bulk RNA-seq analysis in 35 patients (14). 30 patients were common between the Nanostring and RNA-seq datasets.

Nanostring IO360 B cell signature previously showed a strong association with efficacy (event-free survival [EFS] and ongoing response) in second-line (2L) LBCL patients on ZUMA-7 study (15). In this study, B cell signature did not correlate with PFS or ongoing response (Supplemental Figure 6, A and B). A significantly higher B cell signature in r/r FL compared with 2L LBCL (*P <* 0.00001, Supplemental Figure 6C) could explain its lack of impact on outcomes in FL, as B cell signature under or equal to the median would not be representative of limiting factors in r/r FL tumors. In fact, all but 2 patients in ZUMA-5 had B cell signature values higher than the median value observed in ZUMA-7 (Supplemental Figure 6C). Gene expression of *CD19* (Supplemental Figure 6D), which is one of the genes included in the B cell signature, along with CD19 protein expression (Supplemental Figure 6E) also did not associate with PFS in patients with r/r FL, whereas EFS was improved in 2L LBCL (ZUMA-7) with high CD19 expression (15). Analysis of paired biopsies, at pretreatment compared with time of disease progression, showed preservation of CD19 and CD20 expression at progression (*n* = 11 and 9, respectively) except for one case for CD19 (Supplemental Figure 7A). These findings suggest pretreatment antigen levels and loss of CD19 expression after CAR T cell treatment are not major drivers of CAR-T cell resistance in r/r FL.

**Figure 4:**
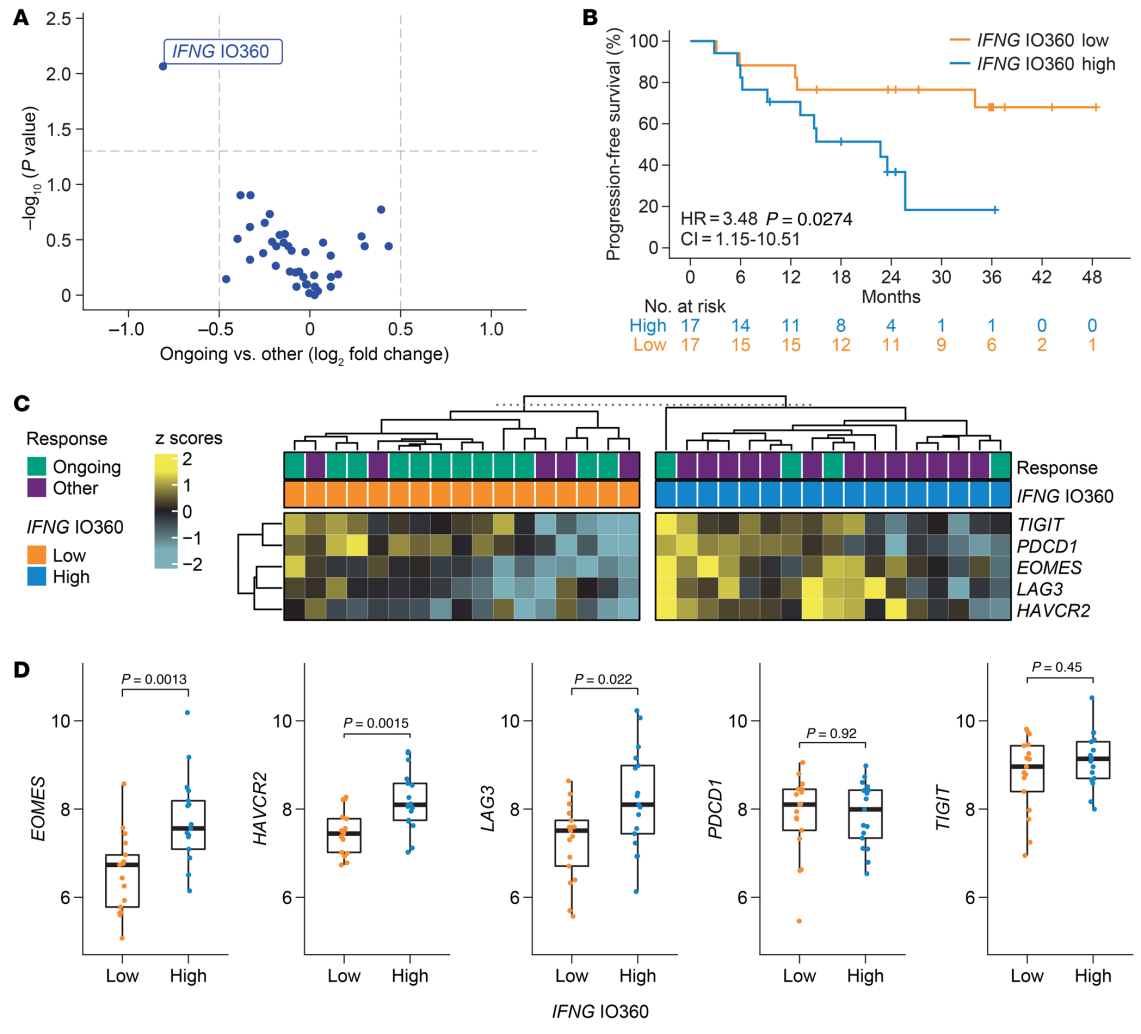
Tumor IFN signaling and T cell inhibitory ligands are associated with lack of clinical response. (**A**) Differential association of Nanostring IO360 scores with ongoing response (Wilcoxon test) (**B**) KM estimated PFS by median IFNG IO360 score. (**C**) Heatmap comparing expression of T cell ligands between patients with high versus low IFNG IO360. (**D**) Boxplots comparing expression (log_2_ normalized counts) of representative T cell inhibitory ligands between IFNG IO360 high versus low. Wilcoxon tests were done to compare expression between ongoing responder versus others. KM, Kaplan-Meier; PFS, progression-free survival.

Out of 40 Nanostring predefined GES consisting of more than 1 gene and Immunosign 21 (14) (Supplemental Table 5), *IFNG* IO360 was the only GES with significantly higher values in patients who experienced disease progression (relapsed and nonresponders), compared with those with ongoing responses (*P <* 0.05; Figure 4A and Supplemental Figure 8A). *IFNG* IO360 also associated negatively with PFS (Figure 4B and Supplemental Figure 8B). The *IFNG* IO360 signature included *STAT1, CXCL9, CXCL10*, and *CXCL11* genes. While *STAT1*, *CXCL10*, and *CXCL11* can be induced by both type I (IFN-α/β) and type II (IFN-γ) IFN, *CXCL9* is specifically induced by IFN-γ (16). *CXCL9* had the greatest fold decrease in ongoing responder versus nonresponder and relapsed patients (fold decreases of 62.8%, 43.7%, 26.0%, and 28.45%, for *CXCL9, CXL10, CXCL11*, and *STAT1* respectively (Supplemental Figure 8, C and D).

To better understand the role of IFN signaling in FL tumors, we interrogated the expression of T cell ligands previously identified to be controlled by IFN signaling in solid tumors (9) and in LBCL (17). Blocking tumor IFN signaling can improve immune checkpoint therapy in solid tumors through restoration of CD8^+^ T cell–mediated tumor killing (10). Patients with FL with high *IFNG* IO360 signature had significantly higher expression of T cell inhibitory ligands, including *LAG3*, *TIM3 (HAVCR2)*, and *EOMES* transcription factor associated with exhaustion (Figure 4, C and D) (11).

**Figure 5:**
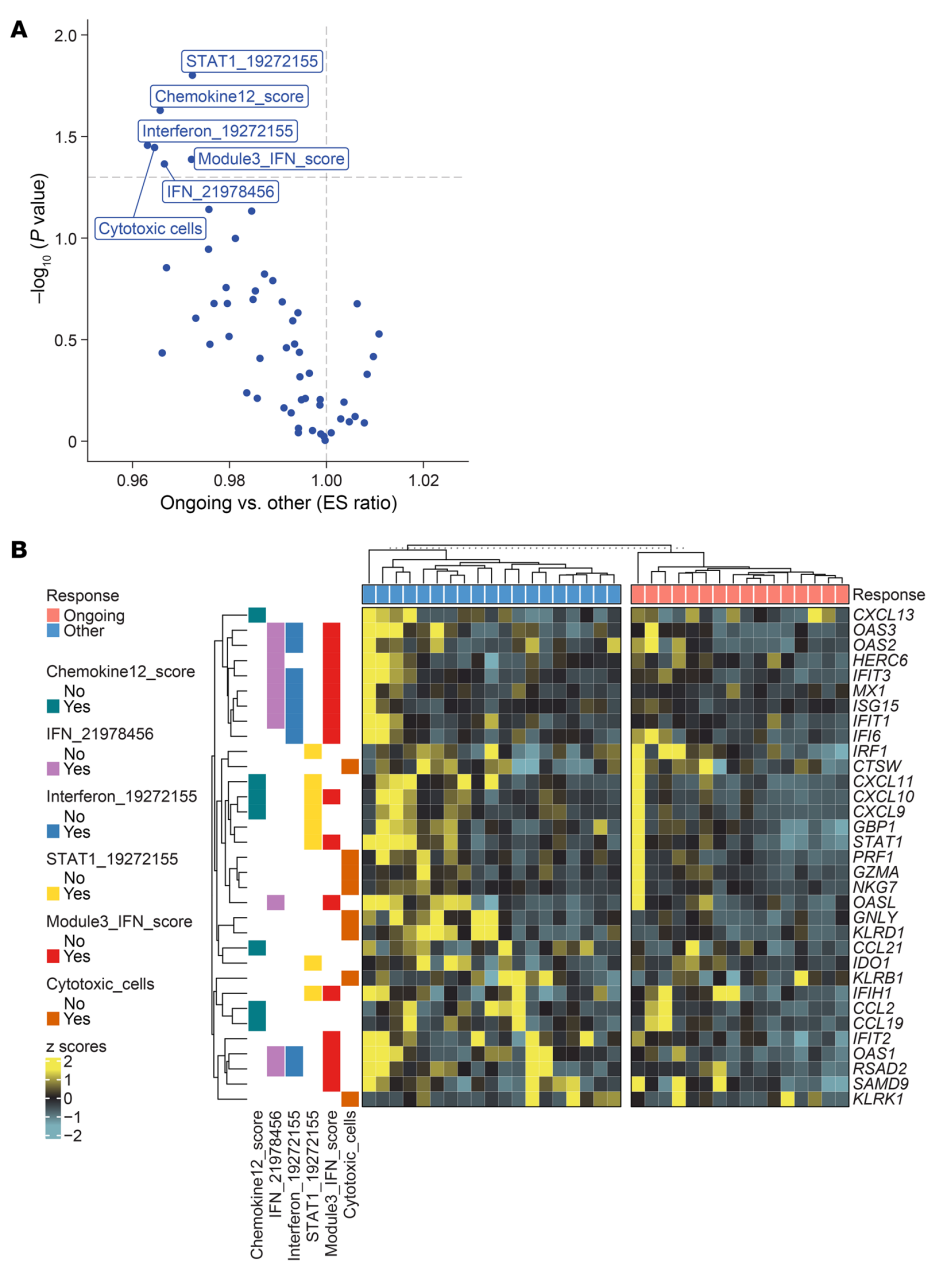
Differential enrichment scores using RNA-seq dataset highlights IFN signaling to be associated with disease progression. (**A**) Volcano plot showing differential enrichment scores between ongoing responders versus relapsed and nonresponders. (**B**) Heatmap comparing expression of the genes included in the presented signatures between ongoing responders and others (nonresponders and relapsed).

We employed RNA-seq for technical validation of the results obtained with Nanostring IO360 panel and investigated TME signatures defined using RNA-seq gene expression in solid tumors (12). Differentially expressed genes (DEG) by RNA-seq identified IFN-stimulated genes (ISGs) to be associated with disease progression (Supplemental Figure 9A). Gene expression of *CXCL9, CXCL10, CXCL11, STAT1* and other ISGs by RNA-seq recapitulated the nanostring results (Supplemental Figure 9B). 56 well-characterized gene signatures/immune traits (Supplemental Table 6) that reflect specific tumor-related pathways (including T/B cell response, TGF-β signaling, IFN signaling, and others) in solid tumors (12) were also investigated for association with clinical outcomes. IFN-related signatures STAT1_19272155, Interferon_19272155, Module3_IFN_score and IFN_21978456, as well as Chemokine_12_score and cytotoxic cells were associated with disease progression (Figure 5A). While cytotoxic cells are generally associated with anti-tumor immunity, the enrichment of such gene expression signature (named “cytotoxic cells”) in tumors from relapsed and nonresponder patients suggests a dysfunctional or exhausted state of these “cytotoxic cells” rather than an effective antitumor role. This finding is consistent with the literature indicating that chronic IFN signaling within the tumor microenvironment can drive T cell dysfunction and exhaustion, ultimately limiting tumor control (17), i.e., it is suggestive of an immune cytotoxic response that has failed and is no longer functional. The individual genes representative of these signatures overall showed increased expression in tumors from relapsed and nonresponders (Figure 5B and Supplemental Figure 9B).

**Figure 6:**
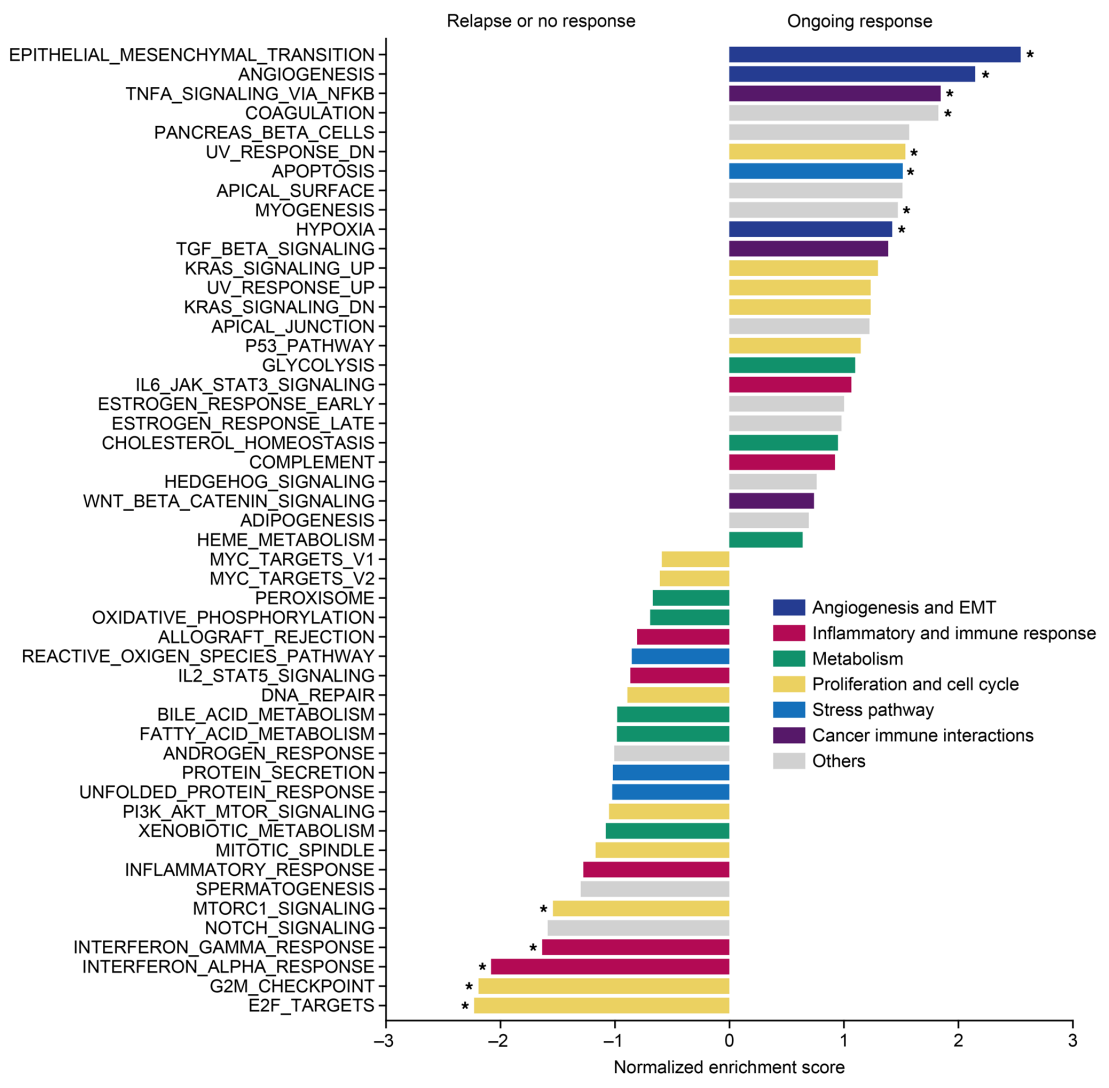
Gene set enrichment analysis (GSEA) using RNA-seq gene expression of pretreatment biopsies. Hallmark pathway gene set show pathways enriched in ongoing responders versus relapsed and nonresponders. Enrichment of tumor IFN-γ (Type II) and tumor IFN-α (Type I) signatures are observed in relapsed and nonresponders. *P < 0.05.

With RNA-seq and GSEA analyses, enrichment scores for five hallmark pathways were associated with disease progression. This included IFN-α and IFN-γ response pathways, confirming findings from nanostring IO360. Conversely, 8 hallmark pathways, including “TNF-α signaling via NF-κB” were linked to durable response (Figure 6). Gene lists from the hallmark pathways associated with outcome (by GSEA) were used to create gene expression signatures via GSVA, which were then correlated to nanostring IO360 signatures (Supplemental Figure 10A).

The IFN response pathways were associated with cytotoxicity, tumor inflammatory signaling, lymphoid and myeloid signatures, and CD8^+^ T cell exhaustion indicative of a very inflamed TME where T cells are no longer functional. TNF-α signaling via NF-κB was associated with APC, myeloid inflammation alongside stroma and T cells, but not with CD8^+^ T cell exhaustion, a functional T cell response supported by antigen-presenting cells (APCs) and vasculature. Notably, the tumor TNF-α signaling via NF-κB pathway showed negative correlation with serum TNF-α levels (Supplemental Figure 10B). Epithelial mesenchymal transition, angiogenesis, and other hallmark pathways that were associated with durable response by GSEA and showed associations with IO360 signatures that were similar to those observed for TNF-α signaling via NFκB.

G2M checkpoint and E2F target pathways negatively associated with T cell, NK, and vasculature signatures, possibly representing a cold and “immune desert” TME. mTORC1 signaling pathway was associated with glycolytic activity, a marker of poor prognosis in LBCL (15), which may indicate a TME that is metabolically restrictive to T cell function, consistent with findings in solid tumors (18).

These results support the hypothesis that excessive tumor IFN signaling is linked with an immunosuppressive tumor microenvironment and exhausted intratumoral T cells, limiting efficacy of CAR-T cell therapy.

### Improved risk stratification by combining FLIPI with serum TNF-α, TMTV or naive product phenotype.

**Figure 7:**
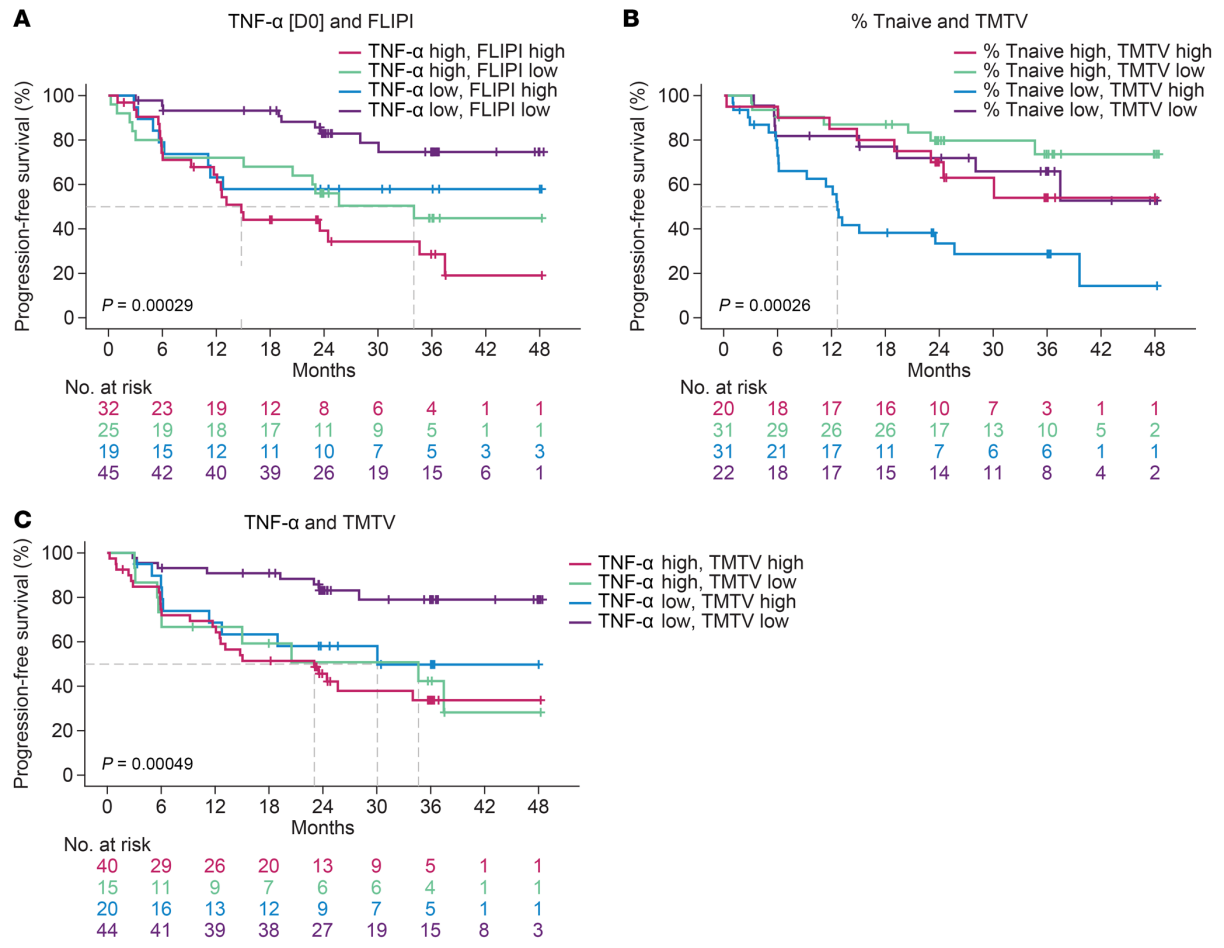
Risk stratification of patients with FL using preinfusion covariates identified in multivariate analysis. (**A**) KM-estimated PFS by median of pretreatment TNF-α in combination with FLIPI score. (**B**) KM-estimated PFS by medians of baseline TMTV and percent of naive T cells in the product. (**C**) KM-estimated PFS by median of day 0 TNF-α in combination with baseline TMTV. FLIPI, follicular lymphoma International Prognostic Index; KM, Kaplan Meier; PFS, progression-free survival; TMTV, total metabolic tumor volume. FLIPI high: FLIPI score > 2; FLIPI low: FLIPI score ≤ 2; Medians were used for all other variables for high versus low.

Among the pretreatment covariates ranked by multivariate analysis, preinfusion TNF-α, product T-naive phenotype, TMTV, and FLIPI score ranked as top ones in their respective categories of serum analytes, product attributes, tumor features, and clinical biomarkers. Hence, we investigated the value of combining these features to improve prediction of outcome. FLIPI score stratified ZUMA-5 patients with FL between FLIPI high (> 2) versus FLIPI low (≤ 2) scores (Supplemental Figure 2E). FLIPI high and FLIPI low patients could be further risk-stratified into 2 subgroups when accounting for levels (> versus ≤ median) of TNF-α (Figure 7A), percent of product naive T cells (Supplemental Figure 11A), or TMTV (Supplemental Figure 11B). Notably, we observed that naive product T cells associated (*P <* 0.05) with improved outcome only in patients with relatively high (> median) TMTV (Figure 7B). Conversely, low levels of TNF-α at day 0 associated with improved outcome only in patients with lower TMTV (Figure 7C).

### Pretreatment product attributes and posttreatment serum inflammatory markers associated with high-grade CRS and NE.

Of the 124 patients with r/r FL in ZUMA5, 19 (15%) experienced grade ≥ 3 neurologic events (NE), and only 8 patients (6%) developed grade ≥ 3 cytokine release syndrome (CRS). A robust association (χ^2^ test *P* = 0.021) was observed between grade ≥ 3 NE and grade ≥ 3 CRS in patients with r/r FL (Supplemental Figure 12A). We conducted univariate and multivariate analysis with 294 pretreatment and postinfusion covariates encompassing clinical parameters, patient demographics, CAR T cell expansion, serum analytes, tumor burden (SPD and TMTV), product attributes, and baseline laboratory values to identify factors associated with high-grade (grade ≥ 3) CRS and/or NE. For the multivariate analysis, the covariates were ranked by VI using random forest analysis.

**Figure 8:**
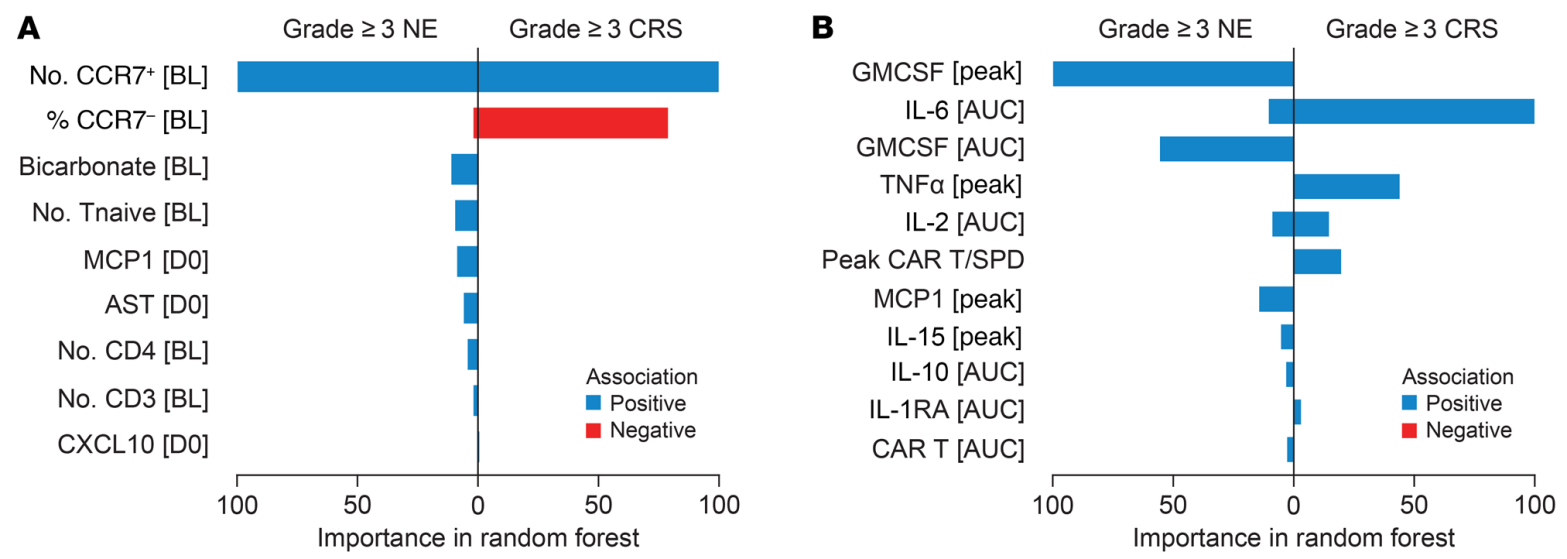
Multivariate analysis of covariates associated with high-grade toxicity identified product memory phenotypes and inflammatory cytokines. (**A**) Variable importance of preinfusion covariates associated with grade ≥ 3 CRS (*n* = 8) and grade ≥ 3 neurologic events (*n* = 19). (**B**) Variable importance of postinfusion covariates associated with grade ≥ 3 CRS (*n* = 8) and grade ≥ 3 neurologic events (*n* = 19). AST, aspartate aminotransferase; CAR, chimeric antigen receptor; CRS, cytokine release syndrome; MCP, monocyte chemoattractant protein; NE, neurologic events; Tnaive, naive T cells; SPD, Sum of product diameters.

Among pretreatment factors, multivariate analysis showed association of total number of infused CCR7^+^ T cells (naive and central memory T cell phenotype) with severity of grade ≥ 3 NE and CRS, while the percentage of effector and effector memory T cell phenotypes (CCR7^–^) exhibited a negative association with grade ≥ 3 CRS (h 8A and Supplemental Figure 12B). Furthermore, CCL2 (monocyte attractant protein 1/MCP1), AST, and CXCL10 on day 0, total number of infused naive T cells, CD3^+^ T cells, CD4^+^ T cells, and baseline bicarbonate levels were found to be associated with grade ≥3 NE (Figure 8A and Supplemental Figure 12B). Serum bicarbonate may contribute to cognitive dysfunction (19), possibly mediated by alterations in acid-base homeostasis, systemically and in cerebrospinal fluid (CSF). Notably, percentage of product T-naive cells did not associate with grade ≥ 3 NE and grade ≥ 3 CRS (Supplemental Figure 12C), which is consistent with observations in r/r LBCL treated with axi-cel (20, 21).

Among postinfusion analytes, GMCSF peak/AUC levels in serum emerged as the foremost analyte linked to grade ≥ 3 NE, followed by IL-6 AUC, IL-2 AUC, CCL2 peak, IL-15 peak, IL-10 AUC, IL-1RA AUC and CAR T cell expansion (Figure 8B and Supplemental Figure 12C). Peak levels of IL-15, found elevated in patients with grade ≥ 3 NE was also reported in LBCL (22). On the other hand, IL-6 AUC and TNF-α peak levels in serum were identified as the primary analytes associated with grade ≥ 3 CRS (Figure 8B). Strong associations with grade ≥ 3 CRS were further observed with peak CAR T cell levels normalized to tumor burden (SPD), IL-6 peak, IL-2 AUC, and IL-1RA AUC (Figure 8B). When the pretreatment and postinfusion variables were jointly analyzed, postinfusion immune-modulatory serum analytes such as GM-CSF, IL-15, IL-6, IL-2, TNF-α, and MCP-1 ranked higher compared with pretreatment product phenotype covariates for association with high-grade CRS and/or NE (Supplemental Table 4).

Univariate analyses corroborated the above observations providing additional insights (Supplemental Figure 12, B and D, and Supplemental 13, A and B). No association was found between grade ≥ 3 NE and tumor burden (data not shown). Interestingly, serum TNF-α, which associated with disease progression (Figure 2A), was elevated in patients with both grade ≥ 3 CRS and NE (Supplemental Figure 13C), making it a promising target to improve the therapeutic index of CAR T cells. Altogether, the above findings present an association of immune-modulatory cytokines and chemokines, and CAR-T cell expansion with high-grade CRS and/or NE in patients with r/r FL treated with axi-cel.

## Discussion

In ZUMA-5, axi-cel demonstrated a remarkable outcome for patients with r/r FL, with a median PFS of 57.3 months and a median overall survival not yet at 60-month data cutoff (23). Notably, durable responses were observed across patient subgroups, including patients with high-risk disease, such as POD24, high tumor burden, and high FLIPI score. Further, FL patients who relapsed and underwent retreatment with axi-cel (*n* = 13) demonstrated promising clinical efficacy posttretreatment, with 69% achieving CR (3).

Identifying biomarkers associated with efficacy and safety readouts in patients with r/r FL who received CAR-T cell therapy could provide insights into mechanisms of resistance and toxicity, hence inform patient management, development of next-generation products, and strategies with improved therapeutic index. There remains a scarcity of correlative studies exploring biomarkers associated with outcome (3, 4) in FL. In this study, we leveraged the largest available CAR T–treated r/r FL clinical dataset yet from the ZUMA-5 study (*n* = 124) and employed both univariate and multivariate analyses to investigate the association of many translational and clinical parameters with efficacy and high-grade CRS or NE, following treatment with axi-cel.

We observed that pretreatment levels of TNF-α and other inflammatory markers, including IL-12p40, IL2-Rα, ferritin, and IL-16 were associated with disease progression. This is consistent with previous observations, where elevated TNF-α was linked to increased tumor burden and poorer outcome in patients with r/r FL (5, 24). TNF-α is reflective of systemic inflammation, which adversely impact outcome with CAR T cells as observed in other studies (25). Further, TNF-α is known to play a role in pathogenesis of non-Hodgkin lymphoma (26) through upregulation of inflammatory and antiapoptotic signals (27).

We observed that baseline TMTV held greater prognostic value than SPD in patients with r/r FL, aligning with similar observations from ZUMA-7 study (28). TMTV threshold of ≥ 240 mL at baseline associated with disease progression in patients with r/r FL treated with tisagenlecleucel in ELARA study (29), while, in ZUMA-5, the threshold for significance for TMTV was 403.71 mL. The difference in significant TMTV thresholds between ZUMA5 versus ELARA studies may be explained by differences in baseline tumor burden, which was higher in ZUMA-5, compared with the ELARA study (5), and/or by different CAR T cell products used in the 2 studies, where the threshold of tumor burden above which an impact to PFS is observed could differ between axi-cel and tisa-cel treatments.

Pretreatment inflammatory cytokines and naive T cells in the axi-cel product showed a stronger association with clinical response than TMTV and CAR T cell expression. In r/r LBCL, the phenotype of CAR T cell product consistently associated with objective and ongoing response rate, as well as with time-to-events metrics such as EFS. Conversely, CAR T cell expansion associated with objective response but did not necessarily associate with ongoing response or time-to-event readouts (20, 30–33). This suggests that CAR T cell expansion is crucial for triggering a response, but the depth and durability of such response relies on additional factors, including product phenotype. It further indicates that a product enriched in T-naive phenotype provides its benefits not solely by impacting CAR T cell expansion. Possibly, a less differentiated product might be more resistant to bulkier and more immune-suppressive TME. Consistent with this notion, we observed that the T-naive phenotype associated with improved outcome only in FL patients with elevated TMTV.

Postinfusion covariates, including serum GM-CSF, IL-6, TNF-α, and CAR T cell expansion, ranked higher than pretreatment covariates, such as product T cell phenotype percentage and number of infused CAR T cells, for association with severe CRS and NE. Hence, early postinfusion inflammatory analytes coupled with CAR T cell expansion could potentially be monitored for anticipation of safety events. The contribution of product T-naive and central memory phenotype to severe CRS or NE appeared modest, driven by the total number of cells infused, which leads to higher CAR-T cell expansion. Our findings, together with supporting evidence from the literature (34, 35), indicate that enrichment of product-naive T cell phenotype could be coupled with inhibition of inflammatory mediators such as TNF-α (36, 37), to improve outcomes in patients with r/r FL patients.

In this study, TME composition in pretreatment tumor biopsies collected from a subset of patients with r/r FL was assessed by measuring gene expression via Nanostring IO360 and bulk RNA-seq. While B cell signature and CD19 expression associated with improved survival in r/r LBCL treated with axi-cel (15), the same was not observed in r/r FL tumors. Possibly, the B cell signature was not representative of limiting factors in the present study. In fact, r/r FL tumors had much higher levels of B cell signature compared with r/r LBCL tumors.

All but 1 of the evaluable ZUMA-5 patients (1 of 11, 9%) presented CD19 expression by IHC at disease progression. While we cannot exclude that some patients with FL would experience tumor antigen loss following CD19-directed CAR T cell therapy, the data are overall not supportive of antigen loss as a major mechanism of relapse in FL. The lack of CD19 detection by IHC in 1 patient could have been sporadic and not related to selective pressure from the CD19-targeting CAR-T cell therapy. Conversely, in aggressive 3L LBCL tumors treated with axi-cel (ZUMA-1), CD19-negative relapses were reported in up to 30% of patients (38, 39).

In r/r FL tumors, we observed that disease progression is associated with tumor inflammation characterized by IFN signaling and T cell checkpoint proteins. Tumor IFN signaling was associated with decreased axi-cel expansion in LBCL in a prior study, giving rise to expression of immune checkpoint ligands and causing immune dysregulation (17). The impact of type I and type II IFN on antitumor immunity is contingent upon the nature of induction, acute or chronic, and the cell type involved. Acute exposure to IFN signaling enhances antitumor immunity, whereas chronic exposure contributes to an immunosuppressive TME (40, 41). Consistent with our results, LAG3^+^ exhausted T cells associated with shorter PFS in the ELARA trial (29). RNA-seq and GSEA further support this, showing enrichment of Hallmark IFN-α/γ response pathways in progressive disease, linked to T cell exhaustion and a dysfunctional TME. “G2M checkpoint”, “E2F targets” and “mTORC1 signaling” pathways also correlated with disease progression and might represent immune exclusionary and metabolically restrictive TME features. In solid tumors and Hodgkin lymphoma, chronic IFN signaling leading to T cell exhaustion has been implicated in immune evasion and resistance to immunotherapy. Promisingly, the use of JAK/STAT inhibitors that block IFN signaling has shown therapeutic benefit by reversing this T cell suppression (42, 43). Our findings suggest that a similar phenomenon may occur in follicular lymphoma, where the slow-growing tumors and persistent IFN stimulation could deeply exhaust T cells, preventing optimal activity of CAR T cells.

Previous studies have highlighted the association of TMTV and FLIPI score with durable response to therapy (44, 45). While FLIPI score serves as the primary method for risk stratification in clinical practice, our study demonstrated that combining FLIPI score with pretreatment serum TNF-α and/or baseline TMTV enhanced the risk stratification of patients with FL. This study, however, has certain limitations. The analyses herein are exploratory and retrospective. Conclusions drawn from these data will require confirmation in an independent validation cohort.

**Figure 9:**
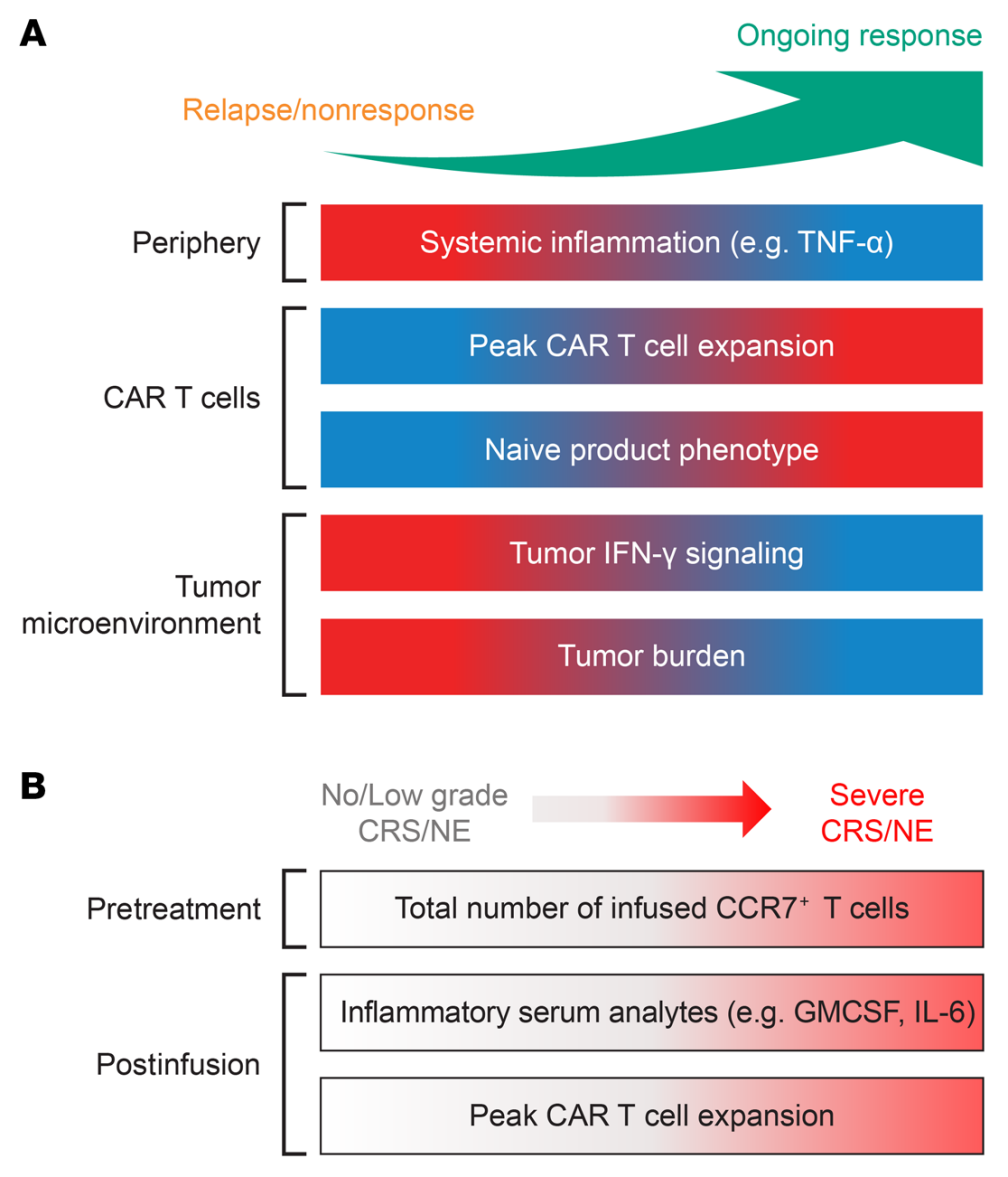
Overview of significant associations with clinical response in FL. (**A**) Biological markers linked to durable response in patients with r/r FL treated with axi-cel. (**B**) Biological markers linked to high-grade CRS and NE. Red represents higher levels and blue represents lower levels in panel **A**. Red represents severe CRS/NE in panel **B**. CAR, chimeric antigen receptor; FL, follicular lymphoma.

Overall, multivariate analyses showed efficacy to be predominantly associated with preinfusion covariates (from baseline and day 0), and high-grade CRS and NE to be strongly associated with postinfusion covariates. Our findings underscore the significance of pretreatment systemic inflammation (e.g. serum TNF-α levels), T-naive product phenotype, TMTV, tumor IFN signaling, and posttreatment CAR T cell expansion as independent prognostic factors, positively or negatively (covariate specific) associated with efficacy, in patients with r/r FL treated with axi-cel (Figure 9A). Postinfusion CAR T cell expansion and secretion of inflammatory cytokines and chemokines, along with the number of infused early memory CCR7^+^ T cells in product emerged as determinants of high-grade (grade ≥ 3) CRS and neurologic events (Figure 9B). These findings could inform risk stratification of patients, development of next generation products, and combination strategies to improve the therapeutic index of CAR T cell therapy in FL.

## Methods

### Sex as a biological variable.

Female and male participants were included in all univariate and multivariate analysis of this study.

### Patient samples.

Evaluable samples from patients in the safety analysis set of ZUMA-5 (NCT03105336), consisting of 124 patients with FL who were treated with axi-cel on study, were analyzed. Both male and female patients were enrolled in ZUMA-5 and were included in this analysis. Sex was considered as a biological variable in the multivariate analysis as a covariate (it was not found to be associated with outcome).

Efficacy and safety endpoints in patients with FL from the 36-month data analysis were used in this investigation (3). Patients who were in ongoing response at least 3 years after axi-cel infusion were said to achieve durable response, whereas those patients who had a complete or partial response and subsequently experienced disease progression were termed as relapsed. Nonresponders were those who achieved stable disease as their best response. A subset of eligible patients received retreatment, per protocol (4). For this investigation, tumor biopsies were obtained from patients prior to treatment (at screening/diagnosis or at baseline/on study before lymphodepleting chemotherapy and axi-cel infusion), after axi-cel infusion between days 7 and 14, or later, at relapse. Herein, baseline is defined as the most recent readout prior to initiation of LD. Baseline includes leukapheresis samples for all participants. A total of 121 pretreatment, 12 posttreatment, and 18 postrelapsed formalin-fixed paraffin embedded (FFPE) biopsies were analyzed. Of the 18 patients with relapse, 11 had paired biopsies at pretreatment and postrelapse that were tested using IHC. Further, 34 pretreatment, were analyzed using NanoString PanCancer IO360 panel and 35 pretreatment biopsies were further evaluated for validation of results using RNA-seq. Thirty samples were common between nanostring and RNA-seq analysis.

### Quantification of CAR T cells.

CAR T cells were quantified using TaqMan quantitative PCR (Thermo Fisher Scientific), as described previously (46–49). CAR+ cells were reported as cells per microliter blood, which was calculated by normalizing CAR gene expression to actin expression in peripheral blood mononuclear cells.

### Measurement of serum cytokines.

Longitudinal patient serum cytokines were analyzed using internally qualified multiparametric assays (50). IL-1RA, IL-2Rα, Ferritin, and PDL1 serum levels were measured by Simple Plex ELLA (SimpleProtein), and granzyme A, granzyme B, sFASL, and perforin were measured by Luminex (EMD Millipore). IL1B and all other serum analytes were measured by V-Plex Multiplex assay panels (Meso Scale Discovery).

### Flow cytometry analysis.

Samples were processed, stained, and analyzed as previously described for end-of-production CAR T cell flow cytometric characterization (50). The antibodies tested were CD3-FITC (clone UCHT1- BioLegend #300406), CD8-APC-H7 (clone SK1- BD #560179), CCR7-BV650 (clone G043H7- BioLegend #353233), and CD45RA-APC (clone HI100- BioLegend #304112). Flow cytometry was performed using a FACSCanto II (BD Biosciences). Data analysis was performed using FlowJo Software v10 (FLOWJO, LLC) with standardized gating and compensation strategies.

### IHC.

IHC staining was performed using tissue sections and an automated immunostainer (DAKO). H&E staining allowed preliminary formalin-fixed paraffin-embedded tissue evaluation for block quality controls. Slides were scanned with the Nanozoomer XR to generate digital images (20 ×). A pathologist identified the tumor area and provided qualitative and semiquantitative assessments. IHC staining for CD-19 (LE-CD19, surface domain) and CD-20 (L26, cytoplasmic domain) was scored by a composite H-score. IHC staining with H-scores of 0–4 was assigned as “negative”, while 5–300 was assigned as positive for the purpose of data quantification.

### NanoString IO360 and RNA-seq analysis of baseline tumor biopsies.

Transcriptomic analysis of ZUMA5 patient tumor biopsies was performed using the PanCancer IO360 gene expression panel (*n* = 34) as described previously (14), and bulk RNA-seq (*n* = 35). Pretreatment biopsies from 30 out of 35 participants were analyzed by both Nanostring and RNA-seq. In the tumor biopsies where more than 50% of the tissue was represented by nontumor tissue (per pathologist examination), macrodissection was performed to focus the mRNA extraction and subsequent gene expression analysis on the tumor area. Wet laboratory testing of Nanostring IO360 was performed at Neogenomics, while bulk RNA-seq was performed in-house. ZUMA7 Nanostring IO360 testing and analyses were performed as previously described (15).

Raw RCC files of Nanostring data were uploaded on ROSALIND (ROSALIND Inc.) (51). Comparison results between the ongoing versus others group was downloaded from the software for further analysis. A predefined set of 21 genes obtained from the PanCancer IO360 Panel using a proprietary algorithm from Veracyte were comprehensively scored as ImmunoSign 21 (IS21). Cell subtypes within the tumor microenvironment and their association with clinical outcomes were investigated using the IS21 and IO360 scores (Supplemental Table 5).

Illumina Stranded Total RNA Prep, Ligation with Ribo-Zero Plus kit (Illumina #20040529) was used to prepare RNA-seq libraries for next-generation sequencing of pretreatment biopsies. Briefly, total RNA was subjected to rRNA depletion followed by fragmentation, cDNA synthesis, ‘A’ tailing, anchor ligation, and, finally, amplification of the anchor ligated DNA fragments. Final bar-coded libraries were quantified using Qubit and quality was checked on the Tape Station. Libraries were then pooled in equimolar concentration and sequenced on NovaSeq 6000 with a paired-end read length of 55 bp.

For RNA-seq data processing raw FASTQ files were trimmed, filtered, and quality controlled with fastp version 0.20.1 (52). Reads were aligned with GENCODE Human release 38 (GRCh38.p13) using STAR version 2.7.8 (53). The median number of aligned reads was 91 million (95% CI 82–97 million). The bam files were assembled into potential transcripts using StringTie version 2.2.1 (54). Assembled transcripts were quantified using the R package ballgown version 2.30.0.

Differentially expressed genes (DEGs) in ongoing versus other tumor samples were identified using DESeq2 (*P <* 0.05). These DEGs were visualized through a heatmap and a Volcano plot, which illustrated the degree of differential expression according to –Log_10_(*P* value). Enrichment analysis of pathways was done using the *fgsea* (version 1.26.0) for GSEA on genes ranked by log_2_ fold-change (55). The landmark analysis was performed using predefined Nanostring signatures to limit data overfitting. RNA-seq was used for confirmatory purposes, differential enrichment scores (12), and pathway enrichment analysis.

To estimate the enrichment of specific immune traits and immune cell types, gene expression deconvolution was performed using single-sample GSEA (ssGSEA). This analysis was conducted with the GSVA package (56), utilizing cell-specific gene signatures detailed in Supporting Information Supplemental Table 6. Specifically, the analysis included 34 immune trait signatures (12), 4 immune exhaustion signatures (57), the 14 cell-specific immune signatures from Bindea et al. (58), and IFNG-RS and IFNG-GS (10). Enrichment scores (ES) were calculated on log_2_-transformed data. Simultaneously, ssGSEA was used to analyze shifts in tumor-infiltrating immune cell subsets. The differentially enriched scores for each immune trait or cell subset were visualized using a Volcano plot (illustrating the degree of differential expression according to –Log_10_(*P* value)) and a heatmap showing genes common to both RNA-seq and Nanostring analyses. To evaluate differences in immune cell presence between ongoing and other response conditions, enrichment scores were compared using the limma test.

### Survival analysis.

Patients were divided into 2 groups based on the median gene expression or enrichment scores of each signature. Progression-free survival (PFS) was summarized with Kaplan-Meier curves using a modified version of the ggkm function (59) or ggsurvplot function in survminer package. Survival data were censored after a follow-up period of 5 years. Hazard ratios (HR) between groups (high: >median and low: ≤median), corresponding *P* values, and CIs were calculated using Cox proportional hazard regression with the R package survival (version 4.2.1).

For each variable, the proportional hazard assumption (PHA) was checked by computing the Pearson product-moment correlation (r) between the Schoenfeld residuals and the transformed (log) survival time using the cox.zph R function (60). Cox proportional hazard models were stratified for variables with a significant violation of the PHA.

### Multivariate analysis.

Associations of clinical characteristics and translational biomarkers with efficacy and safety outcomes were examined using multivariable analysis. Covariates with a high percentage (> 80%) missing or high co-linearity/correlation (R ≥ 0.9 or R ≤ –0.9) were first excluded in a preprocessing step. Missing values were imputed using MissForest algorithm (61). During colinearity exclusion, we arbitrarily chose AUC over peak for inclusion into the multivariate analysis. Some other considerations included (a) several serum analytes (e.g. cytokines) had 0 variances at pretreatment timepoints (screening and day 0) because the levels of these analytes before infusion of CAR T cells were below the lower limit of detection (LLOQ); and (b) HGB and HCT were correlated with each other (R = 0.96) and we have kept HGB for the multivariate analysis.

For time-to-event outcomes, covariates were screened using univariate log-rank test based on their median and optimal cutoffs, then further selected by permutation-based independence test in a conditional inference tree (62). Optimal cutoffs were selected as the ones that provide the best separation of the responses into 2 groups while requiring at least 30% of patients in each group. The conditional random survival forests model was applied to the covariates in a multivariable setting (63, 64). Binary outcomes were analyzed using a similar procedure. Wilcoxon test, log-rank or χ^2^ test were used to compare all pretreatment and postinfusion covariates (univariate screening) by ongoing response, PFS, high-grade CRS, and neurologic events. Four algorithms were used in feature selection, including weight of evidence and information value, penalized logistic regression, conditional random forest, and XGBoost (65). Covariates that were selected by at least 2 of these methods were included in the final random forests model to generate variable importance. The permutation-based conditional variable importance from all the outcomes were reported to help evaluate the importance with the presence of correlated covariates (66). The average variable importance of 50 random forests repetitions using different random seeds was presented to minimize uncertainty. Covariates ranked in the top 10 were considered as top-ranking covariates.

In total, 382 covariates were considered for this analysis. Eighty-six covariates were excluded in the data pre-processing step. Missing imputation was carried out among the remaining 296 covariates (230 pretreatment and 66 postinfusion). Postinfusion corticosteroid and tocilizumab usage were excluded in the analysis of toxicity outcomes. Results for efficacy and safety outcomes were recorded as reported in Supplemental Table 2 (multivariate analysis for joint pretreatment and postinfusion covariates), Supplemental Table 3 (multivariate analysis for pretreatment covariates), Supplemental Table 4 (multivariate analysis for postinfusion covariates).

### Statistics.

Covariates and biomarkers from secondary and exploratory endpoints were investigated for associations with efficacy, safety, and CAR T cell expansion. For univariate analysis, Spearman’s rank-order correlation was used to evaluate the association between analytes. Logistic regression was used to evaluate the relationship between a covariate and an outcome. Wilcoxon rank sum and Kruskal-Wallis tests were used to compare covariates between ongoing responders versus relapsed and nonresponders, tumors with high versus low *IFNG* IO360 score, and low versus high grades of toxicity for assessment of *P* values. For these post hoc analyses, all *P* values were descriptive and *P <* 0.05 was considered significant. No adjustments for multiplicity testing were performed. Covariates were subdivided into subgroups by median value, quartile values, or as indicated (e.g., SPD value of 3721 mm^2^). Plots were generated using TIBCO Spotfire, SAS, R, or GraphPad Prism.

### Study approval.

An IRB approved the study at each study site (Western IRB, Puyallup, Washington, USA; Advarra IRB, Columbia, Maryland, USA; UCLA Office of Human Research Protection Program, Los Angeles, California, USA; IntegReview Ethical Review Board, Austin, Texas, USA; Dana Farber Cancer Institute, Boston, Massachusetts, USA; University of Southern California Health Sciences Campus, Los Angeles, California, USA; Fred Hutchinson Cancer Research Center, Seattle, Washington, USA; MedStar Health Research Institute–Georgetown University, Washington, DC, USA; The University of Texas MD Anderson Cancer Center IRB, Houston, Texas, USA; Vanderbilt University IRB, Nashville, Tennessee, USA; Columbia University Medical Center IRB, New York, New York, USA; University of Miami IRB, Miami, Florida, USA; Comite de Protection des Personnes Ile-de France, Paris, France; University of Pittsburgh IRB, Pittsburgh, Pennsylvania, USA; The Ohio State University Office of Responsible Research Practices, Columbus, Ohio, USA) and the trial was conducted in accordance with the Good Clinical Practice guidelines of the International Conference on Harmonization.

### Data availability.

Kite is committed to sharing clinical trial data with external medical experts and scientific researchers in the interest of advancing public health. As such, Kite shares anonymized individual patient data (IPD) on request or as required by law and/or regulation. Qualified external researchers may request IPD for studies of Kite or Gilead compounds approved in the United States and the European Union with a marketing authorization date on or after 1 January 2014 and are publicly listed on clinicaltrials.gov or the European Union-Clinical Trials Register (EU CTR). The IPD will be available for request 6 months after US Food and Drug Administration and European Medicines Agency approval of the data. Such requests are at Kite’s discretion and are dependent on the nature of the request, the merit of the research proposed, availability of the data, and the intended use of the data. If Kite agrees to the release of clinical data for research purposes, the requestor will be required to sign a data sharing agreement to ensure protection of patient confidentiality before the release of any data.

Values for all data points in graphs are reported in the Supporting Data Values file.

## Author contributions

SP contributed to conceptualization, methodology, data acquisition and analysis, data interpretation, writing, and project administration. JY, GT, WZ, DR, and WP, contributed to the methodology and data analysis. JB contributed to the methodology, data acquisition, and analysis. SS contributed to data analysis. MD and RSS contributed to the methodology and data acquisition. SB, HM, and CAJ contributed to the clinical methodology. QS contributed to supervision of data analysis. MM contributed to conceptualization and methodology. SF contributed to the conceptualization, methodology, writing, data interpretation, data analysis supervision, and project supervision. DB contributed with methodology, writing, andata interpretation, and data analysis supervision. SSN contributed to the conceptualization and project supervision. All authors contributed to the drafting, review, and editing of the manuscript.

## Supplementary Material

Supplemental data

ICMJE disclosure forms

Supporting data values

## Version 1 06/19/2025

In-Press Preview

## Version 2 08/15/2025

Electronic publication
