# Supplementary material for: Clinical, tumor, and product features associated with outcomes after axicabtagene ciloleucel therapy in follicular lymphoma
Source: J Clin Invest. 2025 Aug 15;135(16):e181893. doi: 10.1172/JCI181893 (PMC12352890; doi:10.1172/JCI181893)
Supplement: Supplemental data [file jci-135-181893-s155.pdf]

**Clinical, tumor and product features associated with outcomes after axicabtagene ciloleucel therapy in follicular lymphoma**

**Poddar et al.**

**Supplemental Material**

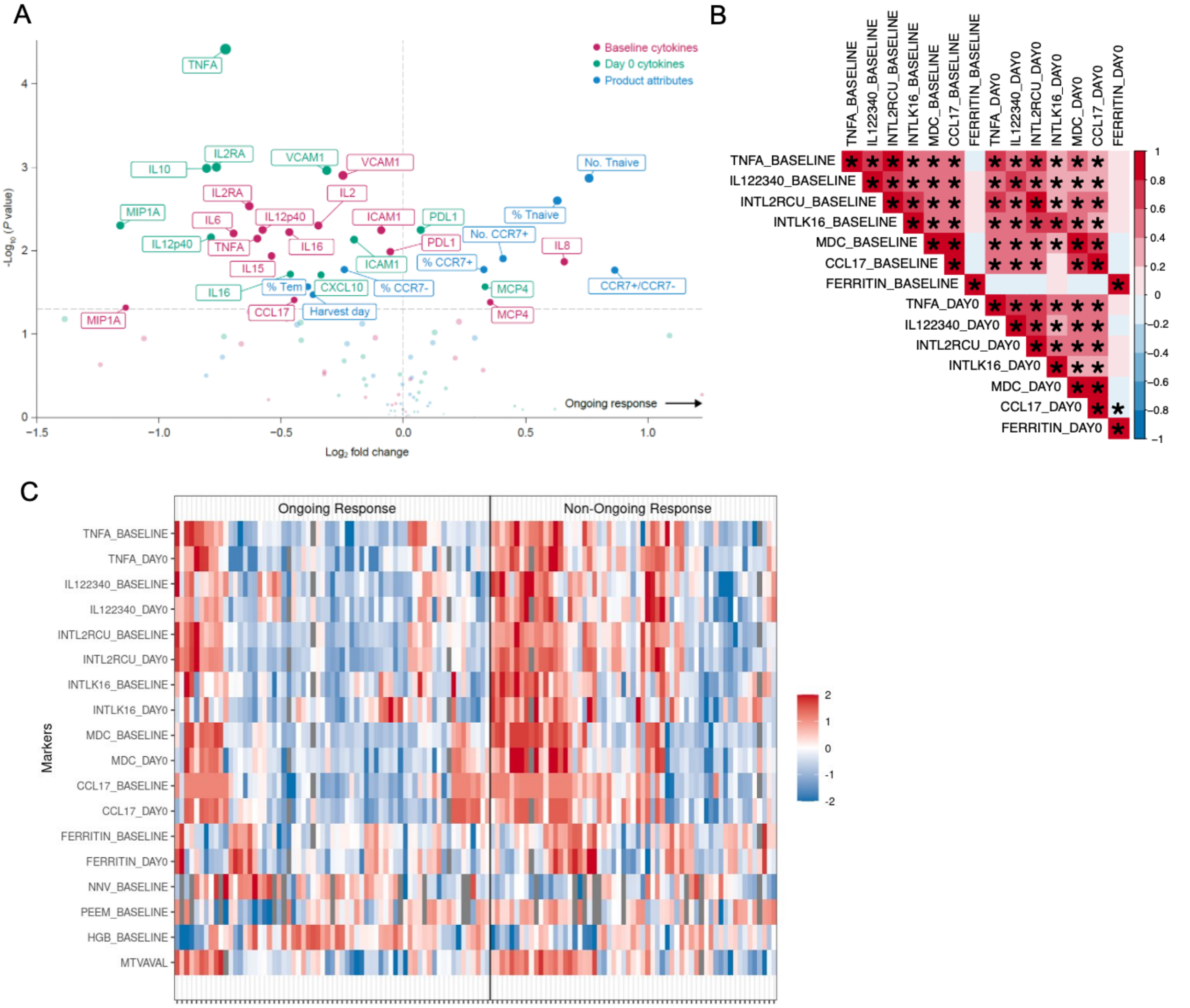


**Figure S1. Univariate analysis of covariates showing association with ongoing response and PFS**

(A) Volcano plot showing univariate associations of pre-treatment serum cytokines and product attributes with ongoing response; Wilcoxon rank sum test was used for assessment of p value, p<0.05 was considered significant. (B) Correlation plot showing cross-correlation between TNFa, IL12 p40, IL2Ra, IL-16, MDC, CCL17 and Ferritin at baseline and day 0. (C) Heatmap showing subject-level pre-treatment values of covariates identified by multivariate analysis between ongoing responders vs. relapsed and non-responders.

FLIPI, follicular lymphoma International prognostic index; HGB, hemoglobin; MDC, macrophage-derived chemokine; MIP, macrophage inflammatory protein; MCP, monocyte chemoattractant protein; PDL1, programmed death ligand 1; PFS, progression-free survival; TMTV, total metabolic tumor volume; Tnaive, naive T cells;TNF, Tumor necrosis factor; IL122340, Interleukin 12+23 p40; ILTL2RCU, Interleukin 2 receptor alpha; MDC, macrophage-derived chemokine; INTLK16, Interleukin 16; CCL17, C-C motif chemokine ligand 17

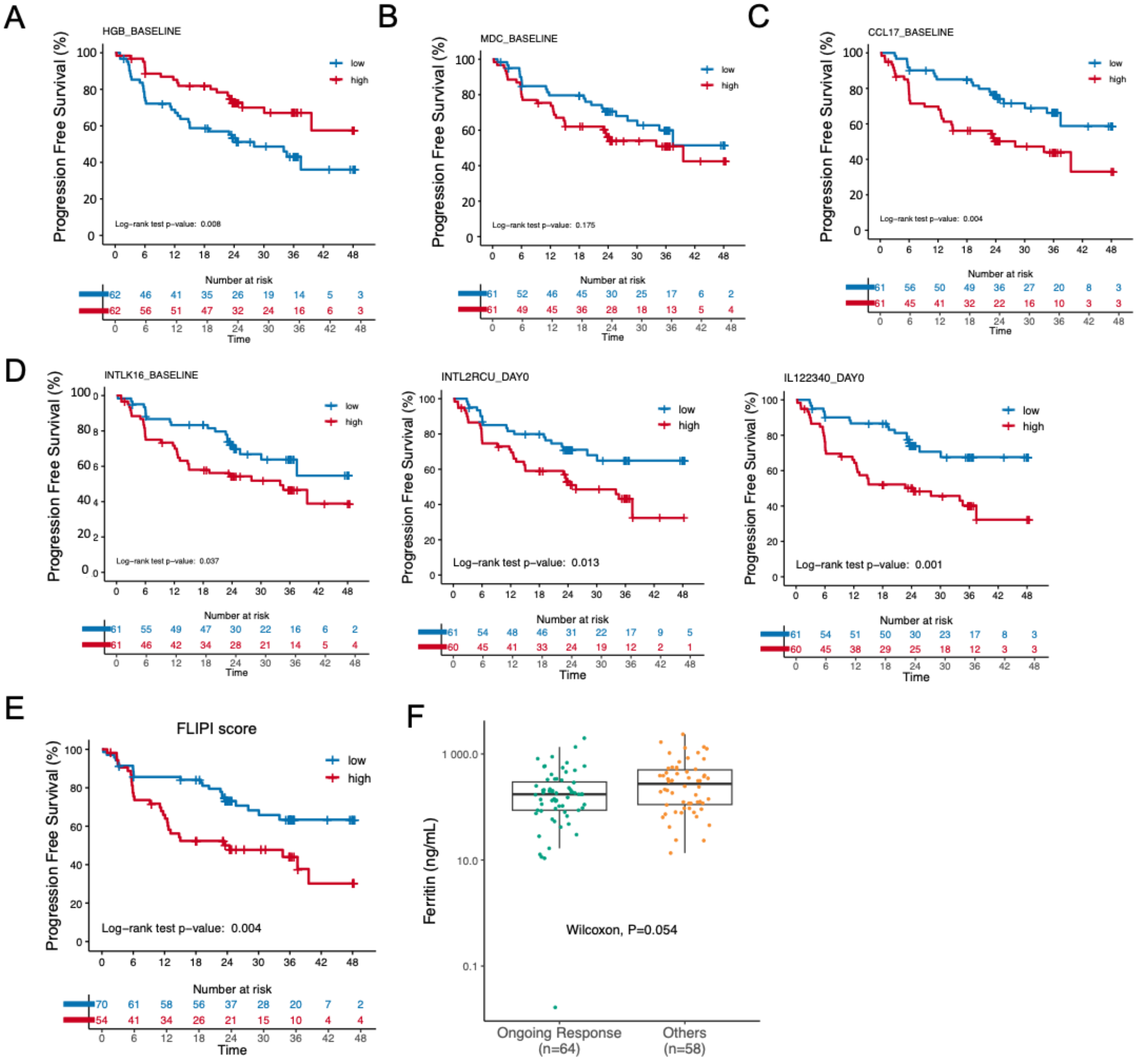


**Figure S2. KM-estimated PFS curves of pre-treatment covariates associated with response in multivariate analysis**

(A) KM estimated PFS by baseline Hemoglobin. (B) KM estimated PFS by baseline CCL22. (C) KM estimated PFS by baseline CCL17. (D) KM estimated PFS by immunomodulatory IL-16 (baseline) and inflammatory markers IL2Ra (day 0), IL-12 p40 (day 0). (E) KM estimated PFS by FLIPI score (>2 vs. ≤2). (F) Boxplot of inflammatory marker Ferritin by ongoing response. For panels A, B, C, and D, high vs. low is defined by above median and below median respectively. Log-rank test is performed to assess p value.

FLIPI, follicular lymphoma International prognostic index; HGB, hemoglobin; MDC, macrophage-derived chemokine; IL122340, Interleukin 12+23 p40; ILTL2RCU, Interleukin 2 receptor alpha; INTLK16, Interleukin 16; MTVAVAL, Total metabolic tumor volume.

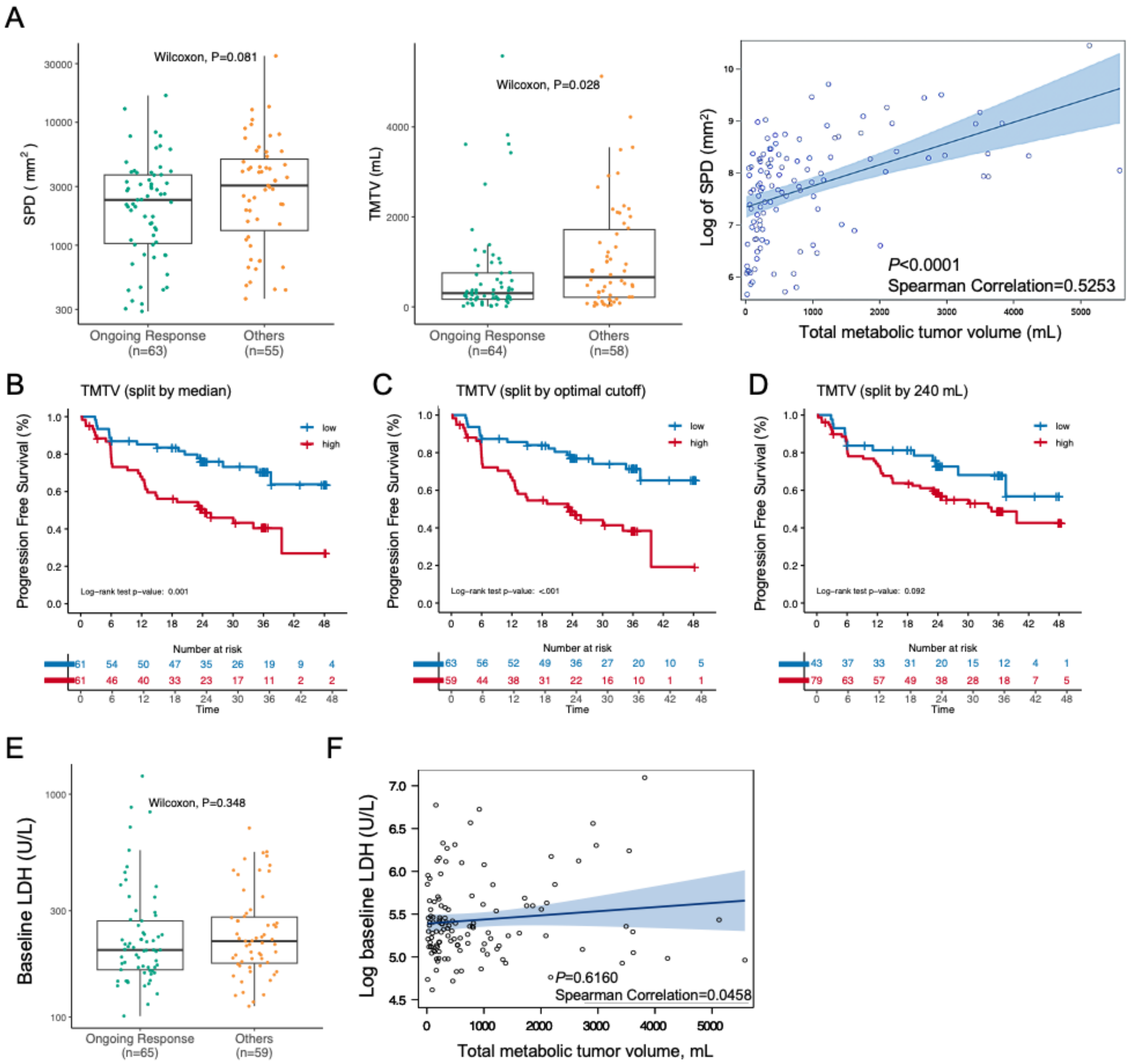


**Figure S3. Association of serum inflammatory markers at baseline with ongoing response and TMTV**
(A) (left) Box plots of baseline SPD and TMTV in patients with ongoing response vs. relapsed and non-response. (Right) Scatter plot showing spearman correlation (95% CI) of SPD and TMTV. (B) KM estimated PFS by TMTV, split by median (C) KM estimated PFS by TMTV, split by optimal cutoff. (D) KM estimated PFS by 240 mL TMTV. (E) Box plot of baseline LDH in patients with ongoing response vs. relapsed and non-response. (F) Spearman correlation (95% CI) between baseline LDH and TMTV.

LDH, lactate dehydrogenase; SPD, sum of product diameters; TMTV, total metabolic tumor volume.

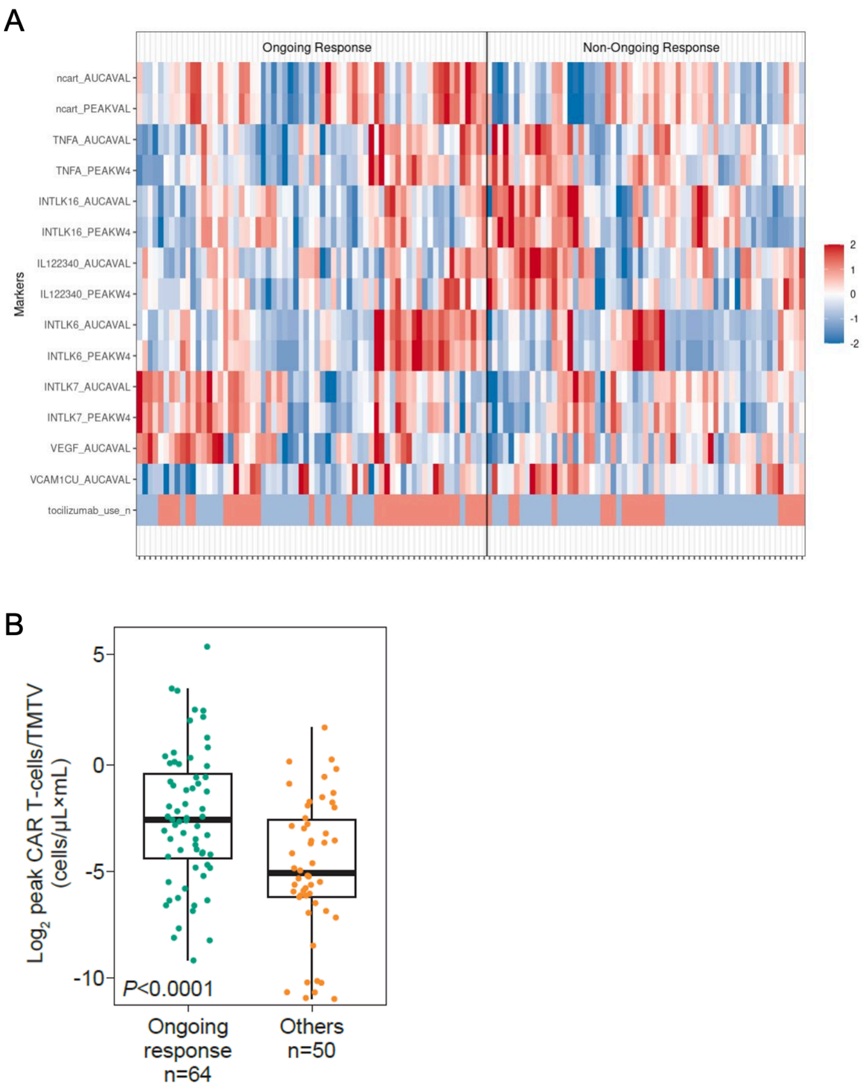


**Figure S4. Subject level information of post-infusion covariates associated with clinical response**

(A) Heatmap comparing post-infusion levels of covariates ranked by multivariate analysis between ongoing responders vs. relapsed and non-responders. (B) Boxplot showing CAR T cells peak/TMTV for ongoing response vs. others; Wilcoxon rank sum test was used to assess p value.

ncart, number of CAR T cells; VEGF, Vascular endothelial growth factor; TNF, Tumor necrosis factor; IL122340, Interleukin 12+23 p40; INTLK16, Interleukin 16; INTLK6, Interleukin 6; INTLK7, Interleukin 7; VEGF, Vascular endothelial growth factor; VCAM1CU, Vascular cell adhesion molecule 1; tocilizumab_use_n, Patients administered tocilizumab.

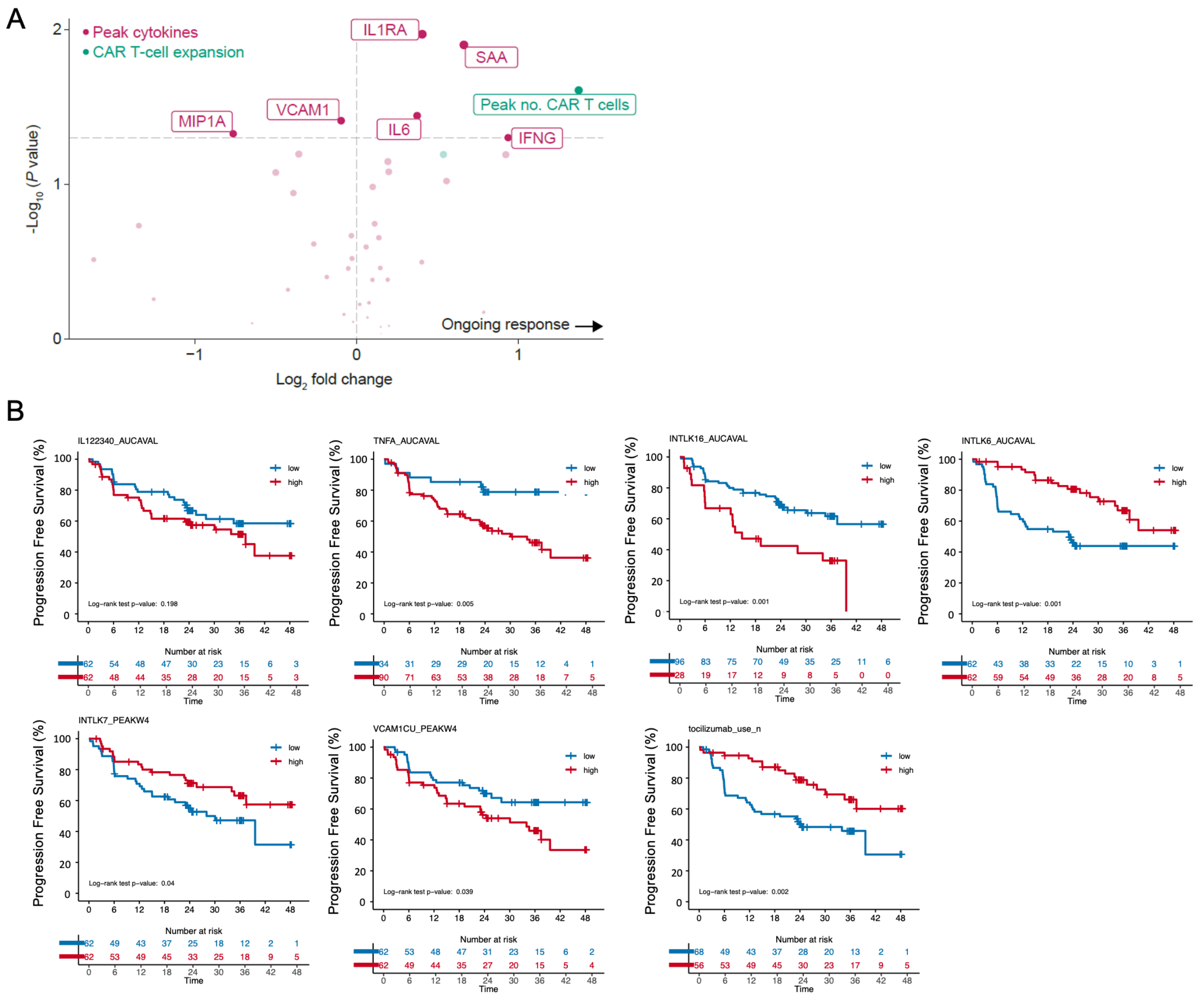


**Figure S5. Univariate analysis of post-infusion covariates associated with clinical response**

(A) Volcano plot showing univariate associations of post-infusion serum analytes and CAR T cell PK with ongoing response. (B) KM-estimated PFS by IL12p40 AUC, TNFα AUC, IL16 AUC, IL6 AUC, IL7 Peak, VCAM1 (above and below median) and Tocilizumab use (Y vs. N).

ncart, number of CAR T cells; VEGF, Vascular endothelial growth factor; TNF, Tumor necrosis factor; IL122340, Interleukin 12+23 p40; INTLK16, Interleukin 16; INTLK6, Interleukin 6; INTLK7, Interleukin 7; VEGF, Vascular endothelial growth factor; VCAM1CU, Vascular cell adhesion molecule 1; tocilizumab_use_n, Patients administered tocilizumab; PFS, progression-free survival.

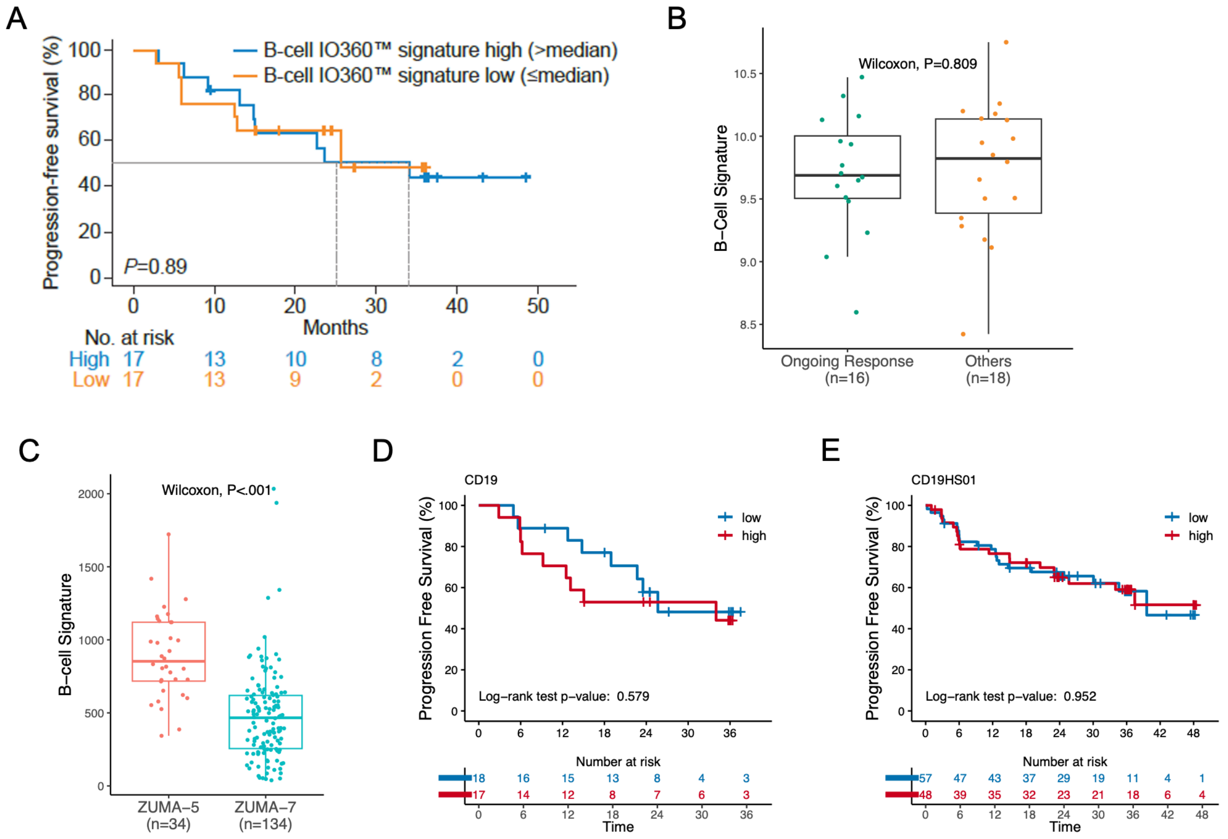


**Figure S6. B-cell signature does not associate with clinical response**

(A) KM-estimated PFS by median B-cell IO360™ signature score. (B) Boxplot of B-cell IO360 score by ongoing response in r/r FL patients. (C) Comparison of B-cell IO360 score between axi-cel treated r/r FL patients in ZUMA-5 (3L FL) and r/r LBCL patients in ZUMA-7 (2L LBCL) studies. (D) KM-estimated PFS by median CD19 gene expression in r/r FL patients. (E) KM-estimated PFS by median CD19 H-score in r/r FL patients.

KM, Kaplan-Meier; PFS, Progression-free survival; r/r, Relapsed/refractory; FL, Follicular lymphoma; LBCL, Large B-cell lymphoma.

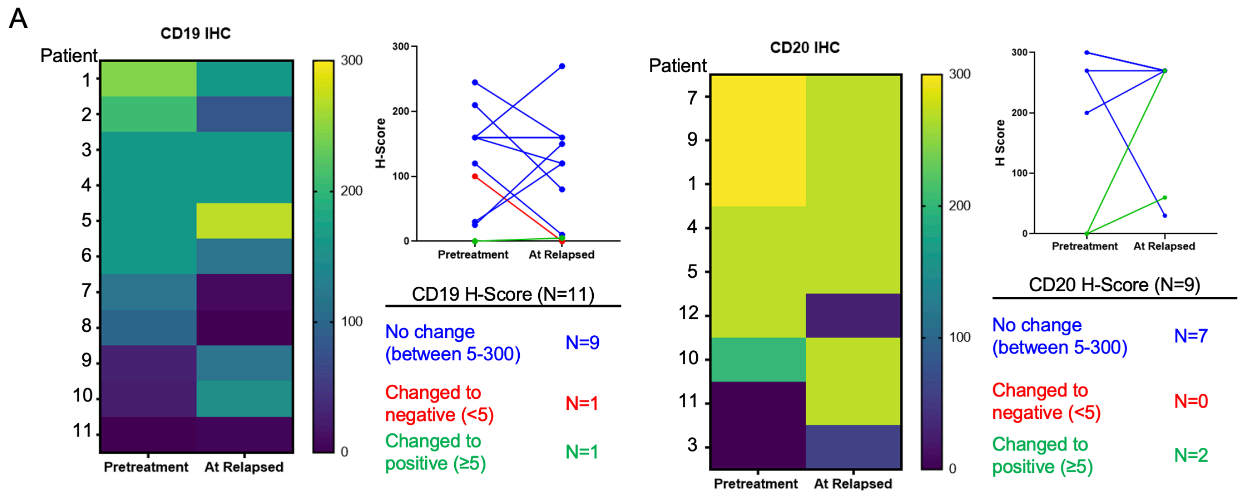


**Figure S7. CD19 loss is unlikely a resistance mechanism of r/r FL patients treated with axi-cel**
(A) Paired CD19 and CD20 expression in pre-treatment and progression biopsies in relapsed FL patients.

FL, follicular lymphoma; IHC, immunohistochemistry; PFS, Progression-free survival.

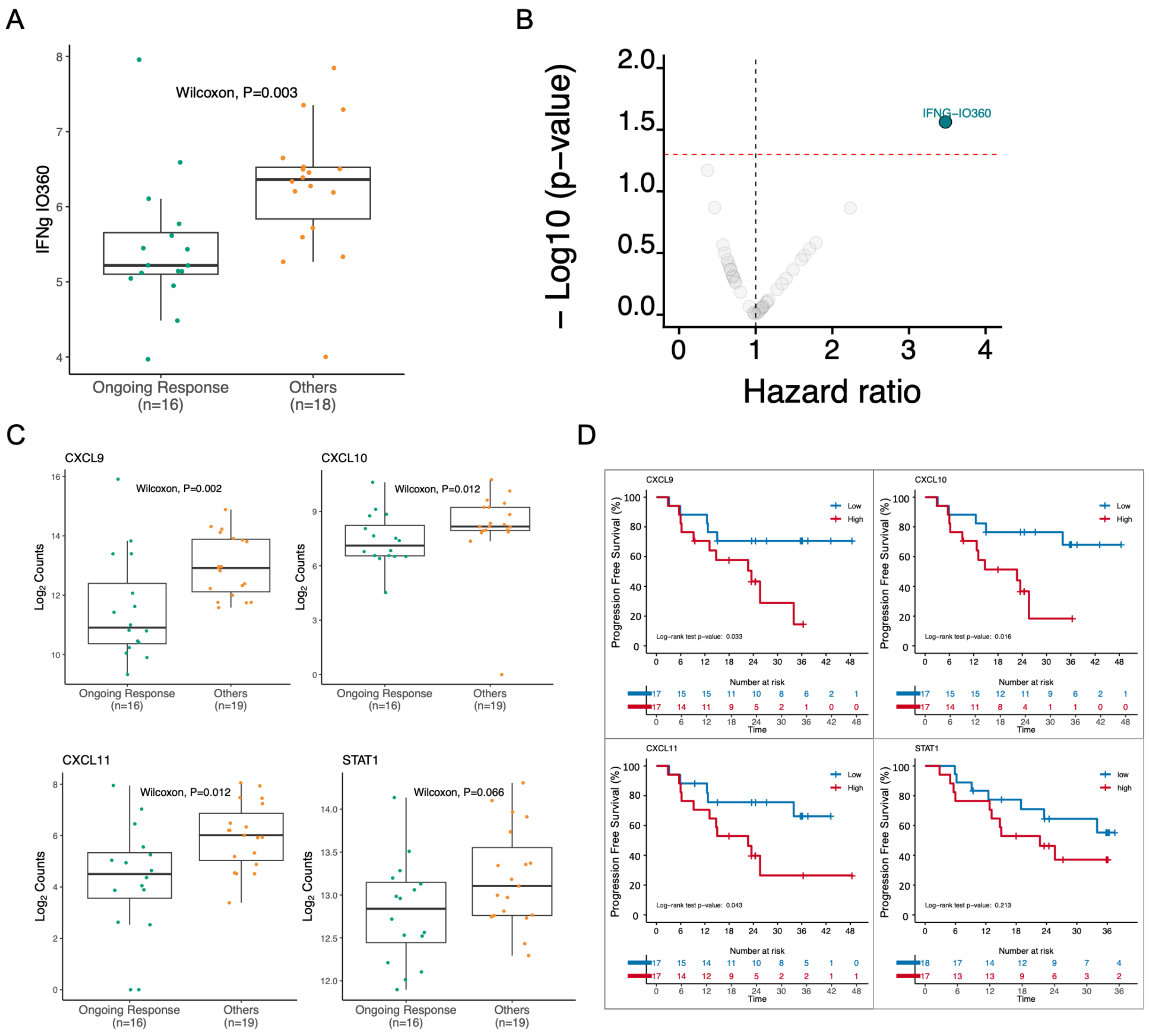


**Figure S8. Association of genes in IFNg IO360 signature with ongoing response and PFS.**
(A) Boxplot of IFNg IO360 score stratified by ongoing response status. (B) Volcano plot of IO360 gene expression signatures and associated Cox proportional hazards statistics. (C) Boxplots of CXCL9, CXCL10, CXCL11 and STAT1 gene expression stratified by ongoing response status. (D) KM-estimated PFS by median expression of CXCL9, CXCL10, CXCL11 and STAT1 genes.

KM, Kaplan-Meier; PFS, Progression-free survival.

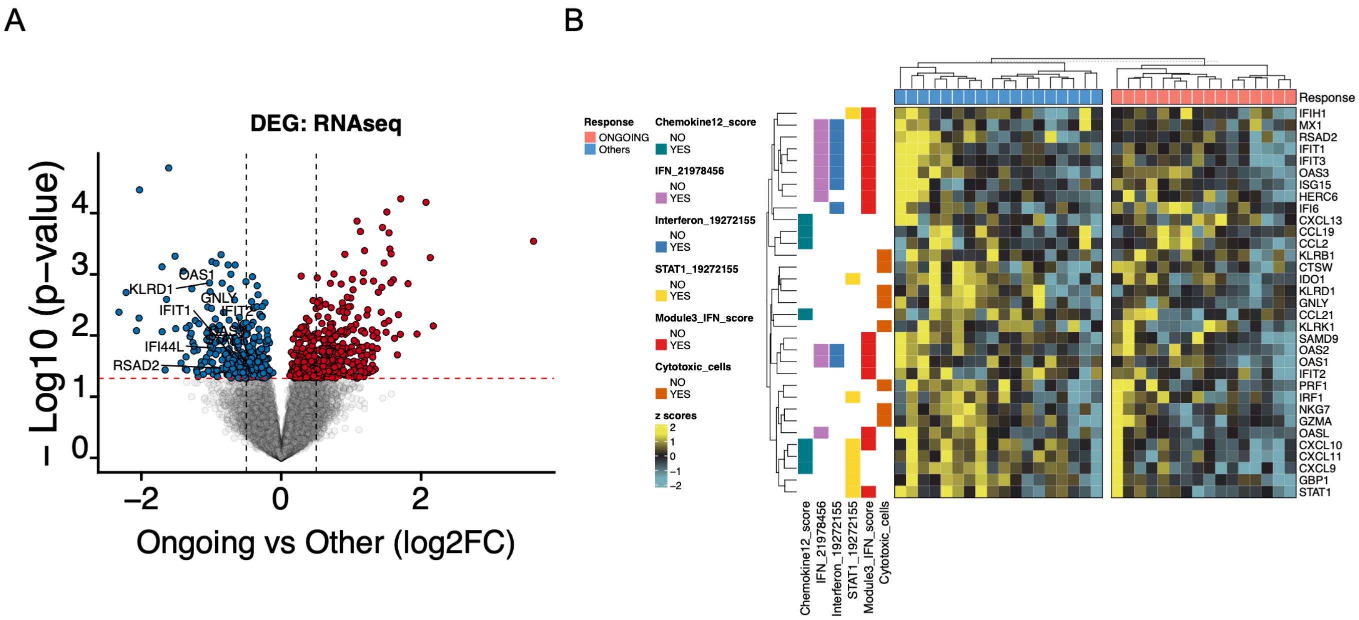


**Figure S9. Validation of Nanostring gene expression using RNAseq analysis.**
(A) Volcano plot showing differential gene expression of RNAseq dataset by ongoing response. (B) Heatmap showing expression of IFN signaling genes that are included in the presented signatures in Nanostring dataset by ongoing response status.

ISG, Interferon stimulating genes; DEG, differentially expressed genes; FC, fold change.

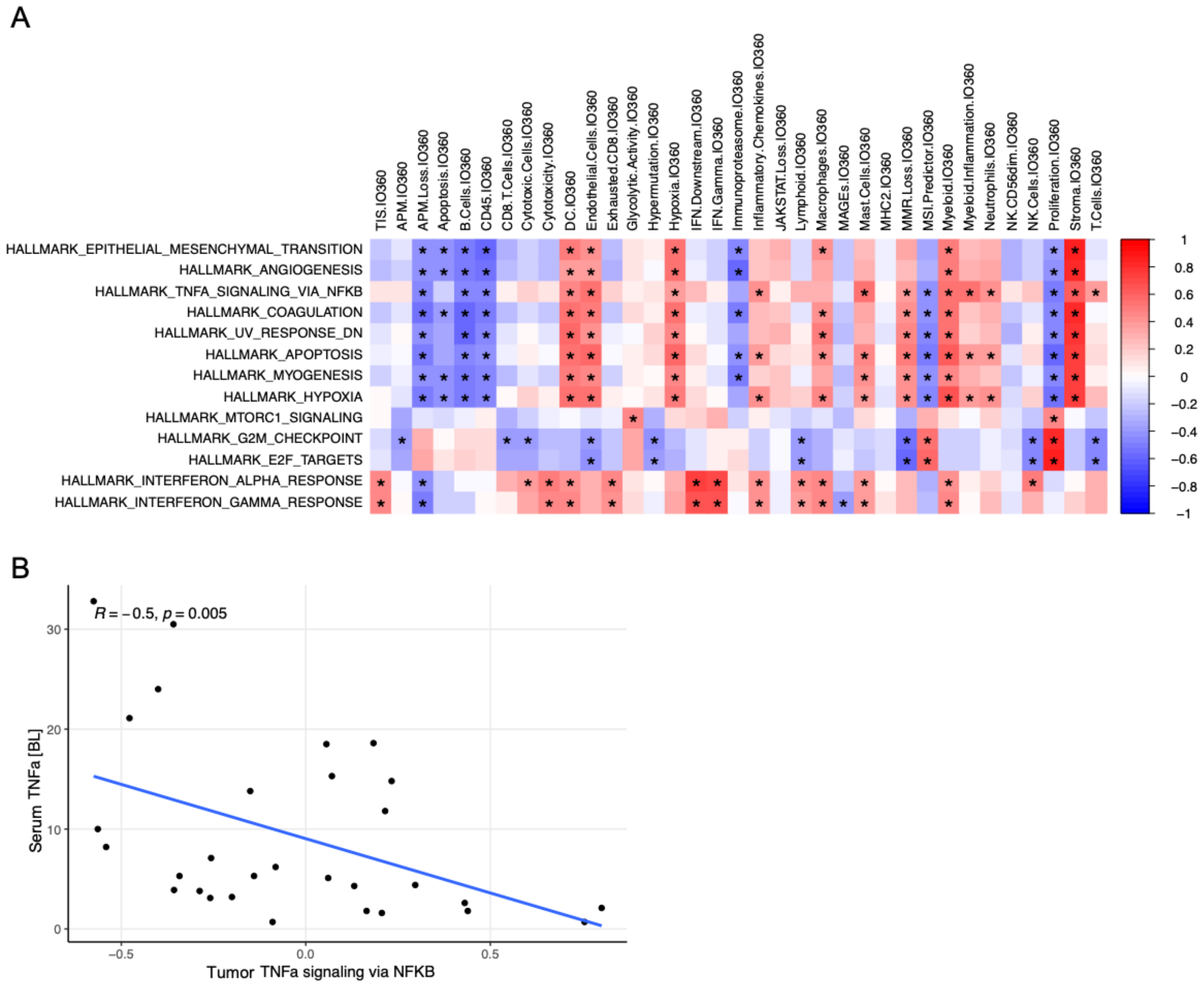


**Figure S10. Correlation of GSEA hallmark pathways and TNF inducible genes with IO360 scores and serum TNFα respectively**

(A) Heatmap showing correlation between GSVA score of significant hallmark pathways and Nanostring IO360 scores (B) Correlation of hallmark TNFα signaling via NFkB pathway with baseline serum TNFα

TNF, Tumor necrosis factor; IO360, Immuno-oncology 360.

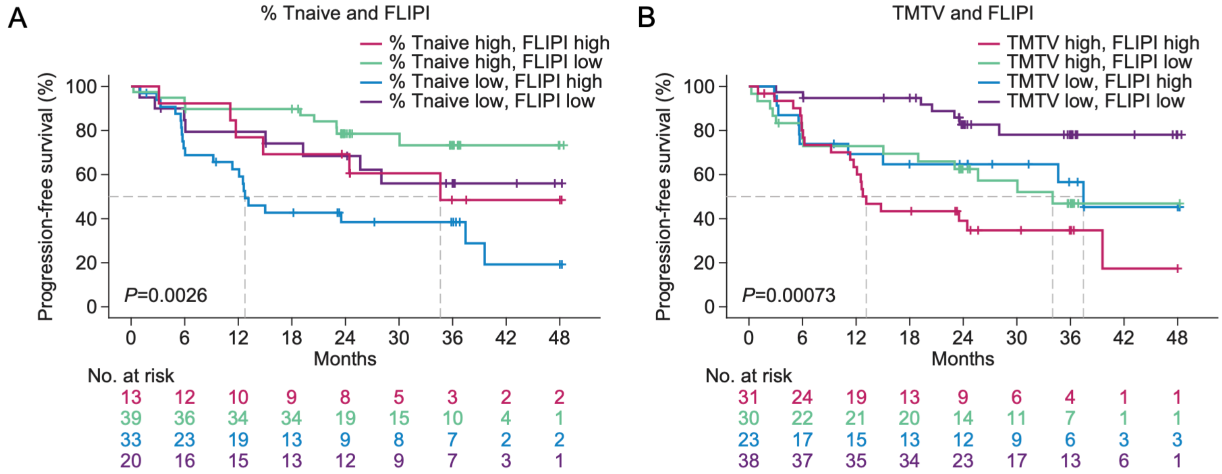


**Figure S11. Risk stratification of FL patients using percent naïve or TMTV with FLIPI score**
(A) KM-estimated PFS by median of percentage of naïve CAR T cells in product and FLIPI score. (B) KM-estimated PFS by median of baseline TMTV and FLIPI score.

FLIPI, follicular lymphoma International Prognostic Index; KM, Kaplan Meier; PFS, progression-free survival; TMTV, total metabolic tumor volume. FLIPI high: FLIPI score>2; FLIPI low: FLIPI score≤2; Medians were used for all other variables for high vs. low.

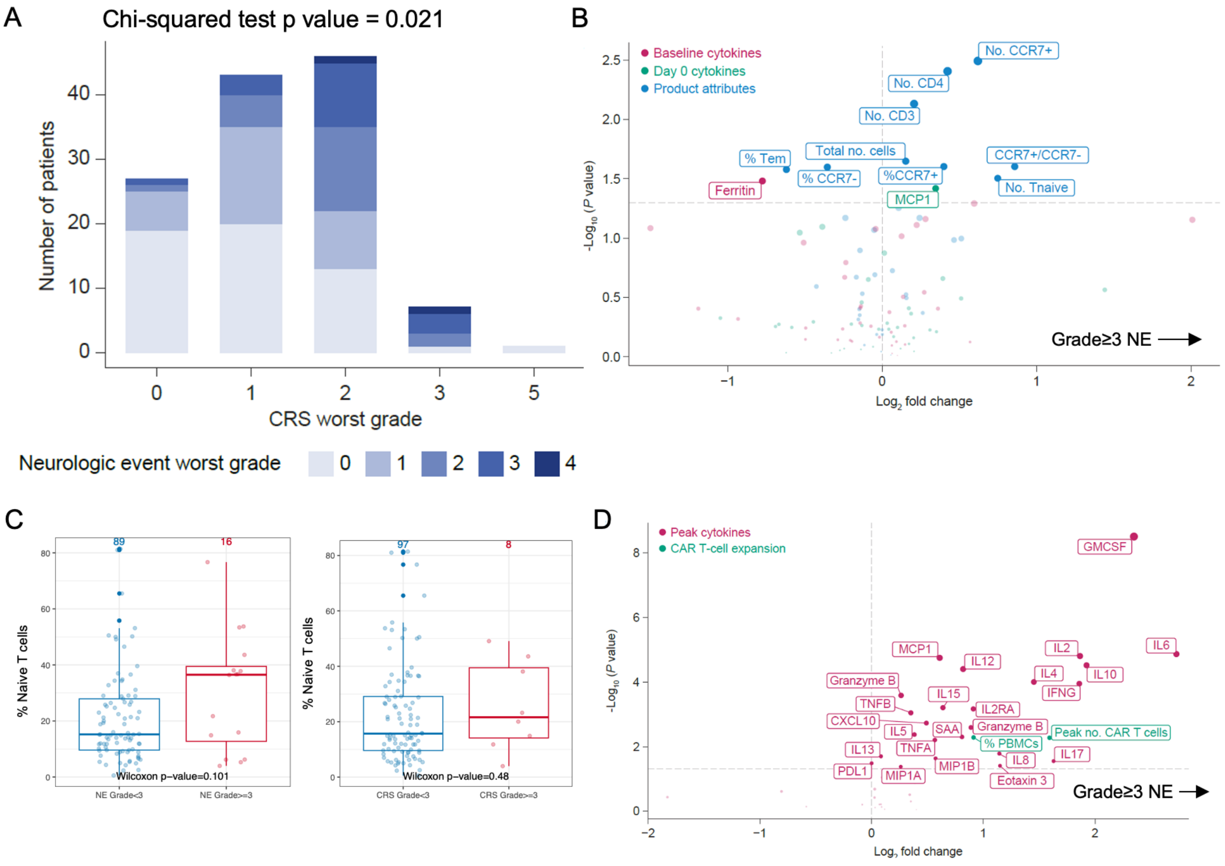


**Figure S12. Univariate analysis of pre-treatment and post-infusion covariates associated with grade≥3 neurologic events**

(A) Association between neurologic events and CRS by grade. (B) Volcano plot showing association of pre-treatment serum cytokines and product attributes with grade ≥3 neurologic events. (C) Percentage of naive T cells in the product by grade of neurologic events or grade of CRS. (D) Volcano plot showing association of post-infusion serum cytokines and peak CAR T-cell levels with grade ≥3 neurologic events.

AE, adverse event; AUC, area under the curve; CAR, chimeric antigen receptor; CCL, C-C motif chemokine ligand; CRS, cytokine release syndrome; CXCL, C-X-C motif chemokine ligand; GM-CSF, granulocyte-macrophage colony-stimulating factor; IFN, interferon; IL, interleukin; MCP-1, monocyte chemoattractant protein-1; NE, neurologic events; PD-L1, programmed death-ligand 1; PMBC, peripheral blood mononuclear cell; SAA, serum amyloid A; SPD, sum of the product of diameters; Tcm, central memory T cells; Teff, effector memory T cells; TNF, tumor necrosis factor; VCAM, vascular cell adhesion molecule.

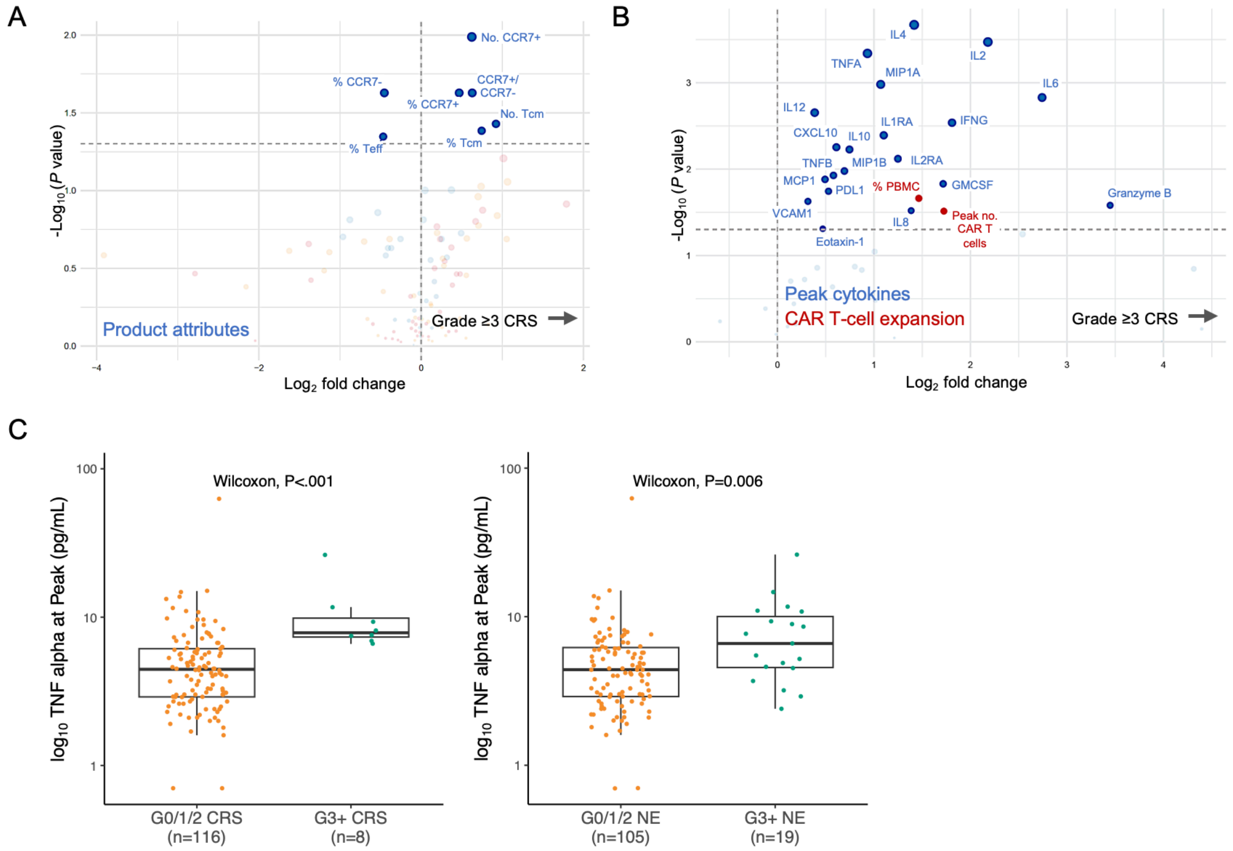


**Fig. S13. Univariate analysis of pre-treatment and post-infusion covariates associated with grade≥3 cytokine release syndrome**

(A) Volcano plot showing associations of pre-treatment serum cytokines and product attributes with grade ≥3 CRS. (B) Volcano plot showing associations of serum cytokines and peak CAR T-cell levels with grade ≥3 CRS. (C) Peak TNFα levels in serum within first 28 days by grade of neurologic events and CRS.

AE, adverse event; AUC, area under the curve; CAR, chimeric antigen receptor; CCL, C-C motif chemokine ligand; CRS, cytokine release syndrome; CXCL, C-X-C motif chemokine ligand; GM-CSF, granulocyte-macrophage colony-stimulating factor; IFN, interferon; IL, interleukin; MCP-1, monocyte chemoattractant protein-1; NE, neurologic events; PD-L1, programmed death-ligand 1; PMBC, peripheral blood mononuclear cell; SAA, serum amyloid A; SPD, sum of the product of diameters; Tcm, central memory T cells; Teff, effector memory T cells; TNF, tumor necrosis factor; VCAM, vascular cell adhesion molecule.

**Supplementary Table 1. List or pre-treatment covariates**

| variable | description |
|---|---|
| NOPRCC | Number of Prior Lines of Therapy (N) |
| BASEWT | Baseline Weight |
| BTBURC01 | Baseline Tumor Bur (SPD) (mm^2) - Cen.01 |
| CD19HS01 | CD19 Antigen HSCORE period 01 |
| stage_n | Disease Stage |
| score_n | International Prognostic Index (IPI) |
| blecog_n | Baseline ECOG Performance Status |
| bulkdis_n | Bulky Disease Y/N |
| steroid_use_n | Steroid usage (y/n) |
| tocilizumab_use_n | Tocilizumab usage (y/n) |
| AGE | Age |
| Male | Male |
| ALB_DAY0 | Albumin (g/L) at day 0 |
| ALP_DAY0 | Alkaline Phosphatase (U/L) at day 0 |
| ALT_DAY0 | Alanine Aminotransferase (U/L) at day 0 |
| AST_DAY0 | Aspartate Aminotransferase (U/L) at day 0 |
| BICARB_DAY0 | Bicarbonate (mmol/L) at day 0 |
| BILDIR_DAY0 | Direct Bilirubin (umol/L) at day 0 |
| BILI_DAY0 | Bilirubin (umol/L) at day 0 |
| CA_DAY0 | Calcium (mmol/L) at day 0 |
| CL_DAY0 | Chloride (mmol/L) at day 0 |
| CREAT_DAY0 | Creatinine (umol/L) at day 0 |
| CREATCLR_DAY0 | Creatinine Clearance (mL/min) at day 0 |
| GLUC_DAY0 | Glucose (mmol/L) at day 0 |
| K_DAY0 | Potassium (mmol/L) at day 0 |
| LDH_DAY0 | Lactate Dehydrogenase (U/L) at day 0 |
| MG_DAY0 | Magnesium (mmol/L) at day 0 |
| PHOS_DAY0 | Phosphate (mmol/L) at day 0 |
| SODIUM_DAY0 | Sodium (mmol/L) at day 0 |
| URATE_DAY0 | Urate (mmol/L) at day 0 |
| UREAN_DAY0 | Urea Nitrogen (mmol/L) at day 0 |
| HGB_DAY0 | Hemoglobin (mmol/L) at day 0 |
| LYM_DAY0 | Lymphocytes (10^9/L) at day 0 |
| LYMLE_DAY0 | Lymphocytes/Leukocytes (fraction of 1) at day 0 |
| MONO_DAY0 | Monocytes (10^9/L) at day 0 |
| MONOLE_DAY0 | Monocytes/Leukocytes (fraction of 1) at day 0 |
| NEUTLE_DAY0 | Neutrophils/Leukocytes (fraction of 1) at day 0 |

| | |
|---|---|
| PLAT_DAY0 | Platelets (10^9/L) at day 0 |
| WBC_DAY0 | Leukocytes (10^9/L) at day 0 |
| ALB_BASELINE | Albumin (g/L) at baseline |
| ALP_BASELINE | Alkaline Phosphatase (U/L) at baseline |
| ALT_BASELINE | Alanine Aminotransferase (U/L) at baseline |
| AST_BASELINE | Aspartate Aminotransferase (U/L) at baseline |
| BICARB_BASELINE | Bicarbonate (mmol/L) at baseline |
| BILDIR_BASELINE | Direct Bilirubin (umol/L) at baseline |
| BILI_BASELINE | Bilirubin (umol/L) at baseline |
| CA_BASELINE | Calcium (mmol/L) at baseline |
| CL_BASELINE | Chloride (mmol/L) at baseline |
| CREAT_BASELINE | Creatinine (umol/L) at baseline |
| CREATCLR_BASELINE | Creatinine Clearance (mL/min) at baseline |
| GLUC_BASELINE | Glucose (mmol/L) at baseline |
| K_BASELINE | Potassium (mmol/L) at baseline |
| LDH_BASELINE | Lactate Dehydrogenase (U/L) at baseline |
| MG_BASELINE | Magnesium (mmol/L) at baseline |
| PHOS_BASELINE | Phosphate (mmol/L) at baseline |
| SODIUM_BASELINE | Sodium (mmol/L) at baseline |
| URATE_BASELINE | Urate (mmol/L) at baseline |
| UREAN_BASELINE | Urea Nitrogen (mmol/L) at baseline |
| HGB_BASELINE | Hemoglobin (mmol/L) at baseline |
| LYM_BASELINE | Lymphocytes (10^9/L) at baseline |
| LYMLE_BASELINE | Lymphocytes/Leukocytes (fraction of 1) at baseline |
| MONO_BASELINE | Monocytes (10^9/L) at baseline |
| MONOLE_BASELINE | Monocytes/Leukocytes (fraction of 1) at baseline |
| NEUT_BASELINE | Neutrophils (10^9/L) at baseline |
| NEUTLE_BASELINE | Neutrophils/Leukocytes (fraction of 1) at baseline |
| PLAT_BASELINE | Platelets (10^9/L) at baseline |
| WBC_BASELINE | Leukocytes (10^9/L) at baseline |
| ALB_D0_FCHG_B | Albumin (g/L) fold change |
| ALP_D0_FCHG_B | Alkaline Phosphatase (U/L) fold change |
| ALT_D0_FCHG_B | Alanine Aminotransferase (U/L) fold change |
| AST_D0_FCHG_B | Aspartate Aminotransferase (U/L) fold change |
| BICARB_D0_FCHG_B | Bicarbonate (mmol/L) fold change |
| BILDIR_D0_FCHG_B | Direct Bilirubin (umol/L) fold change |
| BILI_D0_FCHG_B | Bilirubin (umol/L) fold change |
| CA_D0_FCHG_B | Calcium (mmol/L) fold change |
| CL_D0_FCHG_B | Chloride (mmol/L) fold change |
| CREAT_D0_FCHG_B | Creatinine (umol/L) fold change |

| | |
|---|---|
| CREATCLR_D0_FCHG_B | Creatinine Clearance (mL/min) fold change |
| GLUC_D0_FCHG_B | Glucose (mmol/L) fold change |
| K_D0_FCHG_B | Potassium (mmol/L) fold change |
| LDH_D0_FCHG_B | Lactate Dehydrogenase (U/L) fold change |
| MG_D0_FCHG_B | Magnesium (mmol/L) fold change |
| PHOS_D0_FCHG_B | Phosphate (mmol/L) fold change |
| SODIUM_D0_FCHG_B | Sodium (mmol/L) fold change |
| URATE_D0_FCHG_B | Urate (mmol/L) fold change |
| UREAN_D0_FCHG_B | Urea Nitrogen (mmol/L) fold change |
| HGB_D0_FCHG_B | Hemoglobin (mmol/L) fold change |
| LYM_D0_FCHG_B | Lymphocytes (10^9/L) fold change |
| LYMLE_D0_FCHG_B | Lymphocytes/Leukocytes (fraction of 1) fold change |
| NEUTLE_D0_FCHG_B | Neutrophils/Leukocytes (fraction of 1) fold change |
| PLAT_D0_FCHG_B | Platelets (10^9/L) fold change |
| WBC_D0_FCHG_B | Leukocytes (10^9/L) fold change |
| DOUBTIME_BASELINE | Doubling Time (days) at baseline |
| IFNGCOC_BASELINE | Interferon Gamma by coculture (pg/mL) at baseline |
| MFGDOSE_BASELINE | Manufacturing Dose Produced at baseline |
| NCD3_BASELINE | Number of CD3 Cells (10^6) at baseline |
| NCD3N_BASELINE | Number of CD3- Cells (10^6) at baseline |
| NCD3N_BASELINE | Number of CD3- Cells (10^6) at baseline |
| NCD4_BASELINE | Number of CD4 Cells (10^6) at baseline |
| NCD8_BASELINE | Number of CD8 Cells (10^6) at baseline |
| NCM_BASELINE | Number of Central Memory Cells (10^6) at baseline |
| NEEM_BASELINE | Number of Effector and Effector Memory T Cells (10^6) at baseline |
| NEF_BASELINE | Number of Effector T Cells (10^6) at baseline |
| NEM_BASELINE | Number of Effector Memory T Cells (10^6) at baseline |
| NNCM_BASELINE | Number of Naïve Cells and Central Memory Cells (10^6) at baseline |
| NNV_BASELINE | Number of Naïve Cells (10^6) at baseline |
| PCD4_BASELINE | Percentage of CD4 in viable CD3 cells (%) at baseline |
| PCD4_BASELINE | Percentage of CD4 in viable CD3 cells (%) at baseline |
| PCM_BASELINE | Percentage of Central Memory Cells in viable CD3 cells (%) at baseline |
| PEEM_BASELINE | Percent of Effector and Effector Memory T Cells (%) at baseline |
| PEEM_BASELINE | Percent of Effector and Effector Memory T Cells (%) at baseline |
| PEF_BASELINE | Effector Cells at baseline |
| TRDEFF_BASELINE | Transduction Efficiency at baseline |
| VECTCPNM_BASELINE | Vector Copy Number at baseline |
| VIABILTY_BASELINE | Viability at baseline |
| CRP_BASELINE | C Reactive Protein (mg/L) at baseline |
| CXCL10_BASELINE | Chemokine (C-X-C Motif) Ligand 10 (pg/mL) at baseline |

| | |
|---|---|
| EOTAXIN1_BASELINE | Eotaxin-1 (pg/mL) at baseline |
| EOTAXIN3_BASELINE | Eotaxin-3 (pg/mL) at baseline |
| FERRITIN_BASELINE | Ferritin (ng/mL) at baseline |
| GRZA_BASELINE | Granzyme A (pg/mL) at baseline |
| GRZAE_BASELINE | Granzyme A Ella (pg/mL) at baseline |
| GRZB_BASELINE | Granzyme B (pg/mL) at baseline |
| GRZBE_BASELINE | Granzyme B Ella (pg/mL) at baseline |
| ICAM1CU_BASELINE | Intercellular Adhesion Molecule 1 (ng/mL) at baseline |
| IFNG_BASELINE | Interferon Gamma (pg/mL) at baseline |
| INTLK1RA_BASELINE | Interleukin 1 Receptor Antagonist (pg/mL) at baseline |
| INTLK10_BASELINE | Interleukin 10 (pg/mL) at baseline |
| IL122340_BASELINE | Interleukin 12+23 p40 (pg/mL) at baseline |
| INTLK15_BASELINE | Interleukin 15 (pg/mL) at baseline |
| INTLK16_BASELINE | Interleukin 16 (pg/mL) at baseline |
| INTLK17_BASELINE | Interleukin 17 (pg/mL) at baseline |
| INTLK2_BASELINE | Interleukin 2 (pg/mL) at baseline |
| INTLK6_BASELINE | Interleukin 6 (pg/mL) at baseline |
| INTLK7_BASELINE | Interleukin 7 (pg/mL) at baseline |
| INTLK8_BASELINE | Interleukin 8 (pg/mL) at baseline |
| MCP1_BASELINE | Monocyte Chemotactic Protein 1 (pg/mL) at baseline |
| MCP4_BASELINE | Monocyte Chemotactic Protein 4 (pg/mL) at baseline |
| MDC_BASELINE | Macrophage-Derived Chemokine (pg/mL) at baseline |
| MIP1A_BASELINE | Macrophage Inflammatory Protein 1 Alpha (pg/mL) at baseline |
| MIP1B_BASELINE | Macrophage Inflammatory Protein 1 Beta (pg/mL) at baseline |
| PRFCU_BASELINE | Perforin (ng/mL) at baseline |
| AMYLOIDA_BASELINE | Amyloid A (pg/mL) at baseline |
| SFASL_BASELINE | CD95 Ligand (pg/mL) at baseline |
| CCL17_BASELINE | Chemokine (C-C Motif) Ligand 17 (pg/mL) at baseline |
| TNFA_BASELINE | Tumor Necrosis Factor Alpha (pg/mL) at baseline |
| TNFB_BASELINE | Tumor Necrosis Factor Beta (pg/mL) at baseline |
| VCAM1CU_BASELINE | Vascular Cell Adhesion Molecule 1 (ng/mL) at baseline |
| VEGF_BASELINE | Vascular Endothelial Growth Factor (pg/mL) at baseline |
| CRP_DAY0 | C Reactive Protein (mg/L) at day 0 |
| CXCL10_DAY0 | Chemokine (C-X-C Motif) Ligand 10 (pg/mL) at day 0 |
| EOTAXIN1_DAY0 | Eotaxin-1 (pg/mL) at day 0 |
| EOTAXIN3_DAY0 | Eotaxin-3 (pg/mL) at day 0 |
| FERRITIN_DAY0 | Ferritin (ng/mL) at day 0 |
| GRZA_DAY0 | Granzyme A (pg/mL) at day 0 |
| GRZAE_DAY0 | Granzyme A Ella (pg/mL) at day 0 |
| GRZB_DAY0 | Granzyme B (pg/mL) at day 0 |

| | |
|---|---|
| GRZBE_DAY0 | Granzyme B Ella (pg/mL) at day 0 |
| ICAM1CU_DAY0 | Intercellular Adhesion Molecule 1 (ng/mL) at day 0 |
| IFNG_DAY0 | Interferon Gamma (pg/mL) at day 0 |
| INTLK1RA_DAY0 | Interleukin 1 Receptor Antagonist (pg/mL) at day 0 |
| INTLK10_DAY0 | Interleukin 10 (pg/mL) at day 0 |
| IL122340_DAY0 | Interleukin 12+23 p40 (pg/mL) at day 0 |
| INTLK15_DAY0 | Interleukin 15 (pg/mL) at day 0 |
| INTLK16_DAY0 | Interleukin 16 (pg/mL) at day 0 |
| INTLK17_DAY0 | Interleukin 17 (pg/mL) at day 0 |
| INTLK2_DAY0 | Interleukin 2 (pg/mL) at day 0 |
| INTL2RCU_DAY0 | Interleukin 2 Receptor Alpha (ng/mL) at day 0 |
| INTLK6_DAY0 | Interleukin 6 (pg/mL) at day 0 |
| INTLK7_DAY0 | Interleukin 7 (pg/mL) at day 0 |
| INTLK8_DAY0 | Interleukin 8 (pg/mL) at day 0 |
| MCP1_DAY0 | Monocyte Chemotactic Protein 1 (pg/mL) at day 0 |
| MCP4_DAY0 | Monocyte Chemotactic Protein 4 (pg/mL) at day 0 |
| MDC_DAY0 | Macrophage-Derived Chemokine (pg/mL) at day 0 |
| MIP1A_DAY0 | Macrophage Inflammatory Protein 1 Alpha (pg/mL) at day 0 |
| MIP1B_DAY0 | Macrophage Inflammatory Protein 1 Beta (pg/mL) at day 0 |
| PDL1_DAY0 | Programmed Death Ligand 1 (pg/mL) at day 0 |
| PRFCU_DAY0 | Perforin (ng/mL) at day 0 |
| AMYLOIDA_DAY0 | Amyloid A (pg/mL) at day 0 |
| SFASL_DAY0 | CD95 Ligand (pg/mL) at day 0 |
| CCL17_DAY0 | Chemokine (C-C Motif) Ligand 17 (pg/mL) at day 0 |
| TNFA_DAY0 | Tumor Necrosis Factor Alpha (pg/mL) at day 0 |
| VCAM1CU_DAY0 | Vascular Cell Adhesion Molecule 1 (ng/mL) at day 0 |
| VEGF_DAY0 | Vascular Endothelial Growth Factor (pg/mL) at day 0 |
| CRP_D0_FCHG_B | C Reactive Protein (mg/L) fold change |
| CXCL10_D0_FCHG_B | Chemokine (C-X-C Motif) Ligand 10 (pg/mL) fold change |
| EOTAXIN1_D0_FCHG_B | Eotaxin-1 (pg/mL) fold change |
| EOTAXIN3_D0_FCHG_B | Eotaxin-3 (pg/mL) fold change |
| FERRITIN_D0_FCHG_B | Ferritin (ng/mL) fold change |
| GRZA_D0_FCHG_B | Granzyme A (pg/mL) fold change |
| GRZAE_D0_FCHG_B | Granzyme A Ella (pg/mL) fold change |
| GRZB_D0_FCHG_B | Granzyme B (pg/mL) fold change |
| GRZBE_D0_FCHG_B | Granzyme B Ella (pg/mL) fold change |
| ICAM1CU_D0_FCHG_B | Intercellular Adhesion Molecule 1 (ng/mL) fold change |
| IFNG_D0_FCHG_B | Interferon Gamma (pg/mL) fold change |
| INTLK1RA_D0_FCHG_B | Interleukin 1 Receptor Antagonist (pg/mL) fold change |
| INTLK10_D0_FCHG_B | Interleukin 10 (pg/mL) fold change |

| | |
|---|---|
| IL122340_D0_FCHG_B | Interleukin 12+23 p40 (pg/mL) fold change |
| INTLK15_D0_FCHG_B | Interleukin 15 (pg/mL) fold change |
| INTLK16_D0_FCHG_B | Interleukin 16 (pg/mL) fold change |
| INTLK17_D0_FCHG_B | Interleukin 17 (pg/mL) fold change |
| INTLK2_D0_FCHG_B | Interleukin 2 (pg/mL) fold change |
| INTL2RCU_D0_FCHG_B | Interleukin 2 Receptor Alpha (ng/mL) fold change |
| INTLK6_D0_FCHG_B | Interleukin 6 (pg/mL) fold change |
| INTLK7_D0_FCHG_B | Interleukin 7 (pg/mL) fold change |
| INTLK8_D0_FCHG_B | Interleukin 8 (pg/mL) fold change |
| MCP1_D0_FCHG_B | Monocyte Chemotactic Protein 1 (pg/mL) fold change |
| MCP4_D0_FCHG_B | Monocyte Chemotactic Protein 4 (pg/mL) fold change |
| MDC_D0_FCHG_B | Macrophage-Derived Chemokine (pg/mL) fold change |
| MIP1A_D0_FCHG_B | Macrophage Inflammatory Protein 1 Alpha (pg/mL) fold change |
| MIP1B_D0_FCHG_B | Macrophage Inflammatory Protein 1 Beta (pg/mL) fold change |
| PDL1_D0_FCHG_B | Programmed Death Ligand 1 (pg/mL) fold change |
| PRFCU_D0_FCHG_B | Perforin (ng/mL) fold change |
| AMYLOIDA_D0_FCHG_B | Amyloid A (pg/mL) fold change |
| SFASL_D0_FCHG_B | CD95 Ligand (pg/mL) fold change |
| CCL17_D0_FCHG_B | Chemokine (C-C Motif) Ligand 17 (pg/mL) fold change |
| TNFA_D0_FCHG_B | Tumor Necrosis Factor Alpha (pg/mL) fold change |
| VCAM1CU_D0_FCHG_B | Vascular Cell Adhesion Molecule 1 (ng/mL) fold change |
| VEGF_D0_FCHG_B | Vascular Endothelial Growth Factor (pg/mL) fold change |
| PRASCT | Prior Auto. Stem Cell Transplant (ASCT) |
| PI3KIHFL | Prior PI3K inhibitor |
| PBTKIHFL | Prior BTK inhibitor |
| OBIMABFL | Prior Med-Obinutuzumb |
| BENTINFL | Prior Med-Bendamustine |
| ANTAGFL | Prior Med-Anti-CD20 single agent |
| ALKFL | Prior Med-Alkylating Agent |
| ANTALKFL | Prior Med-Anti-CD20 + Alkylating Agent |
| LENDFL | Prior Med-Lenalidomide |
| HRVSTDY_PROD | Harvest day |
| MTVAVAL | Metabolic Tumor Volume Results |

**Supplementary Table 2. All covariates included/excluded in the multivariate analysis**

| Variable | Description | n missing | high missing | zero variance | High corr | Corr var | Cor | Included |
|---|---|---|---|---|---|---|---|---|
| NOPRCC | Number of Prior Lines of Therapy (N) | 1 | | | | | | Yes |
| BASEWT | Baseline Weight | 0 | | | | | | Yes |
| BTBURC01 | Baseline Tumor Bur (SPD) (mm^2) - Cen.01 | 6 | | | | | | Yes |
| CD19HS01 | CD19 Antigen HSCORE period 01 | 19 | | | | | | Yes |
| stage_n | Disease Stage | 0 | | | | | | Yes |
| score_n | International Prognostic Index (IPI) | 0 | | | | | | Yes |
| blecog_n | Baseline ECOG Performance Status | 0 | | | | | | Yes |
| bulkdis_n | Bulky Disease Y/N | 0 | | | | | | Yes |
| steroid_use_n | Steroid usage (y/n) | 0 | | | | | | Yes |
| tocilizumab_use_n | Tocilizumab usage (y/n) | 0 | | | | | | Yes |
| AGE | Age | 0 | | | | | | Yes |
| Male | Male | 0 | | | | | | Yes |
| ALB_DAY0 | Albumin (g/L) at day 0 | 1 | | | | | | Yes |
| ALP_DAY0 | Alkaline Phosphatase (U/L) at day 0 | 1 | | | | | | Yes |
| ALT_DAY0 | Alanine Aminotransferase (U/L) at day 0 | 1 | | | | | | Yes |
| AST_DAY0 | Aspartate Aminotransferase (U/L) at day 0 | 1 | | | | | | Yes |
| BICARB_DAY0 | Bicarbonate (mmol/L) at day 0 | 0 | | | | | | Yes |

| | | | | | | | | |
|---|---|---|---|---|---|---|---|---|
| BILDIR_DAY0 | Direct Bilirubin (umol/L) at day 0 | 19 | | | | | | Yes |
| BILI_DAY0 | Bilirubin (umol/L) at day 0 | 1 | | | | | | Yes |
| CA_DAY0 | Calcium (mmol/L) at day 0 | 0 | | | | | | Yes |
| CL_DAY0 | Chloride (mmol/L) at day 0 | 0 | | | | | | Yes |
| CREAT_DAY0 | Creatinine (umol/L) at day 0 | 0 | | | | | | Yes |
| CREATCLR_DAY0 | Creatinine Clearance (mL/min) at day 0 | 13 | | | | | | Yes |
| GLUC_DAY0 | Glucose (mmol/L) at day 0 | 0 | | | | | | Yes |
| K_DAY0 | Potassium (mmol/L) at day 0 | 0 | | | | | | Yes |
| LDH_DAY0 | Lactate Dehydrogenase (U/L) at day 0 | 3 | | | | | | Yes |
| MG_DAY0 | Magnesium (mmol/L) at day 0 | 4 | | | | | | Yes |
| PHOS_DAY0 | Phosphate (mmol/L) at day 0 | 5 | | | | | | Yes |
| SODIUM_DAY0 | Sodium (mmol/L) at day 0 | 0 | | | | | | Yes |
| URATE_DAY0 | Urate (mmol/L) at day 0 | 4 | | | | | | Yes |
| UREA_DAY0 | Urea (mmol/L) at day 0 | 114 | Y | | | | | No |
| UREAN_DAY0 | Urea Nitrogen (mmol/L) at day 0 | 10 | | | | | | Yes |
| HCT_DAY0 | Hematocrit (fraction of 1) at day 0 | 0 | | | Y | HGB_DAY0 | 0.976109153 | No |

| | | | | | | | | |
|---|---|---|---|---|---|---|---|---|
| HGB_DAY0 | Hemoglobin (mmol/L) at day 0 | 0 | | | | | | Yes |
| LYM_DAY0 | Lymphocytes (10^9/L) at day 0 | 5 | | | | | | Yes |
| LYMLE_DAY0 | Lymphocytes/Leukocytes (fraction of 1) at day 0 | 5 | | | | | | Yes |
| MONO_DAY0 | Monocytes (10^9/L) at day 0 | 7 | | | | | | Yes |
| MONOLE_DAY0 | Monocytes/Leukocytes (fraction of 1) at day 0 | 5 | | | | | | Yes |
| NEUT_DAY0 | Neutrophils (10^9/L) at day 0 | 5 | | | Y | WBC_DAY0 | 0.962026581 | No |
| NEUTLE_DAY0 | Neutrophils/Leukocytes (fraction of 1) at day 0 | 5 | | | | | | Yes |
| PLAT_DAY0 | Platelets (10^9/L) at day 0 | 1 | | | | | | Yes |
| WBC_DAY0 | Leukocytes (10^9/L) at day 0 | 0 | | | | | | Yes |
| ALB_BASELINE | Albumin (g/L) at baseline | 0 | | | | | | Yes |
| ALP_BASELINE | Alkaline Phosphatase (U/L) at baseline | 0 | | | | | | Yes |
| ALT_BASELINE | Alanine Aminotransferase (U/L) at baseline | 0 | | | | | | Yes |
| AST_BASELINE | Aspartate Aminotransferase (U/L) at baseline | 0 | | | | | | Yes |
| BICARB_BASELINE | Bicarbonate (mmol/L) at baseline | 0 | | | | | | Yes |
| BILDIR_BASELINE | Direct Bilirubin (umol/L) at baseline | 10 | | | | | | Yes |

| | | | | | | | | |
|---|---|---|---|---|---|---|---|---|
| BILI_BASELINE | Bilirubin (umol/L) at baseline | 0 | | | | | | Yes |
| CA_BASELINE | Calcium (mmol/L) at baseline | 0 | | | | | | Yes |
| CL_BASELINE | Chloride (mmol/L) at baseline | 0 | | | | | | Yes |
| CREAT_BASELINE | Creatinine (umol/L) at baseline | 0 | | | | | | Yes |
| CREATCLR_BASELINE | Creatinine Clearance (mL/min) at baseline | 0 | | | | | | Yes |
| GLUC_BASELINE | Glucose (mmol/L) at baseline | 0 | | | | | | Yes |
| K_BASELINE | Potassium (mmol/L) at baseline | 0 | | | | | | Yes |
| LDH_BASELINE | Lactate Dehydrogenase (U/L) at baseline | 0 | | | | | | Yes |
| MG_BASELINE | Magnesium (mmol/L) at baseline | 0 | | | | | | Yes |
| PHOS_BASELINE | Phosphate (mmol/L) at baseline | 1 | | | | | | Yes |
| SODIUM_BASELINE | Sodium (mmol/L) at baseline | 0 | | | | | | Yes |
| URATE_BASELINE | Urate (mmol/L) at baseline | 1 | | | | | | Yes |
| UREA_BASELINE | Urea (mmol/L) at baseline | 114 | Y | | | | | No |
| UREAN_BASELINE | Urea Nitrogen (mmol/L) at baseline | 10 | | | | | | Yes |
| HCT_BASELINE | Hematocrit (fraction of 1) at baseline | 0 | | | Y | HGB_BASELINE | 0.95652914 | No |
| HGB_BASELINE | Hemoglobin (mmol/L) at baseline | 0 | | | | | | Yes |

| | | | | | | | | |
|---|---|---|---|---|---|---|---|---|
| LYM_BASELINE | Lymphocytes (10^9/L) at baseline | 0 | | | | | | Yes |
| LYMLE_BASELINE | Lymphocytes/Leukocytes (fraction of 1) at baseline | 0 | | | | | | Yes |
| MONO_BASELINE | Monocytes (10^9/L) at baseline | 2 | | | | | | Yes |
| MONOLE_BASELINE | Monocytes/Leukocytes (fraction of 1) at baseline | 0 | | | | | | Yes |
| NEUT_BASELINE | Neutrophils (10^9/L) at baseline | 0 | | | | | | Yes |
| NEUTLE_BASELINE | Neutrophils/Leukocytes (fraction of 1) at baseline | 0 | | | | | | Yes |
| PLAT_BASELINE | Platelets (10^9/L) at baseline | 0 | | | | | | Yes |
| WBC_BASELINE | Leukocytes (10^9/L) at baseline | 0 | | | | | | Yes |
| ALB_D0_FCHG_B | Albumin (g/L) fold change | 1 | | | | | | Yes |
| ALP_D0_FCHG_B | Alkaline Phosphatase (U/L) fold change | 1 | | | | | | Yes |
| ALT_D0_FCHG_B | Alanine Aminotransferase (U/L) fold change | 1 | | | | | | Yes |
| AST_D0_FCHG_B | Aspartate Aminotransferase (U/L) fold change | 1 | | | | | | Yes |
| BICARB_D0_FCHG_B | Bicarbonate (mmol/L) fold change | 0 | | | | | | Yes |
| BILDIR_D0_FCHG_B | Direct Bilirubin (umol/L) fold change | 21 | | | | | | Yes |
| BILI_D0_FCHG_B | Bilirubin (umol/L) fold change | 1 | | | | | | Yes |

| | | | | | | | | |
|---|---|---|---|---|---|---|---|---|
| CA_D0_FCHG_B | Calcium (mmol/L) fold change | 0 | | | | | | Yes |
| CL_D0_FCHG_B | Chloride (mmol/L) fold change | 0 | | | | | | Yes |
| CREAT_D0_FCHG_B | Creatinine (umol/L) fold change | 0 | | | | | | Yes |
| CREATCLR_D0_FCHG_B | Creatinine Clearance (mL/min) fold change | 13 | | | | | | Yes |
| GLUC_D0_FCHG_B | Glucose (mmol/L) fold change | 0 | | | | | | Yes |
| K_D0_FCHG_B | Potassium (mmol/L) fold change | 0 | | | | | | Yes |
| LDH_D0_FCHG_B | Lactate Dehydrogenase (U/L) fold change | 3 | | | | | | Yes |
| MG_D0_FCHG_B | Magnesium (mmol/L) fold change | 4 | | | | | | Yes |
| PHOS_D0_FCHG_B | Phosphate (mmol/L) fold change | 6 | | | | | | Yes |
| SODIUM_D0_FCHG_B | Sodium (mmol/L) fold change | 0 | | | | | | Yes |
| URATE_D0_FCHG_B | Urate (mmol/L) fold change | 5 | | | | | | Yes |
| UREA_D0_FCHG_B | Urea (mmol/L) fold change | 114 | Y | | | | | No |
| UREAN_D0_FCHG_B | Urea Nitrogen (mmol/L) fold change | 10 | | | | | | Yes |
| HCT_D0_FCHG_B | Hematocrit (fraction of 1) fold change | 0 | | | Y | HGB_D0_FCHG_B | 0.954835583 | No |
| HGB_D0_FCHG_B | Hemoglobin (mmol/L) fold change | 0 | | | | | | Yes |
| LYM_D0_FCHG_B | Lymphocytes (10^9/L) fold change | 5 | | | | | | Yes |

| | | | | | | | | |
|---|---|---|---|---|---|---|---|---|
| LYMLE_D0_FCHG_B | Lymphocytes/Leukocytes (fraction of 1) fold change | 5 | | | | | | Yes |
| MONO_D0_FCHG_B | Monocytes (10^9/L) fold change | 7 | | | Y | MONO_DAY0 | 0.949303266 | No |
| MONOLE_D0_FCHG_B | Monocytes/Leukocytes (fraction of 1) fold change | 5 | | | Y | MONOLE_DAY0 | 0.925338179 | No |
| NEUT_D0_FCHG_B | Neutrophils (10^9/L) fold change | 5 | | | Y | WBC_D0_FCHG_B | 0.962206471 | No |
| NEUTLE_D0_FCHG_B | Neutrophils/Leukocytes (fraction of 1) fold change | 5 | | | | | | Yes |
| PLAT_D0_FCHG_B | Platelets (10^9/L) fold change | 1 | | | | | | Yes |
| WBC_D0_FCHG_B | Leukocytes (10^9/L) fold change | 0 | | | | | | Yes |
| ncart_PEAKVAL | at peak | 0 | | | Y | ncart_AUCAVAL | 0.977088906 | No |
| ncart_AUCAVAL | AUC | 0 | | | | | | Yes |
| ncart_AUCAVAL | AUC | 0 | | | | | | Yes |
| ppbmc_PEAKVAL | at peak | 0 | | | Y | ppbmc_AUCAVAL | 0.976099022 | No |
| ppbmc_AUCAVAL | AUC | 0 | | | Y | ppbmc_PEAKVAL | 0.976099022 | No |

| | | | | | | | | |
|---|---|---|---|---|---|---|---|---|
| DOUBTIME_BASELINE | Doubling Time (days) at baseline | 0 | | | | | | Yes |
| FLDEXPDY_BASELINE | Fold Expansion per Day at baseline | 0 | | | Y | DOUBTIME_BASELINE | -0.974107279 | No |
| IFNGCOC_BASELINE | Interferon Gamma by coculture (pg/mL) at baseline | 0 | | | | | | Yes |
| IFNGTRDR_BASELINE | IFN-gamma Normalized by Transduction Efficiency (pg/mL) at baseline | 0 | | | Y | IFNGCOC_BASELINE | 0.904201416 | No |
| MFGDOSE_BASELINE | Manufacturing Dose Produced at baseline | 14 | | | | | | Yes |
| NCD3_BASELINE | Number of CD3 Cells (10^6) at baseline | 19 | | | | | | Yes |
| NCD3N_BASELINE | Number of CD3- Cells (10^6) at baseline | 19 | | | | | | Yes |
| NCD3N_BASELINE | Number of CD3- Cells (10^6) at baseline | 19 | | | | | | Yes |
| NCD4_BASELINE | Number of CD4 Cells (10^6) at baseline | 19 | | | | | | Yes |
| NCD8_BASELINE | Number of CD8 Cells (10^6) at baseline | 19 | | | | | | Yes |
| NCM_BASELINE | Number of Central Memory Cells (10^6) at baseline | 19 | | | | | | Yes |
| NEEM_BASELINE | Number of Effector and Effector Memory T Cells (10^6) at baseline | 19 | | | | | | Yes |
| NEF_BASELINE | Number of Effector T Cells (10^6) at baseline | 19 | | | | | | Yes |

| | | | | | | | | |
|---|---|---|---|---|---|---|---|---|
| NEM_BASELINE | Number of Effector Memory T Cells (10^6) at baseline | 19 | | | | | | Yes |
| NNCM_BASELINE | Number of Naïve Cells and Central Memory Cells (10^6) at baseline | 19 | | | | | | Yes |
| NNV_BASELINE | Number of Naïve Cells (10^6) at baseline | 19 | | | | | | Yes |
| PCD3_BASELINE | CD3 Cells at baseline | 19 | | | Y | PCD3N_BASELINE | -1 | No |
| PCD3N_BASELINE | Percent of CD3- Cells (%) at baseline | 19 | | | Y | NCD3N_BASELINE | 0.929009208 | No |
| PCD3N_BASELINE | Percent of CD3- Cells (%) at baseline | 19 | | | Y | PCD3_BASELINE | -1 | No |
| PCD4_BASELINE | Percentage of CD4 in viable CD3 cells (%) at baseline | 19 | | | | | | Yes |
| PCD4_BASELINE | Percentage of CD4 in viable CD3 cells (%) at baseline | 19 | | | | | | Yes |
| PCD8_BASELINE | Percentage of CD8 in viable CD3 cells (%) at baseline | 19 | | | Y | RCD4CD8_BASELINE | -0.999831959 | No |
| PCM_BASELINE | Percentage of Central Memory Cells in viable CD3 cells (%) at baseline | 19 | | | | | | Yes |
| PEEM_BASELINE | Percent of Effector and Effector Memory T Cells (%) at baseline | 19 | | | | | | Yes |

| | | | | | | | | |
|---|---|---|---|---|---|---|---|---|
| PEEM_BASELINE | Percent of Effector and Effector Memory T Cells (%) at baseline | 19 | | | | | | Yes |
| PEF_BASELINE | Effector Cells at baseline | 19 | | | | | | Yes |
| PEM_BASELINE | Effector Memory Cells at baseline | 19 | | | Y | NEM_BASELINE | 0.910772326 | No |
| PNCM_BASELINE | Percent of Naïve Cells and Central Memory Cells in Viable CD3 Cells (%) at baseline | 19 | | | Y | RNCMEEM_BASELINE | 0.999992225 | No |
| PNV_BASELINE | Naive Cells (%) at baseline | 19 | | | Y | NNV_BASELINE | 0.933409708 | No |
| RCD4CD8_BASELINE | CD4/CD8 at baseline | 20 | | | Y | PCD4_BASELINE | 0.999919981 | No |
| RCD4CD8_BASELINE | CD4/CD8 at baseline | 20 | | | Y | PCD8_BASELINE | -0.999831959 | No |
| RNCMEEM_BASELINE | Ratio of Naïve and Central Memory Cells to Effector and Effector Memory Cells at baseline | 19 | | | Y | PNCM_BASELINE | 0.999992225 | No |
| RNCMEEM_BASELINE | Ratio of Naïve and Central Memory Cells to Effector and Effector Memory Cells at baseline | 19 | | | Y | PEEM_BASELINE | -0.99997149 | No |
| TOTCELL_BASELINE | Total CAR + T cells in Product Bag at baseline | 0 | | | Y | BASEWT | 0.96207151 | No |

| | | | | | | | | |
|---|---|---|---|---|---|---|---|---|
| TOTTCELL_BASELINE | Total T Cells (10^6 ) at baseline | 0 | | | Y | NCD3_BASELINE | 0.960313306 | No |
| TRDEFF_BASELINE | Transduction Efficiency at baseline | 0 | | | | | | Yes |
| VECTCPNM_BASELINE | Vector Copy Number at baseline | 7 | | | | | | Yes |
| VIABILTY_BASELINE | Viability at baseline | 0 | | | | | | Yes |
| CRP_BASELINE | | 2 | | | | | | Yes |
| CXCL10_BASELINE | Chemokine (C-X-C Motif) Ligand 10 (pg/mL) at baseline | 2 | | | | | | Yes |
| EOTAXIN1_BASELINE | Eotaxin-1 (pg/mL) at baseline | 2 | | | | | | Yes |
| EOTAXIN3_BASELINE | Eotaxin-3 (pg/mL) at baseline | 2 | | | | | | Yes |
| FERRITIN_BASELINE | Ferritin (ng/mL) at baseline | 2 | | | | | | Yes |
| GMCSF_BASELINE | Granulocyte Macrophage Colony Stm Factor (pg/mL) at baseline | 2 | | Y | | | | No |
| GRZA_BASELINE | Granzyme A (pg/mL) at baseline | 2 | | | | | | Yes |
| GRZAE_BASELINE | Granzyme A Ella (pg/mL) at baseline | 2 | | | | | | Yes |
| GRZB_BASELINE | Granzyme B (pg/mL) at baseline | 2 | | | | | | Yes |
| GRZBE_BASELINE | Granzyme B Ella (pg/mL) at baseline | 2 | | | | | | Yes |
| ICAM1CU_BASELINE | Intercellular Adhesion Molecule 1 (ng/mL) at baseline | 2 | | | | | | Yes |

| | | | | | | | | |
|---|---|---|---|---|---|---|---|---|
| IFNG_BASELINE | Interferon Gamma (pg/mL) at baseline | 2 | | | | | | Yes |
| INTLK1RA_BASELINE | Interleukin 1 Receptor Antagonist (pg/mL) at baseline | 2 | | | | | | Yes |
| INTLK1A_BASELINE | Interleukin 1 Alpha (pg/mL) at baseline | 2 | | Y | | | | No |
| INTLK1B_BASELINE | Interleukin 1 Beta (pg/mL) at baseline | 2 | | Y | | | | No |
| INTLK10_BASELINE | Interleukin 10 (pg/mL) at baseline | 2 | | | | | | Yes |
| IL122340_BASELINE | Interleukin 12+23 p40 (pg/mL) at baseline | 2 | | | | | | Yes |
| INTLK12_BASELINE | Interleukin 12 (pg/mL) at baseline | 2 | | Y | | | | No |
| INTLK13_BASELINE | Interleukin 13 (pg/mL) at baseline | 2 | | Y | | | | No |
| INTLK15_BASELINE | Interleukin 15 (pg/mL) at baseline | 2 | | | | | | Yes |
| INTLK16_BASELINE | Interleukin 16 (pg/mL) at baseline | 2 | | | | | | Yes |
| INTLK17_BASELINE | Interleukin 17 (pg/mL) at baseline | 2 | | | | | | Yes |
| INTLK2_BASELINE | Interleukin 2 (pg/mL) at baseline | 2 | | | | | | Yes |
| INTL2RCU_BASELINE | Interleukin 2 Receptor Alpha (ng/mL) at baseline | 2 | | | Y | INTL2RCU_DAY0 | 0.937793762 | No |
| INTLK4_BASELINE | Interleukin 4 (pg/mL) at baseline | 2 | | Y | | | | No |
| INTLK5_BASELINE | Interleukin 5 (pg/mL) at baseline | 2 | | Y | | | | No |
| INTLK6_BASELINE | Interleukin 6 (pg/mL) at baseline | 2 | | | | | | Yes |

| | | | | | | | | |
|---|---|---|---|---|---|---|---|---|
| INTLK7_BASELINE | Interleukin 7 (pg/mL) at baseline | 2 | | | | | | Yes |
| INTLK8_BASELINE | Interleukin 8 (pg/mL) at baseline | 2 | | | | | | Yes |
| MCP1_BASELINE | Monocyte Chemotactic Protein 1 (pg/mL) at baseline | 2 | | | | | | Yes |
| MCP4_BASELINE | Monocyte Chemotactic Protein 4 (pg/mL) at baseline | 2 | | | | | | Yes |
| MDC_BASELINE | Macrophage-Derived Chemokine (pg/mL) at baseline | 2 | | | | | | Yes |
| MIP1A_BASELINE | Macrophage Inflammatory Protein 1 Alpha (pg/mL) at baseline | 2 | | | | | | Yes |
| MIP1B_BASELINE | Macrophage Inflammatory Protein 1 Beta (pg/mL) at baseline | 2 | | | | | | Yes |
| PDL1_BASELINE | Programmed Death Ligand 1 (pg/mL) at baseline | 2 | | | Y | PDL1_DAY0 | 0.90284939 | No |
| PRFCU_BASELINE | Perforin (ng/mL) at baseline | 2 | | | | | | Yes |
| AMYLOIDA_BASELINE | Amyloid A (pg/mL) at baseline | 2 | | | | | | Yes |
| SFASL_BASELINE | CD95 Ligand (pg/mL) at baseline | 2 | | | | | | Yes |
| CCL17_BASELINE | Chemokine (C-C Motif) Ligand 17 (pg/mL) at baseline | 2 | | | | | | Yes |
| TNFA_BASELINE | Tumor Necrosis Factor Alpha (pg/mL) at baseline | 2 | | | | | | Yes |

| | | | | | | | | |
|---|---|---|---|---|---|---|---|---|
| TNFB_BASELINE | Tumor Necrosis Factor Beta (pg/mL) at baseline | 2 | | | | | | Yes |
| VCAM1CU_BASELINE | Vascular Cell Adhesion Molecule 1 (ng/mL) at baseline | 2 | | | | | | Yes |
| VEGF_BASELINE | Vascular Endothelial Growth Factor (pg/mL) at baseline | 2 | | | | | | Yes |
| GP130_BASELINE | Glycoprotein 130 (pg/mL) at baseline | 124 | Y | | | | | No |
| INTLK6RA_BASELINE | Interleukin 6 Receptor Alpha (pg/mL) at baseline | 124 | Y | | | | | No |
| RANTES_BASELINE | Regulated upon Activation, Normal T Cell Expressed and Persumably Secreted (pg/mL) at baseline | 124 | Y | | | | | No |
| CRP_DAY0 | C Reactive Protein (mg/L) at day 0 | 3 | | | | | | Yes |
| CXCL10_DAY0 | Chemokine (C-X-C Motif) Ligand 10 (pg/mL) at day 0 | 3 | | | | | | Yes |
| EOTAXIN1_DAY0 | Eotaxin-1 (pg/mL) at day 0 | 3 | | | | | | Yes |
| EOTAXIN3_DAY0 | Eotaxin-3 (pg/mL) at day 0 | 3 | | | | | | Yes |
| FERRITIN_DAY0 | Ferritin (ng/mL) at day 0 | 3 | | | | | | Yes |
| GMCSF_DAY0 | Granulocyte Macrophage Colony Stm Factor (pg/mL) at day 0 | 3 | | Y | | | | No |
| GRZA_DAY0 | Granzyme A (pg/mL) at day 0 | 3 | | | | | | Yes |

| | | | | | | | | |
|---|---|---|---|---|---|---|---|---|
| GRZAE_DAY0 | Granzyme A Ella (pg/mL) at day 0 | 3 | | | | | | Yes |
| GRZB_DAY0 | Granzyme B (pg/mL) at day 0 | 3 | | | | | | Yes |
| GRZBE_DAY0 | Granzyme B Ella (pg/mL) at day 0 | 3 | | | | | | Yes |
| ICAM1CU_DAY0 | Intercellular Adhesion Molecule 1 (ng/mL) at day 0 | 3 | | | | | | Yes |
| IFNG_DAY0 | Interferon Gamma (pg/mL) at day 0 | 3 | | | | | | Yes |
| INTLK1RA_DAY0 | Interleukin 1 Receptor Antagonist (pg/mL) at day 0 | 3 | | | | | | Yes |
| INTLK1A_DAY0 | Interleukin 1 Alpha (pg/mL) at day 0 | 3 | | Y | | | | No |
| INTLK1B_DAY0 | Interleukin 1 Beta (pg/mL) at day 0 | 3 | | Y | | | | No |
| INTLK10_DAY0 | Interleukin 10 (pg/mL) at day 0 | 3 | | | | | | Yes |
| IL122340_DAY0 | Interleukin 12+23 p40 (pg/mL) at day 0 | 3 | | | | | | Yes |
| INTLK12_DAY0 | Interleukin 12 (pg/mL) at day 0 | 3 | | Y | | | | No |
| INTLK13_DAY0 | Interleukin 13 (pg/mL) at day 0 | 3 | | Y | | | | No |
| INTLK15_DAY0 | Interleukin 15 (pg/mL) at day 0 | 3 | | | | | | Yes |
| INTLK16_DAY0 | Interleukin 16 (pg/mL) at day 0 | 3 | | | | | | Yes |
| INTLK17_DAY0 | Interleukin 17 (pg/mL) at day 0 | 3 | | | | | | Yes |
| INTLK2_DAY0 | Interleukin 2 (pg/mL) at day 0 | 3 | | | | | | Yes |

| | | | | | | | | |
|---|---|---|---|---|---|---|---|---|
| INTL2RCU_DAY0 | Interleukin 2 Receptor Alpha (ng/mL) at day 0 | 3 | | | | | | Yes |
| INTLK4_DAY0 | Interleukin 4 (pg/mL) at day 0 | 3 | | Y | | | | No |
| INTLK5_DAY0 | Interleukin 5 (pg/mL) at day 0 | 3 | | Y | | | | No |
| INTLK6_DAY0 | Interleukin 6 (pg/mL) at day 0 | 3 | | | | | | Yes |
| INTLK7_DAY0 | Interleukin 7 (pg/mL) at day 0 | 3 | | | | | | Yes |
| INTLK8_DAY0 | Interleukin 8 (pg/mL) at day 0 | 3 | | | | | | Yes |
| MCP1_DAY0 | Monocyte Chemotactic Protein 1 (pg/mL) at day 0 | 3 | | | | | | Yes |
| MCP4_DAY0 | Monocyte Chemotactic Protein 4 (pg/mL) at day 0 | 3 | | | | | | Yes |
| MDC_DAY0 | Macrophage-Derived Chemokine (pg/mL) at day 0 | 3 | | | | | | Yes |
| MIP1A_DAY0 | Macrophage Inflammatory Protein 1 Alpha (pg/mL) at day 0 | 3 | | | | | | Yes |
| MIP1B_DAY0 | Macrophage Inflammatory Protein 1 Beta (pg/mL) at day 0 | 3 | | | | | | Yes |
| PDL1_DAY0 | Programmed Death Ligand 1 (pg/mL) at day 0 | 3 | | | | | | Yes |
| PRFCU_DAY0 | Perforin (ng/mL) at day 0 | 3 | | | | | | Yes |
| AMYLOIDA_DAY0 | Amyloid A (pg/mL) at day 0 | 3 | | | | | | Yes |

| | | | | | | | | |
|---|---|---|---|---|---|---|---|---|
| SFASL_DAY0 | CD95 Ligand (pg/mL) at day 0 | 3 | | | | | | Yes |
| CCL17_DAY0 | Chemokine (C-C Motif) Ligand 17 (pg/mL) at day 0 | 3 | | | | | | Yes |
| TNFA_DAY0 | Tumor Necrosis Factor Alpha (pg/mL) at day 0 | 3 | | | | | | Yes |
| TNFB_DAY0 | Tumor Necrosis Factor Beta (pg/mL) at day 0 | 3 | | Y | | | | No |
| VCAM1CU_DAY0 | Vascular Cell Adhesion Molecule 1 (ng/mL) at day 0 | 3 | | | | | | Yes |
| VEGF_DAY0 | Vascular Endothelial Growth Factor (pg/mL) at day 0 | 3 | | | | | | Yes |
| GP130_DAY0 | Glycoprotein 130 (pg/mL) at day 0 | 124 | Y | | | | | No |
| INTLK6RA_DAY0 | Interleukin 6 Receptor Alpha (pg/mL) at day 0 | 124 | Y | | | | | No |
| RANTES_DAY0 | Regulated upon Activation, Normal T Cell Expressed and Persumably Secreted (pg/mL) at day 0 | 124 | Y | | | | | No |
| CRP_D0_FCHG_B | C Reactive Protein (mg/L) fold change | 5 | | | | | | Yes |
| CXCL10_D0_FCHG_B | Chemokine (C-X-C Motif) Ligand 10 (pg/mL) fold change | 5 | | | | | | Yes |
| EOTAXIN1_D0_FCHG_B | Eotaxin-1 (pg/mL) fold change | 5 | | | | | | Yes |
| EOTAXIN3_D0_FCHG_B | Eotaxin-3 (pg/mL) fold change | 5 | | | | | | Yes |
| FERRITIN_D0_FCHG_B | Ferritin (ng/mL) fold change | 5 | | | | | | Yes |

| | | | | | | | | |
|---|---|---|---|---|---|---|---|---|
| GMCSF_D0_FCHG_B | Granulocyte Macrophage Colony Stm Factor (pg/mL) fold change | 5 | | Y | | | | No |
| GRZA_D0_FCHG_B | Granzyme A (pg/mL) fold change | 5 | | | | | | Yes |
| GRZAE_D0_FCHG_B | Granzyme A Ella (pg/mL) fold change | 5 | | | | | | Yes |
| GRZB_D0_FCHG_B | Granzyme B (pg/mL) fold change | 5 | | | | | | Yes |
| GRZBE_D0_FCHG_B | Granzyme B Ella (pg/mL) fold change | 5 | | | | | | Yes |
| ICAM1CU_D0_FCHG_B | Intercellular Adhesion Molecule 1 (ng/mL) fold change | 5 | | | | | | Yes |
| IFNG_D0_FCHG_B | Interferon Gamma (pg/mL) fold change | 5 | | | | | | Yes |
| INTLK1RA_D0_FCHG_B | Interleukin 1 Receptor Antagonist (pg/mL) fold change | 5 | | | | | | Yes |
| INTLK1A_D0_FCHG_B | Interleukin 1 Alpha (pg/mL) fold change | 5 | | Y | | | | No |
| INTLK1B_D0_FCHG_B | Interleukin 1 Beta (pg/mL) fold change | 5 | | Y | | | | No |
| INTLK10_D0_FCHG_B | Interleukin 10 (pg/mL) fold change | 5 | | | | | | Yes |
| IL122340_D0_FCHG_B | Interleukin 12+23 p40 (pg/mL) fold change | 5 | | | | | | Yes |
| INTLK12_D0_FCHG_B | Interleukin 12 (pg/mL) fold change | 5 | | Y | | | | No |
| INTLK13_D0_FCHG_B | Interleukin 13 (pg/mL) fold change | 5 | | Y | | | | No |
| INTLK15_D0_FCHG_B | Interleukin 15 (pg/mL) fold change | 5 | | | | | | Yes |

| | | | | | | | | |
|---|---|---|---|---|---|---|---|---|
| INTLK16_D0_FCHG_B | Interleukin 16 (pg/mL) fold change | 5 | | | | | | Yes |
| INTLK17_D0_FCHG_B | Interleukin 17 (pg/mL) fold change | 5 | | | | | | Yes |
| INTLK2_D0_FCHG_B | Interleukin 2 (pg/mL) fold change | 5 | | | | | | Yes |
| INTL2RCU_D0_FCHG_B | Interleukin 2 Receptor Alpha (ng/mL) fold change | 5 | | | | | | Yes |
| INTLK4_D0_FCHG_B | Interleukin 4 (pg/mL) fold change | 5 | | Y | | | | No |
| INTLK5_D0_FCHG_B | Interleukin 5 (pg/mL) fold change | 5 | | Y | | | | No |
| INTLK6_D0_FCHG_B | Interleukin 6 (pg/mL) fold change | 5 | | | | | | Yes |
| INTLK7_D0_FCHG_B | Interleukin 7 (pg/mL) fold change | 5 | | | | | | Yes |
| INTLK8_D0_FCHG_B | Interleukin 8 (pg/mL) fold change | 5 | | | | | | Yes |
| MCP1_D0_FCHG_B | Monocyte Chemotactic Protein 1 (pg/mL) fold change | 5 | | | | | | Yes |
| MCP4_D0_FCHG_B | Monocyte Chemotactic Protein 4 (pg/mL) fold change | 5 | | | | | | Yes |
| MDC_D0_FCHG_B | Macrophage-Derived Chemokine (pg/mL) fold change | 5 | | | | | | Yes |
| MIP1A_D0_FCHG_B | Macrophage Inflammatory Protein 1 Alpha (pg/mL) fold change | 5 | | | | | | Yes |
| MIP1B_D0_FCHG_B | Macrophage Inflammatory Protein 1 Beta (pg/mL) fold change | 5 | | | | | | Yes |

| | | | | | | | | |
|---|---|---|---|---|---|---|---|---|
| PDL1_D0_FCHG_B | Programmed Death Ligand 1 (pg/mL) fold change | 5 | | | | | | Yes |
| PRFCU_D0_FCHG_B | Perforin (ng/mL) fold change | 5 | | | | | | Yes |
| AMYLOIDA_D0_FCHG_B | Amyloid A (pg/mL) fold change | 5 | | | | | | Yes |
| SFASL_D0_FCHG_B | CD95 Ligand (pg/mL) fold change | 5 | | | | | | Yes |
| CCL17_D0_FCHG_B | Chemokine (C-C Motif) Ligand 17 (pg/mL) fold change | 5 | | | | | | Yes |
| TNFA_D0_FCHG_B | Tumor Necrosis Factor Alpha (pg/mL) fold change | 5 | | | | | | Yes |
| TNFB_D0_FCHG_B | Tumor Necrosis Factor Beta (pg/mL) fold change | 5 | | | Y | TNFB_BASELINE | -1 | No |
| VCAM1CU_D0_FCHG_B | Vascular Cell Adhesion Molecule 1 (ng/mL) fold change | 5 | | | | | | Yes |
| VEGF_D0_FCHG_B | Vascular Endothelial Growth Factor (pg/mL) fold change | 5 | | | | | | Yes |
| GP130_D0_FCHG_B | Glycoprotein 130 (pg/mL) fold change | 124 | Y | | | | | No |
| INTLK6RA_D0_FCHG_B | Interleukin 6 Receptor Alpha (pg/mL) fold change | 124 | Y | | | | | No |
| RANTES_D0_FCHG_B | Regulated upon Activation, Normal T Cell Expressed and Persumably Secreted (pg/mL) fold change | 124 | Y | | | | | No |
| CRP_PEAKW4 | C Reactive Protein (mg/L) at peak | 0 | | | Y | CRP_AUCAVAL | 0.910608407 | No |

| | | | | | | | | |
|---|---|---|---|---|---|---|---|---|
| CXCL10_PEAKW4 | Chemokine (C-X-C Motif) Ligand 10 (pg/mL) at peak | 0 | | | | | | Yes |
| EOTAXIN1_PEAKW4 | Eotaxin-1 (pg/mL) at peak | 0 | | | Y | EOTAXIN1_AUCAVAL | 0.938224631 | No |
| EOTAXIN3_PEAKW4 | Eotaxin-3 (pg/mL) at peak | 0 | | | | | | Yes |
| FERRITIN_PEAKW4 | Ferritin (ng/mL) at peak | 0 | | | Y | FERRITIN_AUCAVAL | 0.956487805 | No |
| GMCSF_PEAKW4 | Granulocyte Macrophage Colony Stm Factor (pg/mL) at peak | 0 | | | | | | Yes |
| GRZA_PEAKW4 | Granzyme A (pg/mL) at peak | 0 | | | Y | GRZA_AUCAVAL | 0.942453063 | No |
| GRZAE_PEAKW4 | Granzyme A Ella (pg/mL) at peak | 0 | | | | | | Yes |
| GRZB_PEAKW4 | Granzyme B (pg/mL) at peak | 0 | | | Y | GRZB_AUCAVAL | 0.972448937 | No |
| GRZBE_PEAKW4 | Granzyme B Ella (pg/mL) at peak | 0 | | | Y | GRZBE_AUCAVAL | 0.947209739 | No |
| ICAM1CU_PEAKW4 | Intercellular Adhesion Molecule 1 (ng/mL) at peak | 0 | | | | | | Yes |
| IFNG_PEAKW4 | Interferon Gamma (pg/mL) at peak | 0 | | | Y | IFNG_AUCAVAL | 0.927909132 | No |

| | | | | | | | | |
|---|---|---|---|---|---|---|---|---|
| INTLK1RA_PEAKW4 | Interleukin 1 Receptor Antagonist (pg/mL) at peak | 0 | | | Y | INTLK1RA_AUCAVAL | 0.901851313 | No |
| INTLK1A_PEAKW4 | Interleukin 1 Alpha (pg/mL) at peak | 0 | | Y | | | | No |
| INTLK1B_PEAKW4 | Interleukin 1 Beta (pg/mL) at peak | 0 | | Y | | | | No |
| INTLK10_PEAKW4 | Interleukin 10 (pg/mL) at peak | 0 | | | Y | INTLK10_AUCAVAL | 0.916820907 | No |
| IL122340_PEAKW4 | Interleukin 12+23 p40 (pg/mL) at peak | 0 | | | | | | Yes |
| INTLK12_PEAKW4 | Interleukin 12 (pg/mL) at peak | 0 | | | | | | Yes |
| INTLK13_PEAKW4 | Interleukin 13 (pg/mL) at peak | 0 | | | | | | Yes |
| INTLK15_PEAKW4 | Interleukin 15 (pg/mL) at peak | 0 | | | | | | Yes |
| INTLK16_PEAKW4 | Interleukin 16 (pg/mL) at peak | 0 | | | Y | INTLK16_AUCAVAL | 0.906497099 | No |
| INTLK17_PEAKW4 | Interleukin 17 (pg/mL) at peak | 0 | | | | | | Yes |
| INTLK2_PEAKW4 | Interleukin 2 (pg/mL) at peak | 0 | | | Y | INTLK2_AUCAVAL | 0.960763037 | No |
| INTL2RCU_PEAKW4 | Interleukin 2 Receptor Alpha (ng/mL) at peak | 0 | | | Y | INTL2RCU_AUCAVAL | 0.939795437 | No |
| INTLK4_PEAKW4 | Interleukin 4 (pg/mL) at peak | 0 | | | | | | Yes |

| | | | | | | | | |
|---|---|---|---|---|---|---|---|---|
| INTLK5_PEAKW4 | Interleukin 5 (pg/mL) at peak | 0 | | | Y | INTLK5_AUCAVAL | 0.944301054 | No |
| INTLK6_PEAKW4 | Interleukin 6 (pg/mL) at peak | 0 | | | Y | INTLK6_AUCAVAL | 0.971250796 | No |
| INTLK7_PEAKW4 | Interleukin 7 (pg/mL) at peak | 0 | | | | | | Yes |
| INTLK8_PEAKW4 | Interleukin 8 (pg/mL) at peak | 0 | | | | | | Yes |
| MCP1_PEAKW4 | Monocyte Chemotactic Protein 1 (pg/mL) at peak | 0 | | | | | | Yes |
| MCP4_PEAKW4 | Monocyte Chemotactic Protein 4 (pg/mL) at peak | 0 | | | | | | Yes |
| MDC_PEAKW4 | Macrophage-Derived Chemokine (pg/mL) at peak | 0 | | | Y | MDC_AUCAVAL | 0.956731639 | No |
| MDC_PEAKW4 | Macrophage-Derived Chemokine (pg/mL) at peak | 0 | | | Y | MDC_DAY0 | 0.919202085 | No |
| MIP1A_PEAKW4 | Macrophage Inflammatory Protein 1 Alpha (pg/mL) at peak | 0 | | | | | | Yes |
| MIP1B_PEAKW4 | Macrophage Inflammatory Protein 1 Beta (pg/mL) at peak | 0 | | | | | | Yes |
| PDL1_PEAKW4 | Programmed Death Ligand 1 (pg/mL) at peak | 0 | | | | | | Yes |

| | | | | | | | | |
|---|---|---|---|---|---|---|---|---|
| PRFCU_PEAKW4 | Perforin (ng/mL) at peak | 0 | | | Y | PRFCU_AUCAVAL | 0.917476003 | No |
| AMYLOIDA_PEAKW4 | Amyloid A (pg/mL) at peak | 0 | | | Y | AMYLOIDA_AUCAVAL | 0.939883242 | No |
| SFASL_PEAKW4 | CD95 Ligand (pg/mL) at peak | 0 | | | | | | Yes |
| CCL17_PEAKW4 | Chemokine (C-C Motif) Ligand 17 (pg/mL) at peak | 0 | | | Y | CCL17_AUCAVAL | 0.919075827 | No |
| TNFA_PEAKW4 | Tumor Necrosis Factor Alpha (pg/mL) at peak | 0 | | | | | | Yes |
| TNFB_PEAKW4 | Tumor Necrosis Factor Beta (pg/mL) at peak | 0 | | | | | | Yes |
| VCAM1CU_PEAKW4 | Vascular Cell Adhesion Molecule 1 (ng/mL) at peak | 0 | | | | | | Yes |
| VEGF_PEAKW4 | Vascular Endothelial Growth Factor (pg/mL) at peak | 0 | | | Y | VEGF_AUCAVAL | 0.947758916 | No |
| GP130_PEAKW4 | Glycoprotein 130 (pg/mL) at peak | 124 | Y | | | | | No |
| INTLK6RA_PEAKW4 | Interleukin 6 Receptor Alpha (pg/mL) at peak | 124 | Y | | | | | No |
| RANTES_PEAKW4 | Regulated upon Activation, Normal T Cell Expressed and Persumably Secreted (pg/mL) at peak | 124 | Y | | | | | No |
| CRP_AUCAVAL | C Reactive Protein (mg/L) AUC | 0 | | | | | | Yes |

| | | | | | | | | |
|---|---|---|---|---|---|---|---|---|
| CXCL10_AUCAVAL | Chemokine (C-X-C Motif) Ligand 10 (pg/mL) AUC | 0 | | | | | | Yes |
| EOTAXIN1_AUCAVAL | Eotaxin-1 (pg/mL) AUC | 0 | | | | | | Yes |
| EOTAXIN3_AUCAVAL | Eotaxin-3 (pg/mL) AUC | 0 | | | | | | Yes |
| FERRITIN_AUCAVAL | Ferritin (ng/mL) AUC | 0 | | | | | | Yes |
| GMCSF_AUCAVAL | Granulocyte Macrophage Colony Stm Factor (pg/mL) AUC | 0 | | | | | | Yes |
| GRZA_AUCAVAL | Granzyme A (pg/mL) AUC | 0 | | | | | | Yes |
| GRZAE_AUCAVAL | Granzyme A Ella (pg/mL) AUC | 0 | | | | | | Yes |
| GRZB_AUCAVAL | Granzyme B (pg/mL) AUC | 0 | | | | | | Yes |
| GRZBE_AUCAVAL | Granzyme B Ella (pg/mL) AUC | 0 | | | | | | Yes |
| ICAM1CU_AUCAVAL | Intercellular Adhesion Molecule 1 (ng/mL) AUC | 0 | | | | | | Yes |
| IFNG_AUCAVAL | Interferon Gamma (pg/mL) AUC | 0 | | | | | | Yes |
| INTLK1RA_AUCAVAL | Interleukin 1 Receptor Antagonist (pg/mL) AUC | 0 | | | | | | Yes |
| INTLK1A_AUCAVAL | Interleukin 1 Alpha (pg/mL) AUC | 0 | | | | | | Yes |
| INTLK1A_AUCAVAL | Interleukin 1 Alpha (pg/mL) AUC | 0 | | | | | | Yes |

| | | | | | | | | |
|---|---|---|---|---|---|---|---|---|
| INTLK1B_AUCAVAL | Interleukin 1 Beta (pg/mL) AUC | 0 | | | Y | INTLK1A_AUCAVAL | 1 | No |
| INTLK10_AUCAVAL | Interleukin 10 (pg/mL) AUC | 0 | | | | | | Yes |
| IL122340_AUCAVAL | Interleukin 12+23 p40 (pg/mL) AUC | 0 | | | | | | Yes |
| INTLK12_AUCAVAL | Interleukin 12 (pg/mL) AUC | 0 | | | | | | Yes |
| INTLK13_AUCAVAL | Interleukin 13 (pg/mL) AUC | 0 | | | Y | INTLK1A_AUCAVAL | 0.946687626 | No |
| INTLK15_AUCAVAL | Interleukin 15 (pg/mL) AUC | 0 | | | | | | Yes |
| INTLK16_AUCAVAL | Interleukin 16 (pg/mL) AUC | 0 | | | | | | Yes |
| INTLK17_AUCAVAL | Interleukin 17 (pg/mL) AUC | 0 | | | | | | Yes |
| INTLK2_AUCAVAL | Interleukin 2 (pg/mL) AUC | 0 | | | | | | Yes |
| INTL2RCU_AUCAVAL | Interleukin 2 Receptor Alpha (ng/mL) AUC | 0 | | | | | | Yes |
| INTLK4_AUCAVAL | Interleukin 4 (pg/mL) AUC | 0 | | | | | | Yes |
| INTLK5_AUCAVAL | Interleukin 5 (pg/mL) AUC | 0 | | | | | | Yes |
| INTLK6_AUCAVAL | Interleukin 6 (pg/mL) AUC | 0 | | | | | | Yes |
| INTLK7_AUCAVAL | Interleukin 7 (pg/mL) AUC | 0 | | | | | | Yes |
| INTLK8_AUCAVAL | Interleukin 8 (pg/mL) AUC | 0 | | | | | | Yes |

| | | | | | | | | |
|---|---|---|---|---|---|---|---|---|
| MCP1_AUCAVAL | Monocyte Chemotactic Protein 1 (pg/mL) AUC | 0 | | | | | | Yes |
| MCP4_AUCAVAL | Monocyte Chemotactic Protein 4 (pg/mL) AUC | 0 | | | | | | Yes |
| MDC_AUCAVAL | Macrophage-Derived Chemokine (pg/mL) AUC | 0 | | | Y | MDC_PEAKW4 | 0.956731639 | No |
| MIP1A_AUCAVAL | Macrophage Inflammatory Protein 1 Alpha (pg/mL) AUC | 0 | | | | | | Yes |
| MIP1B_AUCAVAL | Macrophage Inflammatory Protein 1 Beta (pg/mL) AUC | 0 | | | | | | Yes |
| PDL1_AUCAVAL | Programmed Death Ligand 1 (pg/mL) AUC | 0 | | | | | | Yes |
| PRFCU_AUCAVAL | Perforin (ng/mL) AUC | 0 | | | | | | Yes |
| AMYLOIDA_AUCAVAL | Amyloid A (pg/mL) AUC | 0 | | | | | | Yes |
| SFASL_AUCAVAL | CD95 Ligand (pg/mL) AUC | 0 | | | | | | Yes |
| CCL17_AUCAVAL | Chemokine (C-C Motif) Ligand 17 (pg/mL) AUC | 0 | | | | | | Yes |
| TNFA_AUCAVAL | Tumor Necrosis Factor Alpha (pg/mL) AUC | 0 | | | | | | Yes |
| TNFB_AUCAVAL | Tumor Necrosis Factor Beta (pg/mL) AUC | 0 | | | | | | Yes |
| VCAM1CU_AUCAVAL | Vascular Cell Adhesion Molecule 1 (ng/mL) AUC | 0 | | | | | | Yes |

| | | | | | | | | |
|---|---|---|---|---|---|---|---|---|
| VEGF_AUCAVAL | Vascular Endothelial Growth Factor (pg/mL) AUC | 0 | | | | | | Yes |
| GP130_AUCAVAL | Glycoprotein 130 (pg/mL) AUC | 124 | Y | | | | | No |
| INTLK6RA_AUCAVAL | Interleukin 6 Receptor Alpha (pg/mL) AUC | 124 | Y | | | | | No |
| RANTES_AUCAVAL | Regulated upon Activation, Normal T Cell Expressed and Persumably Secreted (pg/mL) AUC | 124 | Y | | | | | No |
| DREFA2FL | Double refractory | 0 | | | | | | Yes |
| PRASCT | Prior Auto. Stem Cell Transplant (ASCT) | 0 | | | | | | Yes |
| PRSCTFL | Prior Stem Cell Transplant (Y/N) | 0 | | | Y | PRASCT | 1 | No |
| PI3KIHFL | Prior PI3K inhibitor | 0 | | | | | | Yes |
| PBTKIHFL | Prior BTK inhibitor | 0 | | | | | | Yes |
| OBIMABFL | Prior Med-Obinutuzumb | 0 | | | | | | Yes |
| ANTAGFL | Prior Med-Anti-CD20 single agent | 0 | | | | | | Yes |
| ALKFL | Prior Med-Alkylating Agent | 0 | | | | | | Yes |
| ANTALKFL | Prior Med-Anti-CD20 + Alkylating Agent | 0 | | | | | | Yes |
| LENDFL | Prior Med-Lenalidomide | 0 | | | | | | Yes |
| POD24_Y | Progression of disease within 24 months | 0 | | | | | | Yes |
| POD24_N | Progression of disease not within 24 months | 0 | | | | | | Yes |

| | | | | | | | | | |
|---|---|---|---|---|---|---|---|---|---|
| BENCAT_LE12 | Prior Med-Bendamustine within 12 months | 0 | | | | | | | Yes |
| BENCAT_GT12 | Prior Med-Bendamustine over 12 months | 0 | | | | | | | Yes |
| HRVSTDY_PROD | Harvest day | 0 | | | | | | | Yes |
| MTVAVAL | Metabolic Tumor Volume Results | 2 | | | | | | | Yes |
| ncart_spd_PEAKVAL | Number of CART peak normalized to SPD | 6 | | | | | | | Yes |
| nnv_spd_BASELINE | Number of Naïve cells normalized to SPD | 25 | | | | | | | Yes |

**Supplementary Table 3. List of post-infusion covariates**

| variable | description |
|---|---|
| ncart_AUCAVAL | ncart AUC |
| CXCL10_PEAKW4 | Chemokine (C-X-C Motif) Ligand 10 (pg/mL) at peak |
| EOTAXIN3_PEAKW4 | Eotaxin-3 (pg/mL) at peak |
| GMCSF_PEAKW4 | Granulocyte Macrophage Colony Stm Factor (pg/mL) at peak |
| GRZAE_PEAKW4 | Granzyme A Ella (pg/mL) at peak |
| ICAM1CU_PEAKW4 | Intercellular Adhesion Molecule 1 (ng/mL) at peak |
| IL122340_PEAKW4 | Interleukin 12+23 p40 (pg/mL) at peak |
| INTLK12_PEAKW4 | Interleukin 12 (pg/mL) at peak |
| INTLK13_PEAKW4 | Interleukin 13 (pg/mL) at peak |
| INTLK15_PEAKW4 | Interleukin 15 (pg/mL) at peak |
| INTLK17_PEAKW4 | Interleukin 17 (pg/mL) at peak |
| INTLK4_PEAKW4 | Interleukin 4 (pg/mL) at peak |
| INTLK7_PEAKW4 | Interleukin 7 (pg/mL) at peak |
| INTLK8_PEAKW4 | Interleukin 8 (pg/mL) at peak |
| MCP1_PEAKW4 | Monocyte Chemotactic Protein 1 (pg/mL) at peak |
| MCP4_PEAKW4 | Monocyte Chemotactic Protein 4 (pg/mL) at peak |
| MIP1A_PEAKW4 | Macrophage Inflammatory Protein 1 Alpha (pg/mL) at peak |
| MIP1B_PEAKW4 | Macrophage Inflammatory Protein 1 Beta (pg/mL) at peak |
| PDL1_PEAKW4 | Programmed Death Ligand 1 (pg/mL) at peak |
| SFASL_PEAKW4 | CD95 Ligand (pg/mL) at peak |
| TNFA_PEAKW4 | Tumor Necrosis Factor Alpha (pg/mL) at peak |
| TNFB_PEAKW4 | Tumor Necrosis Factor Beta (pg/mL) at peak |
| VCAM1CU_PEAKW4 | Vascular Cell Adhesion Molecule 1 (ng/mL) at peak |
| CRP_AUCAVAL | C Reactive Protein (mg/L) AUC |
| CXCL10_AUCAVAL | Chemokine (C-X-C Motif) Ligand 10 (pg/mL) AUC |
| EOTAXIN1_AUCAVAL | Eotaxin-1 (pg/mL) AUC |
| EOTAXIN3_AUCAVAL | Eotaxin-3 (pg/mL) AUC |
| FERRITIN_AUCAVAL | Ferritin (ng/mL) AUC |
| GMCSF_AUCAVAL | Granulocyte Macrophage Colony Stm Factor (pg/mL) AUC |
| GRZA_AUCAVAL | Granzyme A (pg/mL) AUC |
| GRZAE_AUCAVAL | Granzyme A Ella (pg/mL) AUC |
| GRZB_AUCAVAL | Granzyme B (pg/mL) AUC |
| GRZBE_AUCAVAL | Granzyme B Ella (pg/mL) AUC |
| ICAM1CU_AUCAVAL | Intercellular Adhesion Molecule 1 (ng/mL) AUC |
| IFNG_AUCAVAL | Interferon Gamma (pg/mL) AUC |
| INTLK1RA_AUCAVAL | Interleukin 1 Receptor Antagonist (pg/mL) AUC |
| INTLK1A_AUCAVAL | Interleukin 1 Alpha (pg/mL) AUC |

| | |
|---|---|
| INTLK1A_AUCAVAL | Interleukin 1 Alpha (pg/mL) AUC |
| INTLK10_AUCAVAL | Interleukin 10 (pg/mL) AUC |
| IL122340_AUCAVAL | Interleukin 12+23 p40 (pg/mL) AUC |
| INTLK12_AUCAVAL | Interleukin 12 (pg/mL) AUC |
| INTLK15_AUCAVAL | Interleukin 15 (pg/mL) AUC |
| INTLK16_AUCAVAL | Interleukin 16 (pg/mL) AUC |
| INTLK17_AUCAVAL | Interleukin 17 (pg/mL) AUC |
| INTLK2_AUCAVAL | Interleukin 2 (pg/mL) AUC |
| INTL2RCU_AUCAVAL | Interleukin 2 Receptor Alpha (ng/mL) AUC |
| INTLK4_AUCAVAL | Interleukin 4 (pg/mL) AUC |
| INTLK5_AUCAVAL | Interleukin 5 (pg/mL) AUC |
| INTLK6_AUCAVAL | Interleukin 6 (pg/mL) AUC |
| INTLK7_AUCAVAL | Interleukin 7 (pg/mL) AUC |
| INTLK8_AUCAVAL | Interleukin 8 (pg/mL) AUC |
| MCP1_AUCAVAL | Monocyte Chemotactic Protein 1 (pg/mL) AUC |
| MCP4_AUCAVAL | Monocyte Chemotactic Protein 4 (pg/mL) AUC |
| MIP1A_AUCAVAL | Macrophage Inflammatory Protein 1 Alpha (pg/mL) AUC |
| MIP1B_AUCAVAL | Macrophage Inflammatory Protein 1 Beta (pg/mL) AUC |
| PDL1_AUCAVAL | Programmed Death Ligand 1 (pg/mL) AUC |
| PRFCU_AUCAVAL | Perforin (ng/mL) AUC |
| AMYLOIDA_AUCAVAL | Amyloid A (pg/mL) AUC |
| SFASL_AUCAVAL | CD95 Ligand (pg/mL) AUC |
| CCL17_AUCAVAL | Chemokine (C-C Motif) Ligand 17 (pg/mL) AUC |
| TNFA_AUCAVAL | Tumor Necrosis Factor Alpha (pg/mL) AUC |
| TNFB_AUCAVAL | Tumor Necrosis Factor Beta (pg/mL) AUC |
| VCAM1CU_AUCAVAL | Vascular Cell Adhesion Molecule 1 (ng/mL) AUC |
| VEGF_AUCAVAL | Vascular Endothelial Growth Factor (pg/mL) AUC |
| ncart_spd_PEAKVAL | Number of CART peak normalized to SPD |
| nnv_spd_BASELINE | Number of Naïve cells normalized to SPD |

**Supplementary Table 4. Multivariate analysis of combined pre-treatment and post-infusion covariates**

| | PFS | | | Ongoing Response | | | NTx G3+ w/o toci or steroid | | | CRS G3+ w/o toci or steroid | | |
|---|---|---|---|---|---|---|---|---|---|---|---|---|
| rank | PARAM | std.vi.mean | Direction | PARAM | vi.std.mean | Direction | PARAM | vi.std.mean | Direction | PARAM | vi.std.mean | Direction |
| 1 | TNFA_DAY0 | 100 | - | TNFA_DAY0 | 100 | - | GMCSF_PEAKW4 | 100 | + | INTLK6_AUCAVAL | 100 | + |
| 2 | IL122340_DAY0 | 46.7 | - | IL122340_DAY0 | 78 | - | GMCSF_AUCAVAL | 70.3 | + | TNFA_PEAKW4 | 33.3 | + |
| 3 | HGB_BASELINE | 21.8 | + | NNV_BASELINE | 51.5 | + | MCP1_PEAKW4 | 20.9 | + | INTLK2_AUCAVAL | 15.6 | + |
| 4 | INTL2RCU_DAY0 | 20.9 | - | INTL2RCU_DAY0 | 34.8 | - | INTLK6_AUCAVAL | 16.5 | + | NCM_BASELINE | 11.8 | + |
| 5 | NNV_BASELINE | 20.1 | + | HGB_BASELINE | 12.6 | + | INTLK2_AUCAVAL | 13.7 | + | ncart_spd_PEAKVAL | 11.7 | + |
| 6 | tocilizumab_use_n | 17.1 | + | INTLK16_DAY0 | 11.3 | - | INTLK15_PEAKW4 | 9.6 | + | CXCL10_PEAKW4 | 7.3 | + |
| 7 | MDC_DAY0 | 16.5 | - | score_n | 10.7 | - | INTLK10_AUCAVAL | 5.4 | + | VEGF_BASELINE | 2.7 | + |
| 8 | CCL17_BASELINE | 13.1 | - | INTLK8_BASELINE | 9.5 | + | NNCM_BASELINE | 5.2 | + | PEF_BASELINE | 2.4 | - |
| 9 | INTLK16_DAY0 | 12.2 | - | IL122340_AUCAVAL | 9 | - | ncart_AUCAVAL | 4.8 | + | NNCM_BASELINE | 1.8 | + |
| 10 | HGB_DAY0 | 12 | + | INTLK15_BASELINE | 8.1 | - | MIP1B_PEAKW4 | 2.3 | + | GRZBE_AUCAVAL | 1 | + |
| 11 | ncart_AUCAVAL | 11.5 | + | INTLK16_BASELINE | 6.6 | - | BICARB_BASELINE | 1.3 | + | PEEM_BASELINE | 0.6 | - |
| 12 | PEEM_BASELINE | 11.4 | - | CXCL10_DAY0 | 5.9 | - | AST_DAY0 | 0.9 | + | IFNG_AUCAVAL | 0.3 | + |
| 13 | MDC_BASELINE | 10.9 | - | MCP4_BASELINE | 5 | + | INTL2RCU_AUCAVAL | 0.9 | + | INTLK8_D0_FCHG_B | 0.2 | - |
| 14 | TNFA_BASELINE | 10.4 | - | HGB_DAY0 | 5 | + | NCD4_BASELINE | 0.7 | + | INTLK4_PEAKW4 | 0 | + |
| 15 | MTVAVAL | 10.3 | - | FERRITIN_BASELINE | 5 | - | GRZB_AUCAVAL | 0.7 | + | INTLK4_AUCAVAL | 0 | + |
| 16 | IL122340_BASELINE | 7.3 | - | AGE | 3.7 | - | CD19HS01 | 0.6 | + | CXCL10_DAY0 | -0.1 | + |
| 17 | FERRITIN_BASELINE | 6.1 | - | ALT_DAY0 | 3.4 | + | NNV_BASELINE | 0.6 | + | INTLK8_PEAKW4 | -0.2 | + |
| 18 | score_n | 4.8 | - | PLAT_BASELINE | 3.4 | + | NCD3_BASELINE | 0.4 | + | MIP1A_PEAKW4 | -0.4 | + |
| 19 | NNCM_BASELINE | 4.4 | + | VEGF_AUCAVAL | 3 | + | INTLK4_PEAKW4 | 0.4 | + | VCAM1CU_BASELINE | -0.8 | + |
| 20 | PHOS_DAY0 | 4.2 | - | VCAM1CU_BASELINE | 2.3 | - | IL122340_AUCAVAL | 0.2 | - | TNFA_DAY0 | -0.8 | + |
| 21 | INTLK16_BASELINE | 3.8 | - | GRZB_AUCAVAL | 2.2 | + | PRFCU_D0_FCHG_B | 0.1 | - | VCAM1CU_PEAKW4 | -0.9 | + |
| 22 | MIP1B_BASELINE | 3.1 | - | MCP4_DAY0 | 1.7 | + | MCP1_AUCAVAL | 0.1 | + | ncart_AUCAVAL | -2.3 | + |
| 23 | bulkdis_n | 2.7 | - | INTLK1RA_AUCAVAL | 1.4 | + | IFNG_AUCAVAL | 0 | + | PCM_BASELINE | -3.2 | + |
| 24 | CRP_BASELINE | 2.3 | - | EOTAXIN1_D0_FCHG_B | 1.2 | - | FERRITIN_BASELINE | -0.1 | - | GMCSF_PEAKW4 | -4.7 | + |
| 25 | BTBURC01 | 2 | - | CL_D0_FCHG_B | 0.9 | + | ncart_spd_PEAKVAL | -0.1 | + | GRZAE_PEAKW4 | -5.6 | + |
| 26 | NEM_BASELINE | 1.7 | - | VCAM1CU_AUCAVAL | 0.5 | - | AGE | -0.1 | + | | | |
| 27 | NEEM_BASELINE | 1.6 | - | ICAM1CU_DAY0 | 0.2 | - | | | | | | |
| 28 | INTLK7_PEAKW4 | 1.4 | + | ICAM1CU_BASELINE | 0.2 | - | | | | | | |
| 29 | INTLK8_BASELINE | 1.3 | + | INTLK6_AUCAVAL | 0.1 | + | | | | | | |
| 30 | ALT_DAY0 | 0.9 | + | INTLK6_BASELINE | 0 | + | | | | | | |
| 31 | nnv_spd_BASELINE | 0.7 | + | PRASCT | 0 | + | | | | | | |
| 32 | UREAN_D0_FCHG_B | 0.7 | - | URATE_DAY0 | -0.6 | - | | | | | | |
| 33 | URATE_DAY0 | 0.6 | - | | | | | | | | | |
| 34 | INTLK16_AUCAVAL | 0.1 | - | | | | | | | | | |
| 35 | INTLK10_DAY0 | 0 | - | | | | | | | | | |

PFS, Progression-free survival; NTx, Neurologic events; CRS, Cytokine release syndrome; Toci, Tocilizumab.

**Supplementary Table 5. Nanostring IO360 signatures and Immunosign 21 (IS21) values for association with ongoing response**

| Gene expression signature | pvalue | log2FC |
|---|---|---|
| TIS | 0.117352 | -0.275167 |
| APM | 0.52324 | -0.081861 |
| APM-Loss | 0.851471 | 0.079201 |
| Apoptosis | 0.670233 | -0.024903 |
| B-Cells | 0.809136 | -0.033625 |
| B7-H3 | 0.329942 | 0.23535 |
| CD8-T-Cells | 0.932182 | 0.046847 |
| Cytotoxic-Cells | 0.198455 | -0.330299 |
| Cytotoxicity | 0.117352 | -0.345194 |
| DC | 0.645548 | -0.110667 |
| Endothelial-Cells | 0.645548 | 0.086167 |
| Exhausted-CD8 | 0.313107 | -0.173826 |
| Glycolytic-Activity | 0.972472 | 0.007931 |
| Hypermutation | 0.506008 | -0.240892 |
| Hypoxia | 0.313107 | -0.120931 |
| IFN-Downstream | 0.463197 | -0.157306 |
| IFNG-IO360 | 0.003063 | -0.795868 |
| Immunoproteasome | 0.573822 | -0.056938 |
| Inflammatory-Chemokines | 0.38379 | -0.262715 |
| JAKSTAT-Loss | 0.352661 | 0.223216 |
| Lymphoid | 0.720635 | -0.019535 |
| Macrophages | 0.851471 | -0.069125 |
| MAGEs | 0.905162 | -0.000662 |
| Mast-Cells | 0.388295 | -0.270836 |
| MHC2 | 0.292602 | -0.203646 |
| MMR-Loss | 0.107112 | 0.192823 |
| MSI-Predictor | 0.091151 | -0.039757 |
| Myeloid | 0.986421 | -0.072139 |
| Myeloid-Inflammation | 0.746302 | 0.082244 |
| Neutrophils | 0.720635 | -0.187715 |
| NK-CD56dim | 0.186568 | -0.329021 |
| NK-Cells | 0.69527 | -0.090403 |
| PD-1 | 0.720635 | 0.248188 |
| PD-L1 | 1 | -0.027333 |

| | | |
|---|---|---|
| PD-L2 | 0.905162 | 0.046924 |
| Proliferation | 0.137874 | -0.320819 |
| Stroma | 0.38379 | 0.253764 |
| T-Cells | 0.506008 | 0.202188 |
| TGF-Beta | 0.281118 | 0.139458 |
| IS21 | 0.690015 | -0.402778 |

**Supplementary Table 6. Enrichment scores evaluated using RNAseq dataset for association with ongoing response**

| Signatures | FDR | ES _ratio |
|---|---|---|
| STAT1_19272155 | 0.015796075 | 0.972350261 |
| Chemokine12_score | 0.023522955 | 0.965709615 |
| Interferon_19272155 | 0.034935176 | 0.96308741 |
| Cytotoxic cells | 0.035824249 | 0.964547899 |
| Module3_IFN_score | 0.04098717 | 0.972156832 |
| IFN_21978456 | 0.043164285 | 0.966575515 |
| ICRscore | 0.072242044 | 0.975758321 |
| Minterferon_Cluster_21214954 | 0.073658348 | 0.984574443 |
| Interferon_Cluster_21214954 | 0.100401334 | 0.981214991 |
| CD103neg_mean_25446897 | 0.113525214 | 0.975674074 |
| TAMsurr_score | 0.139933482 | 0.967009456 |
| Inhibitory | 0.150376349 | 0.987222995 |
| Module5_TcellBcell_score | 0.162037366 | 0.988933403 |
| Module11_Prolif_score | 0.17527999 | 0.979300311 |
| TIS_genes | 0.182085556 | 0.985339332 |
| ISG_RS | 0.200663323 | 0.984863771 |
| STAT1_score | 0.206071154 | 0.990887621 |
| PD1_PDL1_score | 0.209961075 | 0.976813411 |
| Exaustion | 0.210018885 | 0.979562348 |
| IL2_score_21050467 | 0.210597328 | 1.006337592 |
| IFNG_GS | 0.233248943 | 0.994120078 |
| Th1 cells | 0.248030029 | 0.973045094 |
| IL13_score_21050467 | 0.255387194 | 0.993050889 |
| Co-stimulatory | 0.29658677 | 1.010845088 |
| NHI_5gene_score | 0.305041618 | 0.97991582 |
| IGG_Cluster_21214954 | 0.332415847 | 0.993433237 |
| Mast cells | 0.33334021 | 0.975966678 |
| IL12_score_21050467 | 0.346673777 | 0.991743216 |
| Immune_Score | 0.365077967 | 0.994426242 |
| MHC1_21978456 | 0.367724401 | 0.96611976 |
| MHC_I_19272155 | 0.367724401 | 0.96611976 |
| TGFB_score_21050467 | 0.383223235 | 1.009680323 |
| Neutrophils | 0.391029189 | 0.986292421 |
| IFNG_score_21050467 | 0.462927744 | 0.996479647 |

| | | |
|---|---|---|
| NK CD56dim cells | 0.468408649 | 1.008392949 |
| Immune_cell_Cluster_21214954 | 0.482014188 | 0.994525941 |
| MHC2_21978456 | 0.577931037 | 0.98356525 |
| CD8Exaustion | 0.614976439 | 0.985731021 |
| Bcell_receptors_score | 0.615983737 | 0.995638735 |
| CD45 | 0.623708748 | 0.9987023 |
| CD103pos_mean_25446897 | 0.62546691 | 0.994843357 |
| Troester_WoundSig_19887484 | 0.642303108 | 1.003617188 |
| IL4_score_21050467 | 0.66388871 | 0.998650317 |
| Macrophages | 0.68543736 | 0.991236483 |
| Bcell_21978456 | 0.725258298 | 0.992724421 |
| T-cells | 0.756147376 | 1.005905206 |
| MHC_II_19272155 | 0.776687446 | 1.002948751 |
| Exhausted CD8 | 0.802287172 | 1.004715779 |
| IL8_21978456 | 0.812656702 | 1.00781775 |
| CD8 T cells | 0.864125645 | 0.99423134 |
| Tcell_receptors_score | 0.886269998 | 0.997124909 |
| DC | 0.908566168 | 0.994205458 |
| Tcell_21978456 | 0.909106417 | 1.000975653 |
| B-cells | 0.921094097 | 0.998835478 |
| Module4_TcellBcell_score | 0.946395397 | 0.999473825 |
| Treg | 0.990573186 | 0.999727395 |
